# A detailed map of Higgs boson interactions by the ATLAS experiment ten years after the discovery

**DOI:** 10.1038/s41586-022-04893-w

**Published:** 2022-07-04

**Authors:** The ATLAS Collaboration, G. Aad, B. Abbott, D. C. Abbott, K. Abeling, S. H. Abidi, A. Aboulhorma, H. Abramowicz, H. Abreu, Y. Abulaiti, A. C. Abusleme Hoffman, B. S. Acharya, B. Achkar, L. Adam, C. Adam Bourdarios, L. Adamczyk, L. Adamek, S. V. Addepalli, J. Adelman, A. Adiguzel, S. Adorni, T. Adye, A. A. Affolder, Y. Afik, M. N. Agaras, J. Agarwala, A. Aggarwal, C. Agheorghiesei, J. A. Aguilar-Saavedra, A. Ahmad, F. Ahmadov, W. S. Ahmed, S. Ahuja, X. Ai, G. Aielli, I. Aizenberg, M. Akbiyik, T. P. A. Åkesson, A. V. Akimov, K. Al Khoury, G. L. Alberghi, J. Albert, P. Albicocco, M. J. Alconada Verzini, S. Alderweireldt, M. Aleksa, I. N. Aleksandrov, C. Alexa, T. Alexopoulos, A. Alfonsi, F. Alfonsi, M. Alhroob, B. Ali, S. Ali, M. Aliev, G. Alimonti, C. Allaire, B. M. M. Allbrooke, P. P. Allport, A. Aloisio, F. Alonso, C. Alpigiani, E. Alunno Camelia, M. Alvarez Estevez, M. G. Alviggi, Y. Amaral Coutinho, A. Ambler, C. Amelung, C. G. Ames, D. Amidei, S. P. Amor Dos Santos, S. Amoroso, K. R. Amos, C. S. Amrouche, V. Ananiev, C. Anastopoulos, N. Andari, T. Andeen, J. K. Anders, S. Y. Andrean, A. Andreazza, S. Angelidakis, A. Angerami, A. V. Anisenkov, A. Annovi, C. Antel, M. T. Anthony, E. Antipov, M. Antonelli, D. J. A. Antrim, F. Anulli, M. Aoki, J. A. Aparisi Pozo, M. A. Aparo, L. Aperio Bella, C. Appelt, N. Aranzabal, V. Araujo Ferraz, C. Arcangeletti, A. T. H. Arce, E. Arena, J-F. Arguin, S. Argyropoulos, J.-H. Arling, A. J. Armbruster, O. Arnaez, H. Arnold, Z. P. Arrubarrena Tame, G. Artoni, H. Asada, K. Asai, S. Asai, N. A. Asbah, E. M. Asimakopoulou, J. Assahsah, K. Assamagan, R. Astalos, R. J. Atkin, M. Atkinson, N. B. Atlay, H. Atmani, P. A. Atmasiddha, K. Augsten, S. Auricchio, A. D. Auriol, V. A. Austrup, G. Avner, G. Avolio, K. Axiotis, M. K. Ayoub, G. Azuelos, D. Babal, H. Bachacou, K. Bachas, A. Bachiu, F. Backman, A. Badea, P. Bagnaia, M. Bahmani, A. J. Bailey, V. R. Bailey, J. T. Baines, C. Bakalis, O. K. Baker, P. J. Bakker, E. Bakos, D. Bakshi Gupta, S. Balaji, R. Balasubramanian, E. M. Baldin, P. Balek, E. Ballabene, F. Balli, L. M. Baltes, W. K. Balunas, J. Balz, E. Banas, M. Bandieramonte, A. Bandyopadhyay, S. Bansal, L. Barak, E. L. Barberio, D. Barberis, M. Barbero, G. Barbour, K. N. Barends, T. Barillari, M.-S. Barisits, J. Barkeloo, T. Barklow, R. M. Barnett, P. Baron, D. A. Baron Moreno, A. Baroncelli, G. Barone, A. J. Barr, L. Barranco Navarro, F. Barreiro, J. Barreiro Guimarães da Costa, U. Barron, M. G. Barros Teixeira, S. Barsov, F. Bartels, R. Bartoldus, A. E. Barton, P. Bartos, A. Basalaev, A. Basan, M. Baselga, I. Bashta, A. Bassalat, M. J. Basso, C. R. Basson, R. L. Bates, S. Batlamous, J. R. Batley, B. Batool, M. Battaglia, M. Bauce, P. Bauer, A. Bayirli, J. B. Beacham, T. Beau, P. H. Beauchemin, F. Becherer, P. Bechtle, H. P. Beck, K. Becker, C. Becot, A. J. Beddall, V. A. Bednyakov, C. P. Bee, L. J. Beemster, T. A. Beermann, M. Begalli, M. Begel, A. Behera, J. K. Behr, C. Beirao Da Cruz E Silva, J. F. Beirer, F. Beisiegel, M. Belfkir, G. Bella, L. Bellagamba, A. Bellerive, P. Bellos, K. Beloborodov, K. Belotskiy, N. L. Belyaev, D. Benchekroun, F. Bendebba, Y. Benhammou, D. P. Benjamin, M. Benoit, J. R. Bensinger, S. Bentvelsen, L. Beresford, M. Beretta, D. Berge, E. Bergeaas Kuutmann, N. Berger, B. Bergmann, J. Beringer, S. Berlendis, G. Bernardi, C. Bernius, F. U. Bernlochner, T. Berry, P. Berta, A. Berthold, I. A. Bertram, O. Bessidskaia Bylund, S. Bethke, A. Betti, A. J. Bevan, M. Bhamjee, S. Bhatta, D. S. Bhattacharya, P. Bhattarai, V. S. Bhopatkar, R. Bi, R. Bi, R. M. Bianchi, O. Biebel, R. Bielski, M. Biglietti, T. R. V. Billoud, M. Bindi, A. Bingul, C. Bini, S. Biondi, A. Biondini, C. J. Birch-sykes, G. A. Bird, M. Birman, T. Bisanz, D. Biswas, A. Bitadze, K. Bjørke, I. Bloch, C. Blocker, A. Blue, U. Blumenschein, J. Blumenthal, G. J. Bobbink, V. S. Bobrovnikov, M. Boehler, D. Bogavac, A. G. Bogdanchikov, C. Bohm, V. Boisvert, P. Bokan, T. Bold, M. Bomben, M. Bona, M. Boonekamp, C. D. Booth, A. G. Borbély, H. M. Borecka-Bielska, L. S. Borgna, G. Borissov, D. Bortoletto, D. Boscherini, M. Bosman, J. D. Bossio Sola, K. Bouaouda, J. Boudreau, E. V. Bouhova-Thacker, D. Boumediene, R. Bouquet, A. Boveia, J. Boyd, D. Boye, I. R. Boyko, J. Bracinik, N. Brahimi, G. Brandt, O. Brandt, F. Braren, B. Brau, J. E. Brau, W. D. Breaden Madden, K. Brendlinger, R. Brener, L. Brenner, R. Brenner, S. Bressler, B. Brickwedde, D. Britton, D. Britzger, I. Brock, G. Brooijmans, W. K. Brooks, E. Brost, P. A. Bruckman de Renstrom, B. Brüers, D. Bruncko, A. Bruni, G. Bruni, M. Bruschi, N. Bruscino, L. Bryngemark, T. Buanes, Q. Buat, P. Buchholz, A. G. Buckley, I. A. Budagov, M. K. Bugge, O. Bulekov, B. A. Bullard, S. Burdin, C. D. Burgard, A. M. Burger, B. Burghgrave, J. T. P. Burr, C. D. Burton, J. C. Burzynski, E. L. Busch, V. Büscher, P. J. Bussey, J. M. Butler, C. M. Buttar, J. M. Butterworth, W. Buttinger, C. J. Buxo Vazquez, A. R. Buzykaev, G. Cabras, S. Cabrera Urbán, D. Caforio, H. Cai, Y. Cai, V. M. M. Cairo, O. Cakir, N. Calace, P. Calafiura, G. Calderini, P. Calfayan, G. Callea, L. P. Caloba, D. Calvet, S. Calvet, T. P. Calvet, M. Calvetti, R. Camacho Toro, S. Camarda, D. Camarero Munoz, P. Camarri, M. T. Camerlingo, D. Cameron, C. Camincher, M. Campanelli, A. Camplani, V. Canale, A. Canesse, M. Cano Bret, J. Cantero, Y. Cao, F. Capocasa, M. Capua, A. Carbone, R. Cardarelli, J. C. J. Cardenas, F. Cardillo, T. Carli, G. Carlino, B. T. Carlson, E. M. Carlson, L. Carminati, M. Carnesale, S. Caron, E. Carquin, S. Carrá, G. Carratta, F. Carrio Argos, J. W. S. Carter, T. M. Carter, M. P. Casado, A. F. Casha, E. G. Castiglia, F. L. Castillo, L. Castillo Garcia, V. Castillo Gimenez, N. F. Castro, A. Catinaccio, J. R. Catmore, V. Cavaliere, N. Cavalli, V. Cavasinni, E. Celebi, F. Celli, M. S. Centonze, K. Cerny, A. S. Cerqueira, A. Cerri, L. Cerrito, F. Cerutti, A. Cervelli, S. A. Cetin, Z. Chadi, D. Chakraborty, M. Chala, J. Chan, W. S. Chan, W. Y. Chan, J. D. Chapman, B. Chargeishvili, D. G. Charlton, T. P. Charman, M. Chatterjee, S. Chekanov, S. V. Chekulaev, G. A. Chelkov, A. Chen, B. Chen, B. Chen, C. Chen, H. Chen, H. Chen, J. Chen, J. Chen, S. Chen, S. J. Chen, X. Chen, X. Chen, Y. Chen, C. L. Cheng, H. C. Cheng, A. Cheplakov, E. Cheremushkina, E. Cherepanova, R. Cherkaoui El Moursli, E. Cheu, K. Cheung, L. Chevalier, V. Chiarella, G. Chiarelli, G. Chiodini, A. S. Chisholm, A. Chitan, Y. H. Chiu, M. V. Chizhov, K. Choi, A. R. Chomont, Y. Chou, E. Y. S. Chow, T. Chowdhury, L. D. Christopher, K. L. Chu, M. C. Chu, X. Chu, J. Chudoba, J. J. Chwastowski, D. Cieri, K. M. Ciesla, V. Cindro, A. Ciocio, F. Cirotto, Z. H. Citron, M. Citterio, D. A. Ciubotaru, B. M. Ciungu, A. Clark, P. J. Clark, J. M. Clavijo Columbie, S. E. Clawson, C. Clement, J. Clercx, L. Clissa, Y. Coadou, M. Cobal, A. Coccaro, R. F. Coelho Barrue, R. Coelho Lopes De Sa, S. Coelli, H. Cohen, A. E. C. Coimbra, B. Cole, J. Collot, P. Conde Muiño, M. P. Connell, S. H. Connell, I. A. Connelly, E. I. Conroy, F. Conventi, H. G. Cooke, A. M. Cooper-Sarkar, F. Cormier, L. D. Corpe, M. Corradi, E. E. Corrigan, F. Corriveau, A. Cortes-Gonzalez, M. J. Costa, F. Costanza, D. Costanzo, B. M. Cote, G. Cowan, J. W. Cowley, K. Cranmer, S. Crépé-Renaudin, F. Crescioli, M. Cristinziani, M. Cristoforetti, V. Croft, G. Crosetti, A. Cueto, T. Cuhadar Donszelmann, H. Cui, Z. Cui, A. R. Cukierman, W. R. Cunningham, F. Curcio, P. Czodrowski, M. M. Czurylo, M. J. Da Cunha Sargedas De Sousa, J. V. Da Fonseca Pinto, C. Da Via, W. Dabrowski, T. Dado, S. Dahbi, T. Dai, C. Dallapiccola, M. Dam, G. D’amen, V. D’Amico, J. Damp, J. R. Dandoy, M. F. Daneri, M. Danninger, V. Dao, G. Darbo, S. Darmora, S. J. Das, A. Dattagupta, S. D’Auria, C. David, T. Davidek, D. R. Davis, B. Davis-Purcell, I. Dawson, K. De, R. De Asmundis, M. De Beurs, S. De Castro, N. De Groot, P. de Jong, H. De la Torre, A. De Maria, A. De Salvo, U. De Sanctis, A. De Santo, J. B. De Vivie De Regie, D. V. Dedovich, J. Degens, A. M. Deiana, F. Del Corso, J. Del Peso, F. Del Rio, F. Deliot, C. M. Delitzsch, M. Della Pietra, D. Della Volpe, A. Dell’Acqua, L. Dell’Asta, M. Delmastro, P. A. Delsart, S. Demers, M. Demichev, S. P. Denisov, L. D’Eramo, D. Derendarz, F. Derue, P. Dervan, K. Desch, K. Dette, C. Deutsch, P. O. Deviveiros, F. A. Di Bello, A. Di Ciaccio, L. Di Ciaccio, A. Di Domenico, C. Di Donato, A. Di Girolamo, G. Di Gregorio, A. Di Luca, B. Di Micco, R. Di Nardo, C. Diaconu, F. A. Dias, T. Dias Do Vale, M. A. Diaz, F. G. Diaz Capriles, M. Didenko, E. B. Diehl, L. Diehl, S. Díez Cornell, C. Diez Pardos, C. Dimitriadi, A. Dimitrievska, W. Ding, J. Dingfelder, I.-M. Dinu, S. J. Dittmeier, F. Dittus, F. Djama, T. Djobava, J. I. Djuvsland, D. Dodsworth, C. Doglioni, J. Dolejsi, Z. Dolezal, M. Donadelli, B. Dong, J. Donini, A. D’Onofrio, M. D’Onofrio, J. Dopke, A. Doria, M. T. Dova, A. T. Doyle, M. A. Draguet, E. Drechsler, E. Dreyer, I. Drivas-koulouris, A. S. Drobac, D. Du, T. A. du Pree, F. Dubinin, M. Dubovsky, E. Duchovni, G. Duckeck, O. A. Ducu, D. Duda, A. Dudarev, M. D’uffizi, L. Duflot, M. Dührssen, C. Dülsen, A. E. Dumitriu, M. Dunford, S. Dungs, K. Dunne, A. Duperrin, H. Duran Yildiz, M. Düren, A. Durglishvili, B. L. Dwyer, G. I. Dyckes, M. Dyndal, S. Dysch, B. S. Dziedzic, Z. O. Earnshaw, B. Eckerova, M. G. Eggleston, E. Egidio Purcino De Souza, L. F. Ehrke, G. Eigen, K. Einsweiler, T. Ekelof, P. A. Ekman, Y. El Ghazali, H. El Jarrari, A. El Moussaouy, V. Ellajosyula, M. Ellert, F. Ellinghaus, A. A. Elliot, N. Ellis, J. Elmsheuser, M. Elsing, D. Emeliyanov, A. Emerman, Y. Enari, I. Ene, S. Epari, J. Erdmann, A. Ereditato, P. A. Erland, M. Errenst, M. Escalier, C. Escobar, E. Etzion, G. Evans, H. Evans, M. O. Evans, A. Ezhilov, S. Ezzarqtouni, F. Fabbri, L. Fabbri, G. Facini, V. Fadeyev, R. M. Fakhrutdinov, S. Falciano, P. J. Falke, S. Falke, J. Faltova, Y. Fan, Y. Fang, G. Fanourakis, M. Fanti, M. Faraj, A. Farbin, A. Farilla, T. Farooque, S. M. Farrington, F. Fassi, D. Fassouliotis, M. Faucci Giannelli, W. J. Fawcett, L. Fayard, O. L. Fedin, G. Fedotov, M. Feickert, L. Feligioni, A. Fell, D. E. Fellers, C. Feng, M. Feng, M. J. Fenton, A. B. Fenyuk, L. Ferencz, S. W. Ferguson, J. Pretel, J. Ferrando, A. Ferrari, P. Ferrari, R. Ferrari, D. Ferrere, C. Ferretti, F. Fiedler, A. Filipčič, E. K. Filmer, F. Filthaut, M. C. N. Fiolhais, L. Fiorini, F. Fischer, W. C. Fisher, T. Fitschen, I. Fleck, P. Fleischmann, T. Flick, L. Flores, M. Flores, L. R. Flores Castillo, F. M. Follega, N. Fomin, J. H. Foo, B. C. Forland, A. Formica, A. C. Forti, E. Fortin, A. W. Fortman, M. G. Foti, L. Fountas, D. Fournier, H. Fox, P. Francavilla, S. Francescato, M. Franchini, S. Franchino, D. Francis, L. Franco, L. Franconi, M. Franklin, G. Frattari, A. C. Freegard, P. M. Freeman, W. S. Freund, N. Fritzsche, A. Froch, D. Froidevaux, J. A. Frost, Y. Fu, M. Fujimoto, E. Fullana Torregrosa, J. Fuster, A. Gabrielli, A. Gabrielli, P. Gadow, G. Gagliardi, L. G. Gagnon, G. E. Gallardo, E. J. Gallas, B. J. Gallop, R. Gamboa Goni, K. K. Gan, S. Ganguly, J. Gao, Y. Gao, F. M. Garay Walls, B. Garcia, C. García, J. E. García Navarro, J. A. García Pascual, M. Garcia-Sciveres, R. W. Gardner, D. Garg, R. B. Garg, S. Gargiulo, C. A. Garner, V. Garonne, S. J. Gasiorowski, P. Gaspar, G. Gaudio, V. Gautam, P. Gauzzi, I. L. Gavrilenko, A. Gavrilyuk, C. Gay, G. Gaycken, E. N. Gazis, A. A. Geanta, C. M. Gee, J. Geisen, M. Geisen, C. Gemme, M. H. Genest, S. Gentile, S. George, W. F. George, T. Geralis, L. O. Gerlach, P. Gessinger-Befurt, M. Ghasemi Bostanabad, M. Ghneimat, A. Ghosal, A. Ghosh, A. Ghosh, B. Giacobbe, S. Giagu, N. Giangiacomi, P. Giannetti, A. Giannini, S. M. Gibson, M. Gignac, D. T. Gil, A. K. Gilbert, B. J. Gilbert, D. Gillberg, G. Gilles, N. E. K. Gillwald, L. Ginabat, D. M. Gingrich, M. P. Giordani, P. F. Giraud, G. Giugliarelli, D. Giugni, F. Giuli, I. Gkialas, L. K. Gladilin, C. Glasman, G. R. Gledhill, M. Glisic, I. Gnesi, Y. Go, M. Goblirsch-Kolb, D. Godin, S. Goldfarb, T. Golling, M. G. D. Gololo, D. Golubkov, J. P. Gombas, A. Gomes, G. Gomes Da Silva, A. J. Gomez Delegido, R. Goncalves Gama, R. Gonçalo, G. Gonella, L. Gonella, A. Gongadze, F. Gonnella, J. L. Gonski, S. González de la Hoz, S. Gonzalez Fernandez, R. Gonzalez Lopez, C. Gonzalez Renteria, R. Gonzalez Suarez, S. Gonzalez-Sevilla, G. R. Gonzalvo Rodriguez, R. Y. González Andana, L. Goossens, N. A. Gorasia, P. A. Gorbounov, B. Gorini, E. Gorini, A. Gorišek, A. T. Goshaw, M. I. Gostkin, C. A. Gottardo, M. Gouighri, V. Goumarre, A. G. Goussiou, N. Govender, C. Goy, I. Grabowska-Bold, K. Graham, E. Gramstad, S. Grancagnolo, M. Grandi, V. Gratchev, P. M. Gravila, F. G. Gravili, H. M. Gray, M. Greco, C. Grefe, I. M. Gregor, P. Grenier, C. Grieco, A. A. Grillo, K. Grimm, S. Grinstein, J.-F. Grivaz, E. Gross, J. Grosse-Knetter, C. Grud, A. Grummer, J. C. Grundy, L. Guan, W. Guan, C. Gubbels, J. G. R. Guerrero Rojas, G. Guerrieri, F. Guescini, R. Gugel, J. A. M. Guhit, A. Guida, T. Guillemin, E. Guilloton, S. Guindon, F. Guo, J. Guo, L. Guo, Y. Guo, R. Gupta, S. Gurbuz, S. S. Gurdasani, G. Gustavino, M. Guth, P. Gutierrez, L. F. Gutierrez Zagazeta, C. Gutschow, C. Guyot, C. Gwenlan, C. B. Gwilliam, E. S. Haaland, A. Haas, M. Habedank, C. Haber, H. K. Hadavand, A. Hadef, S. Hadzic, M. Haleem, J. Haley, J. J. Hall, G. D. Hallewell, L. Halser, K. Hamano, H. Hamdaoui, M. Hamer, G. N. Hamity, J. Han, K. Han, L. Han, L. Han, S. Han, Y. F. Han, K. Hanagaki, M. Hance, D. A. Hangal, M. D. Hank, R. Hankache, J. B. Hansen, J. D. Hansen, P. H. Hansen, K. Hara, D. Harada, T. Harenberg, S. Harkusha, Y. T. Harris, N. M. Harrison, P. F. Harrison, N. M. Hartman, N. M. Hartmann, Y. Hasegawa, A. Hasib, S. Haug, R. Hauser, M. Havranek, C. M. Hawkes, R. J. Hawkings, S. Hayashida, D. Hayden, C. Hayes, R. L. Hayes, C. P. Hays, J. M. Hays, H. S. Hayward, F. He, Y. He, Y. He, M. P. Heath, V. Hedberg, A. L. Heggelund, N. D. Hehir, C. Heidegger, K. K. Heidegger, W. D. Heidorn, J. Heilman, S. Heim, T. Heim, J. G. Heinlein, J. J. Heinrich, L. Heinrich, J. Hejbal, L. Helary, A. Held, S. Hellesund, C. M. Helling, S. Hellman, C. Helsens, R. C. W. Henderson, L. Henkelmann, A. M. Henriques Correia, H. Herde, Y. Hernández Jiménez, H. Herr, M. G. Herrmann, T. Herrmann, G. Herten, R. Hertenberger, L. Hervas, N. P. Hessey, H. Hibi, E. Higón-Rodriguez, S. J. Hillier, I. Hinchliffe, F. Hinterkeuser, M. Hirose, S. Hirose, D. Hirschbuehl, T. G. Hitchings, B. Hiti, J. Hobbs, R. Hobincu, N. Hod, M. C. Hodgkinson, B. H. Hodkinson, A. Hoecker, J. Hofer, D. Hohn, T. Holm, M. Holzbock, L. B. A. H. Hommels, B. P. Honan, J. Hong, T. M. Hong, Y. Hong, J. C. Honig, A. Hönle, B. H. Hooberman, W. H. Hopkins, Y. Horii, S. Hou, A. S. Howard, J. Howarth, J. Hoya, M. Hrabovsky, A. Hrynevich, T. Hryn’ova, P. J. Hsu, S.-C. Hsu, Q. Hu, Y. F. Hu, D. P. Huang, S. Huang, X. Huang, Y. Huang, Y. Huang, Z. Huang, Z. Hubacek, M. Huebner, F. Huegging, T. B. Huffman, M. Huhtinen, S. K. Huiberts, R. Hulsken, N. Huseynov, J. Huston, J. Huth, R. Hyneman, S. Hyrych, G. Iacobucci, G. Iakovidis, I. Ibragimov, L. Iconomidou-Fayard, P. Iengo, R. Iguchi, T. Iizawa, Y. Ikegami, A. Ilg, N. Ilic, H. Imam, T. Ingebretsen Carlson, G. Introzzi, M. Iodice, V. Ippolito, M. Ishino, W. Islam, C. Issever, S. Istin, H. Ito, J. M. Iturbe Ponce, R. Iuppa, A. Ivina, J. M. Izen, V. Izzo, P. Jacka, P. Jackson, R. M. Jacobs, B. P. Jaeger, C. S. Jagfeld, G. Jäkel, K. Jakobs, T. Jakoubek, J. Jamieson, K. W. Janas, G. Jarlskog, A. E. Jaspan, T. Javůrek, M. Javurkova, F. Jeanneau, L. Jeanty, J. Jejelava, P. Jenni, C. E. Jessiman, S. Jézéquel, J. Jia, X. Jia, X. Jia, Z. Jia, Y. Jiang, S. Jiggins, J. Jimenez Pena, S. Jin, A. Jinaru, O. Jinnouchi, H. Jivan, P. Johansson, K. A. Johns, C. A. Johnson, D. M. Jones, E. Jones, P. Jones, R. W. L. Jones, T. J. Jones, J. Jovicevic, X. Ju, J. J. Junggeburth, A. Juste Rozas, S. Kabana, A. Kaczmarska, M. Kado, H. Kagan, M. Kagan, A. Kahn, A. Kahn, C. Kahra, T. Kaji, E. Kajomovitz, N. Kakati, C. W. Kalderon, A. Kamenshchikov, N. J. Kang, Y. Kano, D. Kar, K. Karava, M. J. Kareem, E. Karentzos, I. Karkanias, S. N. Karpov, Z. M. Karpova, V. Kartvelishvili, A. N. Karyukhin, E. Kasimi, C. Kato, J. Katzy, S. Kaur, K. Kawade, K. Kawagoe, T. Kawaguchi, T. Kawamoto, G. Kawamura, E. F. Kay, F. I. Kaya, S. Kazakos, V. F. Kazanin, Y. Ke, J. M. Keaveney, R. Keeler, G. V. Kehris, J. S. Keller, A. S. Kelly, D. Kelsey, J. J. Kempster, J. Kendrick, K. E. Kennedy, O. Kepka, B. P. Kerridge, S. Kersten, B. P. Kerševan, L. Keszeghova, S. Ketabchi Haghighat, M. Khandoga, A. Khanov, A. G. Kharlamov, T. Kharlamova, E. E. Khoda, T. J. Khoo, G. Khoriauli, J. Khubua, Y. A. R. Khwaira, M. Kiehn, A. Kilgallon, D. W. Kim, E. Kim, Y. K. Kim, N. Kimura, A. Kirchhoff, D. Kirchmeier, C. Kirfel, J. Kirk, A. E. Kiryunin, T. Kishimoto, D. P. Kisliuk, C. Kitsaki, O. Kivernyk, M. Klassen, C. Klein, L. Klein, M. H. Klein, M. Klein, U. Klein, P. Klimek, A. Klimentov, F. Klimpel, T. Klingl, T. Klioutchnikova, F. F. Klitzner, P. Kluit, S. Kluth, E. Kneringer, T. M. Knight, A. Knue, D. Kobayashi, R. Kobayashi, M. Kocian, T. Kodama, P. Kodyš, D. M. Koeck, P. T. Koenig, T. Koffas, N. M. Köhler, M. Kolb, I. Koletsou, T. Komarek, K. Köneke, A. X. Y. Kong, T. Kono, N. Konstantinidis, B. Konya, R. Kopeliansky, S. Koperny, K. Korcyl, K. Kordas, G. Koren, A. Korn, S. Korn, I. Korolkov, N. Korotkova, B. Kortman, O. Kortner, S. Kortner, W. H. Kostecka, V. V. Kostyukhin, A. Kotsokechagia, A. Kotwal, A. Koulouris, A. Kourkoumeli-Charalampidi, C. Kourkoumelis, E. Kourlitis, O. Kovanda, R. Kowalewski, W. Kozanecki, A. S. Kozhin, V. A. Kramarenko, G. Kramberger, P. Kramer, M. W. Krasny, A. Krasznahorkay, J. A. Kremer, T. Kresse, J. Kretzschmar, K. Kreul, P. Krieger, F. Krieter, S. Krishnamurthy, A. Krishnan, M. Krivos, K. Krizka, K. Kroeninger, H. Kroha, J. Kroll, J. Kroll, K. S. Krowpman, U. Kruchonak, H. Krüger, N. Krumnack, M. C. Kruse, J. A. Krzysiak, A. Kubota, O. Kuchinskaia, S. Kuday, D. Kuechler, J. T. Kuechler, S. Kuehn, T. Kuhl, V. Kukhtin, Y. Kulchitsky, S. Kuleshov, M. Kumar, N. Kumari, M. Kuna, A. Kupco, T. Kupfer, A. Kupich, O. Kuprash, H. Kurashige, L. L. Kurchaninov, Y. A. Kurochkin, A. Kurova, E. S. Kuwertz, M. Kuze, A. K. Kvam, J. Kvita, T. Kwan, K. W. Kwok, C. Lacasta, F. Lacava, H. Lacker, D. Lacour, N. N. Lad, E. Ladygin, B. Laforge, T. Lagouri, S. Lai, I. K. Lakomiec, N. Lalloue, J. E. Lambert, S. Lammers, W. Lampl, C. Lampoudis, A. N. Lancaster, E. Lançon, U. Landgraf, M. P. J. Landon, V. S. Lang, R. J. Langenberg, A. J. Lankford, F. Lanni, K. Lantzsch, A. Lanza, A. Lapertosa, J. F. Laporte, T. Lari, F. Lasagni Manghi, M. Lassnig, V. Latonova, T. S. Lau, A. Laudrain, A. Laurier, S. D. Lawlor, Z. Lawrence, M. Lazzaroni, B. Le, B. Leban, A. Lebedev, M. LeBlanc, T. LeCompte, F. Ledroit-Guillon, A. C. A. Lee, G. R. Lee, L. Lee, S. C. Lee, S. Lee, L. L. Leeuw, H. P. Lefebvre, M. Lefebvre, C. Leggett, K. Lehmann, G. Lehmann Miotto, W. A. Leight, A. Leisos, M. A. L. Leite, C. E. Leitgeb, R. Leitner, K. J. C. Leney, T. Lenz, S. Leone, C. Leonidopoulos, A. Leopold, C. Leroy, R. Les, C. G. Lester, M. Levchenko, J. Levêque, D. Levin, L. J. Levinson, M. P. Lewicki, D. J. Lewis, B. Li, B. Li, C. Li, C.-Q. Li, H. Li, H. Li, H. Li, J. Li, K. Li, L. Li, M. Li, Q. Y. Li, S. Li, T. Li, X. Li, Z. Li, Z. Li, Z. Li, Z. Li, Z. Liang, M. Liberatore, B. Liberti, K. Lie, J. Lieber Marin, K. Lin, R. A. Linck, R. E. Lindley, J. H. Lindon, A. Linss, E. Lipeles, A. Lipniacka, T. M. Liss, A. Lister, J. D. Little, B. Liu, B. X. Liu, D. Liu, J. B. Liu, J. K. K. Liu, K. Liu, M. Liu, M. Y. Liu, P. Liu, Q. Liu, X. Liu, Y. Liu, Y. Liu, Y. L. Liu, Y. W. Liu, M. Livan, J. Llorente Merino, S. L. Lloyd, E. M. Lobodzinska, P. Loch, S. Loffredo, T. Lohse, K. Lohwasser, M. Lokajicek, J. D. Long, I. Longarini, L. Longo, R. Longo, I. Lopez Paz, A. Lopez Solis, J. Lorenz, N. Lorenzo Martinez, A. M. Lory, A. Lösle, X. Lou, X. Lou, A. Lounis, J. Love, P. A. Love, J. J. Lozano Bahilo, G. Lu, M. Lu, S. Lu, Y. J. Lu, H. J. Lubatti, C. Luci, F. L. Lucio Alves, A. Lucotte, F. Luehring, I. Luise, O. Lukianchuk, O. Lundberg, B. Lund-Jensen, N. A. Luongo, M. S. Lutz, D. Lynn, H. Lyons, R. Lysak, E. Lytken, F. Lyu, V. Lyubushkin, T. Lyubushkina, H. Ma, L. L. Ma, Y. Ma, D. M. Mac Donell, G. Maccarrone, J. C. MacDonald, R. Madar, W. F. Mader, J. Maeda, T. Maeno, M. Maerker, V. Magerl, J. Magro, H. Maguire, D. J. Mahon, C. Maidantchik, A. Maio, K. Maj, O. Majersky, S. Majewski, N. Makovec, V. Maksimovic, B. Malaescu, Pa. Malecki, V. P. Maleev, F. Malek, D. Malito, U. Mallik, C. Malone, S. Maltezos, S. Malyukov, J. Mamuzic, G. Mancini, G. Manco, J. P. Mandalia, I. Mandić, L. Manhaes de Andrade Filho, I. M. Maniatis, M. Manisha, J. Manjarres Ramos, D. C. Mankad, K. H. Mankinen, A. Mann, A. Manousos, B. Mansoulie, S. Manzoni, A. Marantis, G. Marchiori, M. Marcisovsky, L. Marcoccia, C. Marcon, M. Marinescu, M. Marjanovic, Z. Marshall, S. Marti-Garcia, T. A. Martin, V. J. Martin, B. Martin dit Latour, L. Martinelli, M. Martinez, P. Martinez Agullo, V. I. Martinez Outschoorn, P. Martinez Suarez, S. Martin-Haugh, V. S. Martoiu, A. C. Martyniuk, A. Marzin, S. R. Maschek, L. Masetti, T. Mashimo, J. Masik, A. L. Maslennikov, L. Massa, P. Massarotti, P. Mastrandrea, A. Mastroberardino, T. Masubuchi, T. Mathisen, A. Matic, N. Matsuzawa, J. Maurer, B. Maček, D. A. Maximov, R. Mazini, I. Maznas, M. Mazza, S. M. Mazza, C. Mc Ginn, J. P. Mc Gowan, S. P. Mc Kee, T. G. McCarthy, W. P. McCormack, E. F. McDonald, A. E. McDougall, J. A. Mcfayden, G. Mchedlidze, R. P. Mckenzie, T. C. Mclachlan, D. J. Mclaughlin, K. D. McLean, S. J. McMahon, P. C. McNamara, R. A. McPherson, J. E. Mdhluli, S. Meehan, T. Megy, S. Mehlhase, A. Mehta, B. Meirose, D. Melini, B. R. Mellado Garcia, A. H. Melo, F. Meloni, E. D. Mendes Gouveia, A. M. Mendes Jacques Da Costa, H. Y. Meng, L. Meng, S. Menke, M. Mentink, E. Meoni, C. Merlassino, L. Merola, C. Meroni, G. Merz, O. Meshkov, J. K. R. Meshreki, J. Metcalfe, A. S. Mete, C. Meyer, J.-P. Meyer, M. Michetti, R. P. Middleton, L. Mijović, G. Mikenberg, M. Mikestikova, M. Mikuž, H. Mildner, A. Milic, C. D. Milke, D. W. Miller, L. S. Miller, A. Milov, D. A. Milstead, T. Min, A. A. Minaenko, I. A. Minashvili, L. Mince, A. I. Mincer, B. Mindur, M. Mineev, Y. Minegishi, Y. Mino, L. M. Mir, M. Miralles Lopez, M. Mironova, T. Mitani, A. Mitra, V. A. Mitsou, O. Miu, P. S. Miyagawa, Y. Miyazaki, A. Mizukami, J. U. Mjörnmark, T. Mkrtchyan, M. Mlynarikova, T. Moa, S. Mobius, K. Mochizuki, P. Moder, P. Mogg, A. F. Mohammed, S. Mohapatra, G. Mokgatitswane, B. Mondal, S. Mondal, K. Mönig, E. Monnier, L. Monsonis Romero, J. Montejo Berlingen, M. Montella, F. Monticelli, N. Morange, A. L. Moreira De Carvalho, M. Moreno Llácer, C. Moreno Martinez, P. Morettini, S. Morgenstern, M. Morii, M. Morinaga, V. Morisbak, A. K. Morley, F. Morodei, L. Morvaj, P. Moschovakos, B. Moser, M. Mosidze, T. Moskalets, P. Moskvitina, J. Moss, E. J. W. Moyse, S. Muanza, J. Mueller, D. Muenstermann, R. Müller, G. A. Mullier, J. J. Mullin, D. P. Mungo, J. L. Munoz Martinez, D. Munoz Perez, F. J. Munoz Sanchez, M. Murin, W. J. Murray, A. Murrone, J. M. Muse, M. Muškinja, C. Mwewa, A. G. Myagkov, A. J. Myers, A. A. Myers, G. Myers, M. Myska, B. P. Nachman, O. Nackenhorst, A. Nag, K. Nagai, K. Nagano, J. L. Nagle, E. Nagy, A. M. Nairz, Y. Nakahama, K. Nakamura, H. Nanjo, R. Narayan, E. A. Narayanan, I. Naryshkin, M. Naseri, C. Nass, G. Navarro, J. Navarro-Gonzalez, R. Nayak, P. Y. Nechaeva, F. Nechansky, T. J. Neep, A. Negri, M. Negrini, C. Nellist, C. Nelson, K. Nelson, S. Nemecek, M. Nessi, M. S. Neubauer, F. Neuhaus, J. Neundorf, R. Newhouse, P. R. Newman, C. W. Ng, Y. S. Ng, Y. W. Y. Ng, B. Ngair, H. D. N. Nguyen, R. B. Nickerson, R. Nicolaidou, J. Nielsen, M. Niemeyer, N. Nikiforou, V. Nikolaenko, I. Nikolic-Audit, K. Nikolopoulos, P. Nilsson, H. R. Nindhito, A. Nisati, N. Nishu, R. Nisius, J.-E. Nitschke, E. K. Nkadimeng, S. J. Noacco Rosende, T. Nobe, D. L. Noel, Y. Noguchi, T. Nommensen, M. A. Nomura, M. B. Norfolk, R. R. B. Norisam, B. J. Norman, J. Novak, T. Novak, O. Novgorodova, L. Novotny, R. Novotny, L. Nozka, K. Ntekas, E. Nurse, F. G. Oakham, J. Ocariz, A. Ochi, I. Ochoa, S. Oerdek, A. Ogrodnik, A. Oh, C. C. Ohm, H. Oide, R. Oishi, M. L. Ojeda, Y. Okazaki, M. W. O’Keefe, Y. Okumura, A. Olariu, L. F. Oleiro Seabra, S. A. Olivares Pino, D. Oliveira Damazio, D. Oliveira Goncalves, J. L. Oliver, M. J. R. Olsson, A. Olszewski, J. Olszowska, Ö. O. Öncel, D. C. O’Neil, A. P. O’Neill, A. Onofre, P. U. E. Onyisi, M. J. Oreglia, G. E. Orellana, D. Orestano, N. Orlando, R. S. Orr, V. O’Shea, R. Ospanov, G. Otero y Garzon, H. Otono, P. S. Ott, G. J. Ottino, M. Ouchrif, J. Ouellette, F. Ould-Saada, M. Owen, R. E. Owen, K. Y. Oyulmaz, V. E. Ozcan, N. Ozturk, S. Ozturk, J. Pacalt, H. A. Pacey, K. Pachal, A. Pacheco Pages, C. Padilla Aranda, G. Padovano, S. Pagan Griso, G. Palacino, A. Palazzo, S. Palazzo, S. Palestini, M. Palka, J. Pan, T. Pan, D. K. Panchal, C. E. Pandini, J. G. Panduro Vazquez, H. Pang, P. Pani, G. Panizzo, L. Paolozzi, C. Papadatos, S. Parajuli, A. Paramonov, C. Paraskevopoulos, D. Paredes Hernandez, T. H. Park, M. A. Parker, F. Parodi, E. W. Parrish, V. A. Parrish, J. A. Parsons, U. Parzefall, B. Pascual Dias, L. Pascual Dominguez, V. R. Pascuzzi, F. Pasquali, E. Pasqualucci, S. Passaggio, F. Pastore, P. Pasuwan, J. R. Pater, J. Patton, T. Pauly, J. Pearkes, M. Pedersen, R. Pedro, S. V. Peleganchuk, O. Penc, C. Peng, H. Peng, K. E. Penski, M. Penzin, B. S. Peralva, A. P. Pereira Peixoto, L. Pereira Sanchez, D. V. Perepelitsa, E. Perez Codina, M. Perganti, L. Perini, H. Pernegger, S. Perrella, A. Perrevoort, O. Perrin, K. Peters, R. F. Y. Peters, B. A. Petersen, T. C. Petersen, E. Petit, V. Petousis, C. Petridou, A. Petrukhin, M. Pettee, N. E. Pettersson, A. Petukhov, K. Petukhova, A. Peyaud, R. Pezoa, L. Pezzotti, G. Pezzullo, T. Pham, P. W. Phillips, M. W. Phipps, G. Piacquadio, E. Pianori, F. Piazza, R. Piegaia, D. Pietreanu, A. D. Pilkington, M. Pinamonti, J. L. Pinfold, B. C. Pinheiro Pereira, C. Pitman Donaldson, D. A. Pizzi, L. Pizzimento, A. Pizzini, M.-A. Pleier, V. Plesanovs, V. Pleskot, E. Plotnikova, G. Poddar, R. Poettgen, R. Poggi, L. Poggioli, I. Pogrebnyak, D. Pohl, I. Pokharel, S. Polacek, G. Polesello, A. Poley, R. Polifka, A. Polini, C. S. Pollard, Z. B. Pollock, V. Polychronakos, D. Ponomarenko, L. Pontecorvo, S. Popa, G. A. Popeneciu, D. M. Portillo Quintero, S. Pospisil, P. Postolache, K. Potamianos, I. N. Potrap, C. J. Potter, H. Potti, T. Poulsen, J. Poveda, G. Pownall, M. E. Pozo Astigarraga, A. Prades Ibanez, M. M. Prapa, D. Price, M. Primavera, M. A. Principe Martin, M. L. Proffitt, N. Proklova, K. Prokofiev, G. Proto, S. Protopopescu, J. Proudfoot, M. Przybycien, J. E. Puddefoot, D. Pudzha, P. Puzo, D. Pyatiizbyantseva, J. Qian, Y. Qin, T. Qiu, A. Quadt, M. Queitsch-Maitland, G. Rabanal Bolanos, D. Rafanoharana, F. Ragusa, J. L. Rainbolt, J. A. Raine, S. Rajagopalan, E. Ramakoti, K. Ran, V. Raskina, D. F. Rassloff, S. Rave, B. Ravina, I. Ravinovich, M. Raymond, A. L. Read, N. P. Readioff, D. M. Rebuzzi, G. Redlinger, K. Reeves, J. A. Reidelsturz, D. Reikher, A. Reiss, A. Rej, C. Rembser, A. Renardi, M. Renda, M. B. Rendel, A. G. Rennie, S. Resconi, M. Ressegotti, E. D. Resseguie, S. Rettie, B. Reynolds, E. Reynolds, M. Rezaei Estabragh, O. L. Rezanova, P. Reznicek, E. Ricci, R. Richter, S. Richter, E. Richter-Was, M. Ridel, P. Rieck, P. Riedler, M. Rijssenbeek, A. Rimoldi, M. Rimoldi, L. Rinaldi, T. T. Rinn, M. P. Rinnagel, G. Ripellino, I. Riu, P. Rivadeneira, J. C. Rivera Vergara, F. Rizatdinova, E. Rizvi, C. Rizzi, B. A. Roberts, B. R. Roberts, S. H. Robertson, M. Robin, D. Robinson, C. M. Robles Gajardo, M. Robles Manzano, A. Robson, A. Rocchi, C. Roda, S. Rodriguez Bosca, Y. Rodriguez Garcia, A. Rodriguez Rodriguez, A. M. Rodríguez Vera, S. Roe, J. T. Roemer, A. R. Roepe-Gier, J. Roggel, O. Røhne, R. A. Rojas, B. Roland, C. P. A. Roland, J. Roloff, A. Romaniouk, E. Romano, M. Romano, A. C. Romero Hernandez, N. Rompotis, L. Roos, S. Rosati, B. J. Rosser, E. Rossi, E. Rossi, L. P. Rossi, L. Rossini, R. Rosten, M. Rotaru, B. Rottler, D. Rousseau, D. Rousso, G. Rovelli, A. Roy, A. Rozanov, Y. Rozen, X. Ruan, A. Rubio Jimenez, A. J. Ruby, T. A. Ruggeri, F. Rühr, A. Ruiz-Martinez, A. Rummler, Z. Rurikova, N. A. Rusakovich, H. L. Russell, J. P. Rutherfoord, E. M. Rüttinger, K. Rybacki, M. Rybar, E. B. Rye, A. Ryzhov, J. A. Sabater Iglesias, P. Sabatini, L. Sabetta, H. F.-W. Sadrozinski, F. Safai Tehrani, B. Safarzadeh Samani, M. Safdari, S. Saha, M. Sahinsoy, M. Saimpert, M. Saito, T. Saito, D. Salamani, G. Salamanna, A. Salnikov, J. Salt, A. Salvador Salas, D. Salvatore, F. Salvatore, A. Salzburger, D. Sammel, D. Sampsonidis, D. Sampsonidou, J. Sánchez, A. Sanchez Pineda, V. Sanchez Sebastian, H. Sandaker, C. O. Sander, J. A. Sandesara, M. Sandhoff, C. Sandoval, D. P. C. Sankey, A. Sansoni, L. Santi, C. Santoni, H. Santos, S. N. Santpur, A. Santra, K. A. Saoucha, J. G. Saraiva, J. Sardain, O. Sasaki, K. Sato, C. Sauer, F. Sauerburger, E. Sauvan, P. Savard, R. Sawada, C. Sawyer, L. Sawyer, I. Sayago Galvan, C. Sbarra, A. Sbrizzi, T. Scanlon, J. Schaarschmidt, P. Schacht, D. Schaefer, U. Schäfer, A. C. Schaffer, D. Schaile, R. D. Schamberger, E. Schanet, C. Scharf, V. A. Schegelsky, D. Scheirich, F. Schenck, M. Schernau, C. Scheulen, C. Schiavi, Z. M. Schillaci, E. J. Schioppa, M. Schioppa, B. Schlag, K. E. Schleicher, S. Schlenker, K. Schmieden, C. Schmitt, S. Schmitt, L. Schoeffel, A. Schoening, P. G. Scholer, E. Schopf, M. Schott, J. Schovancova, S. Schramm, F. Schroeder, H.-C. Schultz-Coulon, M. Schumacher, B. A. Schumm, Ph. Schune, A. Schwartzman, T. A. Schwarz, Ph. Schwemling, R. Schwienhorst, A. Sciandra, G. Sciolla, F. Scuri, F. Scutti, C. D. Sebastiani, K. Sedlaczek, P. Seema, S. C. Seidel, A. Seiden, B. D. Seidlitz, T. Seiss, C. Seitz, J. M. Seixas, G. Sekhniaidze, S. J. Sekula, L. Selem, N. Semprini-Cesari, S. Sen, D. Sengupta, V. Senthilkumar, L. Serin, L. Serkin, M. Sessa, H. Severini, S. Sevova, F. Sforza, A. Sfyrla, E. Shabalina, R. Shaheen, J. D. Shahinian, N. W. Shaikh, D. Shaked Renous, L. Y. Shan, M. Shapiro, A. Sharma, A. S. Sharma, P. Sharma, S. Sharma, P. B. Shatalov, K. Shaw, S. M. Shaw, Q. Shen, P. Sherwood, L. Shi, C. O. Shimmin, Y. Shimogama, J. D. Shinner, I. P. J. Shipsey, S. Shirabe, M. Shiyakova, J. Shlomi, M. J. Shochet, J. Shojaii, D. R. Shope, S. Shrestha, E. M. Shrif, M. J. Shroff, P. Sicho, A. M. Sickles, E. Sideras Haddad, O. Sidiropoulou, A. Sidoti, F. Siegert, Dj. Sijacki, R. Sikora, F. Sili, J. M. Silva, M. V. Silva Oliveira, S. B. Silverstein, S. Simion, R. Simoniello, E. L. Simpson, N. D. Simpson, S. Simsek, S. Sindhu, P. Sinervo, V. Sinetckii, S. Singh, S. Singh, S. Sinha, S. Sinha, M. Sioli, I. Siral, S. Yu. Sivoklokov, J. Sjölin, A. Skaf, E. Skorda, P. Skubic, M. Slawinska, V. Smakhtin, B. H. Smart, J. Smiesko, S. Yu. Smirnov, Y. Smirnov, L. N. Smirnova, O. Smirnova, A. C. Smith, E. A. Smith, H. A. Smith, J. L. Smith, R. Smith, M. Smizanska, K. Smolek, A. Smykiewicz, A. A. Snesarev, H. L. Snoek, S. Snyder, R. Sobie, A. Soffer, C. A. Solans Sanchez, E. Yu. Soldatov, U. Soldevila, A. A. Solodkov, S. Solomon, A. Soloshenko, K. Solovieva, O. V. Solovyanov, V. Solovyev, P. Sommer, A. Sonay, W. Y. Song, A. Sopczak, A. L. Sopio, F. Sopkova, V. Sothilingam, S. Sottocornola, R. Soualah, Z. Soumaimi, D. South, S. Spagnolo, M. Spalla, F. Spanò, D. Sperlich, G. Spigo, M. Spina, S. Spinali, D. P. Spiteri, M. Spousta, E. J. Staats, A. Stabile, R. Stamen, M. Stamenkovic, A. Stampekis, M. Standke, E. Stanecka, B. Stanislaus, M. M. Stanitzki, M. Stankaityte, B. Stapf, E. A. Starchenko, G. H. Stark, J. Stark, D. M. Starko, P. Staroba, P. Starovoitov, S. Stärz, R. Staszewski, G. Stavropoulos, J. Steentoft, P. Steinberg, A. L. Steinhebel, B. Stelzer, H. J. Stelzer, O. Stelzer-Chilton, H. Stenzel, T. J. Stevenson, G. A. Stewart, M. C. Stockton, G. Stoicea, M. Stolarski, S. Stonjek, A. Straessner, J. Strandberg, S. Strandberg, M. Strauss, T. Strebler, P. Strizenec, R. Ströhmer, D. M. Strom, L. R. Strom, R. Stroynowski, A. Strubig, S. A. Stucci, B. Stugu, J. Stupak, N. A. Styles, D. Su, S. Su, W. Su, X. Su, K. Sugizaki, V. V. Sulin, M. J. Sullivan, D. M. S. Sultan, L. Sultanaliyeva, S. Sultansoy, T. Sumida, S. Sun, S. Sun, O. Sunneborn Gudnadottir, M. R. Sutton, M. Svatos, M. Swiatlowski, T. Swirski, I. Sykora, M. Sykora, T. Sykora, D. Ta, K. Tackmann, A. Taffard, R. Tafirout, J. S. Tafoya Vargas, R. H. M. Taibah, R. Takashima, K. Takeda, E. P. Takeva, Y. Takubo, M. Talby, A. A. Talyshev, K. C. Tam, N. M. Tamir, A. Tanaka, J. Tanaka, R. Tanaka, M. Tanasini, J. Tang, Z. Tao, S. Tapia Araya, S. Tapprogge, A. Tarek Abouelfadl Mohamed, S. Tarem, K. Tariq, G. Tarna, G. F. Tartarelli, P. Tas, M. Tasevsky, E. Tassi, A. C. Tate, G. Tateno, Y. Tayalati, G. N. Taylor, W. Taylor, H. Teagle, A. S. Tee, R. Teixeira De Lima, P. Teixeira-Dias, J. J. Teoh, K. Terashi, J. Terron, S. Terzo, M. Testa, R. J. Teuscher, A. Thaler, N. Themistokleous, T. Theveneaux-Pelzer, O. Thielmann, D. W. Thomas, J. P. Thomas, E. A. Thompson, P. D. Thompson, E. Thomson, E. J. Thorpe, Y. Tian, V. Tikhomirov, Yu. A. Tikhonov, S. Timoshenko, E. X. L. Ting, P. Tipton, S. Tisserant, S. H. Tlou, A. Tnourji, K. Todome, S. Todorova-Nova, S. Todt, M. Togawa, J. Tojo, S. Tokár, K. Tokushuku, R. Tombs, M. Tomoto, L. Tompkins, P. Tornambe, E. Torrence, H. Torres, E. Torró Pastor, M. Toscani, C. Tosciri, D. R. Tovey, A. Traeet, I. S. Trandafir, T. Trefzger, A. Tricoli, I. M. Trigger, S. Trincaz-Duvoid, D. A. Trischuk, B. Trocmé, A. Trofymov, C. Troncon, L. Truong, M. Trzebinski, A. Trzupek, F. Tsai, M. Tsai, A. Tsiamis, P. V. Tsiareshka, S. Tsigaridas, A. Tsirigotis, V. Tsiskaridze, E. G. Tskhadadze, M. Tsopoulou, Y. Tsujikawa, I. I. Tsukerman, V. Tsulaia, S. Tsuno, O. Tsur, D. Tsybychev, Y. Tu, A. Tudorache, V. Tudorache, A. N. Tuna, S. Turchikhin, I. Turk Cakir, R. Turra, T. Turtuvshin, P. M. Tuts, S. Tzamarias, P. Tzanis, E. Tzovara, K. Uchida, F. Ukegawa, P. A. Ulloa Poblete, G. Unal, M. Unal, A. Undrus, G. Unel, K. Uno, J. Urban, P. Urquijo, G. Usai, R. Ushioda, M. Usman, Z. Uysal, V. Vacek, B. Vachon, K. O. H. Vadla, T. Vafeiadis, C. Valderanis, E. Valdes Santurio, M. Valente, S. Valentinetti, A. Valero, A. Vallier, J. A. Valls Ferrer, T. R. Van Daalen, P. Van Gemmeren, S. Van Stroud, I. Van Vulpen, M. Vanadia, W. Vandelli, M. Vandenbroucke, E. R. Vandewall, D. Vannicola, L. Vannoli, R. Vari, E. W. Varnes, C. Varni, T. Varol, D. Varouchas, L. Varriale, K. E. Varvell, M. E. Vasile, L. Vaslin, G. A. Vasquez, F. Vazeille, T. Vazquez Schroeder, J. Veatch, V. Vecchio, M. J. Veen, I. Veliscek, L. M. Veloce, F. Veloso, S. Veneziano, A. Ventura, A. Verbytskyi, M. Verducci, C. Vergis, M. Verissimo De Araujo, W. Verkerke, J. C. Vermeulen, C. Vernieri, P. J. Verschuuren, M. Vessella, M. L. Vesterbacka, M. C. Vetterli, A. Vgenopoulos, N. Viaux Maira, T. Vickey, O. E. Vickey Boeriu, G. H. A. Viehhauser, L. Vigani, M. Villa, M. Villaplana Perez, E. M. Villhauer, E. Vilucchi, M. G. Vincter, G. S. Virdee, A. Vishwakarma, C. Vittori, I. Vivarelli, V. Vladimirov, E. Voevodina, F. Vogel, P. Vokac, J. Von Ahnen, E. Von Toerne, B. Vormwald, V. Vorobel, K. Vorobev, M. Vos, J. H. Vossebeld, M. Vozak, L. Vozdecky, N. Vranjes, M. Vranjes Milosavljevic, M. Vreeswijk, R. Vuillermet, O. Vujinovic, I. Vukotic, S. Wada, C. Wagner, W. Wagner, S. Wahdan, H. Wahlberg, R. Wakasa, M. Wakida, V. M. Walbrecht, J. Walder, R. Walker, W. Walkowiak, A. M. Wang, A. Z. Wang, C. Wang, C. Wang, H. Wang, J. Wang, P. Wang, R.-J. Wang, R. Wang, R. Wang, S. M. Wang, S. Wang, T. Wang, W. T. Wang, W. X. Wang, X. Wang, X. Wang, X. Wang, Y. Wang, Y. Wang, Z. Wang, Z. Wang, A. Warburton, R. J. Ward, N. Warrack, A. T. Watson, M. F. Watson, G. Watts, B. M. Waugh, A. F. Webb, C. Weber, M. S. Weber, S. A. Weber, S. M. Weber, C. Wei, Y. Wei, A. R. Weidberg, J. Weingarten, M. Weirich, C. Weiser, C. J. Wells, T. Wenaus, B. Wendland, T. Wengler, N. S. Wenke, N. Wermes, M. Wessels, K. Whalen, A. M. Wharton, A. S. White, A. White, M. J. White, D. Whiteson, L. Wickremasinghe, W. Wiedenmann, C. Wiel, M. Wielers, N. Wieseotte, C. Wiglesworth, L. A. M. Wiik-Fuchs, D. J. Wilbern, H. G. Wilkens, D. M. Williams, H. H. Williams, S. Williams, S. Willocq, P. J. Windischhofer, F. Winklmeier, B. T. Winter, M. Wittgen, M. Wobisch, A. Wolf, R. Wölker, J. Wollrath, M. W. Wolter, H. Wolters, V. W. S. Wong, A. F. Wongel, S. D. Worm, B. K. Wosiek, K. W. Woźniak, K. Wraight, J. Wu, M. Wu, S. L. Wu, X. Wu, Y. Wu, Z. Wu, J. Wuerzinger, T. R. Wyatt, B. M. Wynne, S. Xella, L. Xia, M. Xia, J. Xiang, X. Xiao, M. Xie, X. Xie, J. Xiong, I. Xiotidis, D. Xu, H. Xu, L. Xu, R. Xu, T. Xu, W. Xu, Y. Xu, Z. Xu, Z. Xu, B. Yabsley, S. Yacoob, N. Yamaguchi, Y. Yamaguchi, H. Yamauchi, T. Yamazaki, Y. Yamazaki, J. Yan, S. Yan, Z. Yan, H. J. Yang, H. T. Yang, S. Yang, T. Yang, X. Yang, X. Yang, Y. Yang, Z. Yang, W.-M. Yao, Y. C. Yap, H. Ye, J. Ye, S. Ye, X. Ye, Y. Yeh, I. Yeletskikh, M. R. Yexley, P. Yin, K. Yorita, C. J. S. Young, C. Young, M. Yuan, R. Yuan, L. Yue, X. Yue, M. Zaazoua, B. Zabinski, E. Zaid, T. Zakareishvili, N. Zakharchuk, S. Zambito, J. Zang, D. Zanzi, O. Zaplatilek, S. V. Zeißner, C. Zeitnitz, J. C. Zeng, D. T. Zenger, O. Zenin, T. Ženiš, S. Zenz, S. Zerradi, D. Zerwas, B. Zhang, D. F. Zhang, G. Zhang, J. Zhang, K. Zhang, L. Zhang, R. Zhang, S. Zhang, T. Zhang, X. Zhang, X. Zhang, Z. Zhang, Z. Zhang, H. Zhao, P. Zhao, T. Zhao, Y. Zhao, Z. Zhao, A. Zhemchugov, Z. Zheng, D. Zhong, B. Zhou, C. Zhou, H. Zhou, N. Zhou, Y. Zhou, C. G. Zhu, C. Zhu, H. L. Zhu, H. Zhu, J. Zhu, Y. Zhu, Y. Zhu, X. Zhuang, K. Zhukov, V. Zhulanov, N. I. Zimine, J. Zinsser, M. Ziolkowski, L. Živković, A. Zoccoli, K. Zoch, T. G. Zorbas, O. Zormpa, W. Zou, L. Zwalinski

**Affiliations:** 1 CPPM, Aix-Marseille Université, CNRS/IN2P3, Marseille, France; 2 Homer L. Dodge Department of Physics and Astronomy, University of Oklahoma, Norman, OK United States of America; 3 Department of Physics, University of Massachusetts, Amherst, MA United States of America; 4 II. Physikalisches Institut, Georg-August-Universität Göttingen, Göttingen, Germany; 5 Physics Department, Brookhaven National Laboratory, Upton, NY United States of America; 6 Faculté des sciences, Université Mohammed V, Rabat, Morocco; 7 Physics Department, An-Najah National University, Nablus, Palestine; 8 Raymond and Beverly Sackler School of Physics and Astronomy, Tel Aviv University, Tel Aviv, Israel; 9 Department of Physics, Technion, Israel Institute of Technology, Haifa, Israel; 10 Department of Physics, New York University, New York, NY United States of America; 11 Departamento de Física, Pontificia Universidad Católica de Chile, Santiago, Chile; 12 INFN Gruppo Collegato di Udine, Sezione di Trieste, Udine, Italy; 13 ICTP, Trieste, Italy; 14 Institut für Physik, Universität Mainz, Mainz, Germany; 15 LAPP, Univ. Savoie Mont Blanc, CNRS/IN2P3, Annecy, France; 16 AGH University of Krakow, Faculty of Physics and Applied Computer Science, Krakow, Poland; 17 Department of Physics, University of Toronto, Toronto, ON Canada; 18 Department of Physics, Brandeis University, Waltham, MA United States of America; 19 Department of Physics, Northern Illinois University, DeKalb, IL United States of America; 20 Department of Physics, Istanbul University, Istanbul, Turkey; 21 Département de Physique Nucléaire et Corpusculaire, Université de Genève, Genève, Switzerland; 22 Particle Physics Department, Rutherford Appleton Laboratory, Didcot, United Kingdom; 23 Santa Cruz Institute for Particle Physics, University of California Santa Cruz, Santa Cruz, CA United States of America; 24 CERN, Geneva, Switzerland; 25 Institut de Física d’Altes Energies (IFAE), Barcelona Institute of Science and Technology, Barcelona, Spain; 26 INFN Sezione di Pavia, Pavia, Italy; 27 Dipartimento di Fisica, Università di Pavia, Pavia, Italy; 28 Department of Physics, Alexandru Ioan Cuza University of Iasi, Iasi, Romania; 29 Departamento de Física Teórica y del Cosmos, Universidad de Granada, Granada, Spain; 30 Affiliated with an international laboratory covered by a cooperation agreement with CERN, Geneva, Switzerland; 31 Department of Physics, McGill University, Montreal, QC Canada; 32 Department of Physics, Royal Holloway University of London, Egham, United Kingdom; 33 Deutsches Elektronen-Synchrotron DESY, Hamburg and Zeuthen, Germany; 34 INFN Sezione di Roma Tor Vergata, Rome, Italy; 35 Dipartimento di Fisica, Università di Roma Tor Vergata, Roma, Italy; 36 Department of Particle Physics and Astrophysics, Weizmann Institute of Science, Rehovot, Israel; 37 Fysiska institutionen, Lunds universitet, Lund, Sweden; 38 Affiliated with an institute covered by a cooperation agreement with CERN, Geneva, Switzerland; 39 Nevis Laboratory, Columbia University, Irvington, NY United States of America; 40 INFN Sezione di Bologna, Bologna, Italy; 41 Department of Physics and Astronomy, University of Victoria, Victoria, BC Canada; 42 INFN e Laboratori Nazionali di Frascati, Frascati, Italy; 43 Instituto de Física La Plata, Universidad Nacional de La Plata and CONICET, La Plata, Argentina; 44 SUPA - School of Physics and Astronomy, University of Edinburgh, Edinburgh, United Kingdom; 45 Horia Hulubei National Institute of Physics and Nuclear Engineering, Bucharest, Romania; 46 Physics Department, National Technical University of Athens, Zografou, Greece; 47 Nikhef National Institute for Subatomic Physics and University of Amsterdam, Amsterdam, Netherlands; 48 Czech Technical University in Prague, Prague, Czech Republic; 49 Institute of Physics, Academia Sinica, Taipei, Taiwan; 50 INFN Sezione di Milano, Milan, Italy; 51 Department of Physics and Astronomy, University of Sussex, Brighton, United Kingdom; 52 School of Physics and Astronomy, University of Birmingham, Birmingham, United Kingdom; 53 INFN Sezione di Napoli, Napoli, Italy; 54 Dipartimento di Fisica, Università di Napoli, Napoli, Italy; 55 Department of Physics, University of Washington, Seattle, WA United States of America; 56 Departamento de Física Teorica C-15 and CIAFF, Universidad Autónoma de Madrid, Madrid, Spain; 57 Universidade Federal do Rio De Janeiro COPPE/EE/IF, Rio de Janeiro, Brazil; 58 Fakultät für Physik, Ludwig-Maximilians-Universität München, München, Germany; 59 Department of Physics, University of Michigan, Ann Arbor, MI United States of America; 60 Laboratório de Instrumentação e Física Experimental de Partículas - LIP, Lisboa, Portugal; 61 Instituto de Física Corpuscular (IFIC), Centro Mixto Universidad de Valencia - CSIC, Valencia, Spain; 62 Department of Physics, University of Oslo, Oslo, Norway; 63 Department of Physics and Astronomy, University of Sheffield, Sheffield, United Kingdom; 64 IRFU, CEA, Université Paris-Saclay, Gif-sur-Yvette, France; 65 Department of Physics, University of Texas at Austin, Austin, TX United States of America; 66 Albert Einstein Center for Fundamental Physics and Laboratory for High Energy Physics, University of Bern, Bern, Switzerland; 67 Department of Physics, Stockholm University, Stockholm, Sweden; 68 Oskar Klein Centre, Stockholm, Sweden; 69 Dipartimento di Fisica, Università di Milano, Milano, Italy; 70 Physics Department, National and Kapodistrian University of Athens, Athens, Greece; 71 INFN Sezione di Pisa, Pisa, Italy; 72 Department of Physics, Oklahoma State University, Stillwater, OK United States of America; 73 Physics Division, Lawrence Berkeley National Laboratory, Berkeley, CA United States of America; 74 Institute for Nuclear Research and Nuclear Energy (INRNE) of the Bulgarian Academy of Sciences, Sofia, Bulgaria; 75 INFN Sezione di Roma, Roma, Italy; 76 KEK, High Energy Accelerator Research Organization, Tsukuba, Japan; 77 Institut für Physik, Humboldt Universität zu Berlin, Berlin, Germany; 78 Departamento de Engenharia Elétrica, Universidade Federal de Juiz de Fora (UFJF), Juiz de Fora, Brazil; 79 Department of Physics, Duke University, Durham, NC United States of America; 80 Oliver Lodge Laboratory, University of Liverpool, Liverpool, United Kingdom; 81 Group of Particle Physics, University of Montreal, Montreal, QC Canada; 82 Physikalisches Institut, Albert-Ludwigs-Universität Freiburg, Freiburg, Germany; 83 Dipartimento di Fisica, Sapienza Università di Roma, Roma, Italy; 84 Graduate School of Science and Kobayashi-Maskawa Institute, Nagoya University, Nagoya, Japan; 85 Ochanomizu University, Otsuka, Bunkyo-ku, Tokyo, Japan; 86 International Center for Elementary Particle Physics and Department of Physics, University of Tokyo, Tokyo, Japan; 87 Laboratory for Particle Physics and Cosmology, Harvard University, Cambridge, MA United States of America; 88 Department of Physics and Astronomy, University of Uppsala, Uppsala, Sweden; 89 LPMR, Faculté des Sciences, Université Mohamed Premier, Oujda, Morocco; 90 Faculty of Mathematics, Physics and Informatics, Comenius University, Bratislava, Slovak Republic; 91 Department of Physics, University of Cape Town, Cape Town, South Africa; 92 L2IT, Université de Toulouse, CNRS/IN2P3, UPS, Toulouse, France; 93 Department of Physics, University of Illinois, Urbana, IL United States of America; 94 Institute of Frontier and Interdisciplinary Science and Key Laboratory of Particle Physics and Particle Irradiation (MOE), Shandong University, Qingdao, China; 95 Fakultät für Mathematik und Naturwissenschaften, Fachgruppe Physik, Bergische Universität Wuppertal, Wuppertal, Germany; 96 Department of Physics, Nanjing University, Nanjing, China; 97 Department of Physics, Aristotle University of Thessaloniki, Thessaloniki, Greece; 98 Department of Physics, Carleton University, Ottawa, ON Canada; 99 Department of Physics, Yale University, New Haven, CT United States of America; 100 Institute of Physics, University of Belgrade, Belgrade, Serbia; 101 Department of Physics, University of Texas at Arlington, Arlington, TX United States of America; 102 School of Physics, University of Sydney, Sydney, Australia; 103 Charles University, Faculty of Mathematics and Physics, Prague, Czech Republic; 104 Kirchhoff-Institut für Physik, Ruprecht-Karls-Universität Heidelberg, Heidelberg, Germany; 105 Cavendish Laboratory, University of Cambridge, Cambridge, United Kingdom; 106 Institute of Nuclear Physics Polish Academy of Sciences, Krakow, Poland; 107 Department of Physics and Astronomy, University of Pittsburgh, Pittsburgh, PA United States of America; 108 Physikalisches Institut, Universität Bonn, Bonn, Germany; 109 School of Physics, University of Melbourne, Victoria, Australia; 110 Dipartimento di Fisica, Università di Genova, Genova, Italy; 111 INFN Sezione di Genova, Genova, Italy; 112 Department of Physics and Astronomy, University College London, London, United Kingdom; 113 Max-Planck-Institut für Physik (Werner-Heisenberg-Institut), München, Germany; 114 Institute for Fundamental Science, University of Oregon, Eugene, OR United States of America; 115 SLAC National Accelerator Laboratory, Stanford, CA United States of America; 116 Palacký University, Joint Laboratory of Optics, Olomouc, Czech Republic; 117 School of Physics and Astronomy, University of Manchester, Manchester, United Kingdom; 118 Department of Modern Physics and State Key Laboratory of Particle Detection and Electronics, University of Science and Technology of China, Hefei, China; 119 Department of Physics, Oxford University, Oxford, United Kingdom; 120 Institute of High Energy Physics, Chinese Academy of Sciences, Beijing, China; 121 Physics Department, Tsinghua University, Beijing, China; 122 University of Chinese Academy of Science (UCAS), Beijing, China; 123 Physics Department, Lancaster University, Lancaster, United Kingdom; 124 Fakultät Physik, Technische Universität Dortmund, Dortmund, Germany; 125 INFN Sezione di Roma Tre, Roma, Italy; 126 Dipartimento di Matematica e Fisica, Università Roma Tre, Roma, Italy; 127 IJCLab, Université Paris-Saclay, CNRS/IN2P3, 91405 Orsay, France; 128 SUPA - School of Physics and Astronomy, University of Glasgow, Glasgow, United Kingdom; 129 Department Physik, Universität Siegen, Siegen, Germany; 130 Department of Physics, Bogazici University, Istanbul, Turkey; 131 LPNHE, Sorbonne Université, Université Paris Cité, CNRS/IN2P3, Paris, France; 132 Department of Physics and Astronomy, Tufts University, Medford, MA United States of America; 133 Department of Physics, University of Warwick, Coventry, United Kingdom; 134 Istinye University, Sariyer, Istanbul, Turkey; 135 Departments of Physics and Astronomy, Stony Brook University, Stony Brook, NY United States of America; 136 Rio de Janeiro State University, Rio de Janeiro, Brazil; 137 Technical University of Munich, Munich, Germany; 138 United Arab Emirates University, Al Ain, United Arab Emirates; 139 Faculté des Sciences Ain Chock, Réseau Universitaire de Physique des Hautes Energies - Université Hassan II, Casablanca, Morocco; 140 Department of Physics, University of Arizona, Tucson, AZ United States of America; 141 APC, Université Paris Cité, CNRS/IN2P3, Paris, France; 142 Institut für Kern- und Teilchenphysik, Technische Universität Dresden, Dresden, Germany; 143 School of Physics and Astronomy, Queen Mary University of London, London, United Kingdom; 144 Department of Mechanical Engineering Science, University of Johannesburg, Johannesburg, South Africa; 145 Fakultät für Physik und Astronomie, Julius-Maximilians-Universität Würzburg, Würzburg, Germany; 146 High Energy Physics Division, Argonne National Laboratory, Argonne, IL United States of America; 147 Department of Physics Engineering, Gaziantep University, Gaziantep, Türkiye; 148 Dipartimento di Fisica e Astronomia A. Righi, Università di Bologna, Bologna, Italy; 149 Department of Physics, University of Wisconsin, Madison, WI United States of America; 150 LPC, Université Clermont Auvergne, CNRS/IN2P3, Clermont-Ferrand, France; 151 Ohio State University, Columbus, OH United States of America; 152 School of Physics and Astronomy, Shanghai Jiao Tong University, Key Laboratory for Particle Astrophysics and Cosmology (MOE), SKLPPC, Shanghai, China; 153 Tsung-Dao Lee Institute, Shanghai, China; 154 Departamento de Física, Universidad Técnica Federico Santa María, Valparaíso, Chile; 155 Department of Subnuclear Physics, Institute of Experimental Physics of the Slovak Academy of Sciences, Kosice, Slovak Republic; 156 Department for Physics and Technology, University of Bergen, Bergen, Norway; 157 Department of Physics, Simon Fraser University, Burnaby, BC Canada; 158 Department of Physics, Boston University, Boston, MA United States of America; 159 Department of Physics and Astronomy, Michigan State University, East Lansing, MI United States of America; 160 II. Physikalisches Institut, Justus-Liebig-Universität Giessen, Giessen, Germany; 161 Department of Physics, Ankara University, Ankara, Turkey; 162 Department of Physics, Indiana University, Bloomington, IN United States of America; 163 Dipartimento di Fisica E. Fermi, Università di Pisa, Pisa, Italy; 164 Niels Bohr Institute, University of Copenhagen, Copenhagen, Denmark; 165 University of Iowa, Iowa City, IA United States of America; 166 Dipartimento di Fisica, Università della Calabria, Rende, Italy; 167 INFN Gruppo Collegato di Cosenza, Laboratori Nazionali di Frascati, Frascati, Italy; 168 TRIUMF, Vancouver, BC Canada; 169 Institute for Mathematics, Astrophysics and Particle Physics, Radboud University/Nikhef, Nijmegen, Netherlands; 170 School of Physics, University of the Witwatersrand, Johannesburg, South Africa; 171 Departamento de Física, Universidade do Minho, Braga, Portugal; 172 INFN Sezione di Lecce, Lecce, Italy; 173 Dipartimento di Matematica e Fisica, Università del Salento, Lecce, Italy; 174 High Energy Physics Institute, Tbilisi State University, Tbilisi, Georgia; 175 Department of Physics, Stanford University, Stanford CA, United States of America; 176 Department of Physics, Chinese University of Hong Kong, Shatin, N.T., Hong Kong, China; 177 Department of Physics, National Tsing Hua University, Hsinchu, Taiwan; 178 Institute of Physics of the Czech Academy of Sciences, Prague, Czech Republic; 179 Department of Experimental Particle Physics, Jožef Stefan Institute and Department of Physics, University of Ljubljana, Ljubljana, Slovenia; 180 Dipartimento Politecnico di Ingegneria e Architettura, Università di Udine, Udine, Italy; 181 LPSC, Université Grenoble Alpes, CNRS/IN2P3, Grenoble INP, Grenoble, France; 182 Instituto Superior Técnico, Universidade de Lisboa, Lisboa, Portugal; 183 Department of Physics, University of British Columbia, Vancouver, BC Canada; 184 INFN-TIFPA, Povo, Italy; 185 Università degli Studi di Trento, Trento, Italy; 186 Department of Physics and Astronomy, University of California Irvine, Irvine, CA United States of America; 187 Physikalisches Institut, Ruprecht-Karls-Universität Heidelberg, Heidelberg, Germany; 188 Department of Physics, University of Pennsylvania, Philadelphia, PA United States of America; 189 Universidad de Buenos Aires, Facultad de Ciencias Exactas y Naturales, Departamento de Física, y CONICET, Instituto de Física de Buenos Aires (IFIBA), Buenos Aires, Argentina; 190 Department of Physics and Astronomy, York University, Toronto, ON Canada; 191 Physics Department, Southern Methodist University, Dallas, TX United States of America; 192 Millennium Institute for Subatomic physics at high energy frontier (SAPHIR), Santiago, Chile; 193 Instituto de Física, Universidade de São Paulo, São Paulo, Brazil; 194 Faculté des Sciences, Université Ibn-Tofail, Kénitra, Morocco; 196 National Centre for Scientific Research “Demokritos”, Agia Paraskevi, Greece; 197 Physics Department, University of Texas at Dallas, Richardson, TX United States of America; 198 Department of Physics, University of Adelaide, Adelaide, Australia; 199 Departamento de Física, Universidade de Coimbra, Coimbra, Portugal; 200 National Institute of Physics, University of the Philippines, Diliman (Philippines), Diliman, Philippines; 203 Enrico Fermi Institute, University of Chicago, Chicago, IL United States of America; 204 Marian Smoluchowski Institute of Physics, Jagiellonian University, Krakow, Poland; 205 Department of Physics, University of Alberta, Edmonton, AB Canada; 206 Departamento de Física, Faculdade de Ciências, Universidade de Lisboa, Lisboa, Portugal; 207 West University in Timisoara, Timisoara, Romania; 209 California State University, Long Beach, CA United States of America; 210 Department of Physics and Astronomy, University of New Mexico, Albuquerque, NM United States of America; 211 Division of Physics and Tomonaga Center for the History of the Universe, Faculty of Pure and Applied Sciences, University of Tsukuba, Tsukuba, Japan; 212 Department of Physics, Shinshu University, Nagano, Japan; 213 Department of Physics, Tokyo Institute of Technology, Tokyo, Japan; 214 Department of Physics and Astronomy, Iowa State University, Ames IA, United States of America; 215 Graduate School of Science, Kobe University, Kobe, Japan; 216 Graduate School of Science, Osaka University, Osaka, Japan; 217 University Politehnica Bucharest, Bucharest, Romania; 218 Department of Physics, University of Hong Kong, Hong Kong, China; 219 Institute of Physics, Azerbaijan Academy of Sciences, Baku, Azerbaijan; 220 Waseda University, Tokyo, Japan; 221 E. Andronikashvili Institute of Physics, Iv. Javakhishvili Tbilisi State University, Tbilisi, Georgia; 222 Instituto de Alta Investigación, Universidad de Tarapacá, Arica, Chile; 223 Research Center for Advanced Particle Physics and Department of Physics, Kyushu University, Fukuoka, Japan; 224 Universität Innsbruck, Department of Astro and Particle Physics, Innsbruck, Austria; 225 Faculty of Science, Kyoto University, Kyoto, Japan; 226 Universidad Andres Bello, Department of Physics, Santiago, Chile; 227 Department of Physics, Royal Institute of Technology, Stockholm, Sweden; 228 Department of Physics and Institute for Advanced Study, Hong Kong University of Science and Technology, Clear Water Bay, Kowloon, Hong Kong China; 229 Centro de Física Nuclear da Universidade de Lisboa, Lisboa, Portugal; 230 Facultad de Ciencias y Centro de Investigaciónes, Universidad Antonio Nariño, Bogotá, Colombia; 231 Transilvania University of Brasov, Brasov, Romania; 232 National Institute for Research and Development of Isotopic and Molecular Technologies, Physics Department, Cluj-Napoca, Romania; 233 Departamento de Física, Universidad Nacional de Colombia, Bogotá, Colombia; 234 Louisiana Tech University, Ruston, LA United States of America; 235 University of Sharjah, Sharjah, United Arab Emirates; 236 Division of Physics, TOBB University of Economics and Technology, Ankara, Turkey; 237 Kyoto University of Education, Kyoto, Japan; 238 Instituto de Investigación Multidisciplinario en Ciencia y Tecnología, y Departamento de Física, Universidad de La Serena, La Serena, Chile; 239 Affiliated with an institute covered by a cooperation agreement with CERN, Geneva, Switzerland; 240 Borough of Manhattan Community College, City University of New York, New York, NY United States of America; 241 Bruno Kessler Foundation, Trento, Italy; 242 Present Address: Center for High Energy Physics, Peking University, Beijing, China; 243 Centro Studi e Ricerche Enrico Fermi, Roma, Italy; 244 CERN, Geneva, Switzerland; 245 Département de Physique Nucléaire et Corpusculaire, Université de Genève, Genève, Switzerland; 246 Departament de Fisica de la Universitat Autonoma de Barcelona, Barcelona, Spain; 247 Department of Financial and Management Engineering, University of the Aegean, Chios, Greece; 248 Department of Physics and Astronomy, Michigan State University, East Lansing MI, United States of America; 249 Department of Physics and Astronomy, University of Louisville, Louisville, KY United States of America; 250 Department of Physics, Ben Gurion University of the Negev, Beer Sheva, Israel; 251 Department of Physics, California State University, East Bay, United States of America; 252 Department of Physics, California State University, Sacramento, United States of America; 253 Department of Physics, King’s College London, London, United Kingdom; 254 Present Address: Department of Physics, University of Fribourg, Fribourg, Switzerland; 255 Department of Physics, University of Thessaly, Thessaly, Greece; 256 Department of Physics, Westmont College, Santa Barbara, United States of America; 257 Hellenic Open University, Patras, Greece; 258 Present Address: Institucio Catalana de Recerca i Estudis Avancats, ICREA, Barcelona, Spain; 259 Institut für Experimentalphysik, Universität Hamburg, Hamburg, Germany; 260 Institute of Particle Physics (IPP), Ottawa, Canada; 261 Present Address: Institute of Physics, Azerbaijan Academy of Sciences, Baku, Azerbaijan; 262 Institute of Theoretical Physics, Ilia State University, Tbilisi, Georgia; 263 Lawrence Livermore National Laboratory, Livermore, United States of America; 264 Present Address: The City College of New York, New York, NY United States of America; 265 The Collaborative Innovation Center of Quantum Matter (CICQM), Beijing, China; 266 TRIUMF, Vancouver, BC Canada; 267 Università di Napoli Parthenope, Napoli, Italy; 268 University of Chinese Academy of Sciences (UCAS), Beijing, China; 269 Present Address: University of Colorado Boulder, Department of Physics, Colorado, United States of America; 270 Yeditepe University, Physics Department, Istanbul, Turkey

**Keywords:** Particle physics, Experimental particle physics

## Abstract

The standard model of particle physics^1–4^ describes the known fundamental particles and forces that make up our Universe, with the exception of gravity. One of the central features of the standard model is a field that permeates all of space and interacts with fundamental particles^5–9^. The quantum excitation of this field, known as the Higgs field, manifests itself as the Higgs boson, the only fundamental particle with no spin. In 2012, a particle with properties consistent with the Higgs boson of the standard model was observed by the ATLAS and CMS experiments at the Large Hadron Collider at CERN^10,11^. Since then, more than 30 times as many Higgs bosons have been recorded by the ATLAS experiment, enabling much more precise measurements and new tests of the theory. Here, on the basis of this larger dataset, we combine an unprecedented number of production and decay processes of the Higgs boson to scrutinize its interactions with elementary particles. Interactions with gluons, photons, and *W* and *Z* bosons—the carriers of the strong, electromagnetic and weak forces—are studied in detail. Interactions with three third-generation matter particles (bottom (*b*) and top (*t*) quarks, and tau leptons (*τ*)) are well measured and indications of interactions with a second-generation particle (muons, *μ*) are emerging. These tests reveal that the Higgs boson discovered ten years ago is remarkably consistent with the predictions of the theory and provide stringent constraints on many models of new phenomena beyond the standard model.

## Abstract (web summary)

Ten years after the discovery of the Higgs boson, the ATLAS  experiment at CERN probes its kinematic properties with a significantly larger dataset from 2015–2018 and provides further insights on its interaction with other known particles.

## Main

The standard model of particle physics has been tested by many experiments since its formulation^1–4^ and, after accounting for the neutrino masses, no discrepancies between experimental observations and its predictions have been established so far. A central feature of the standard model is the existence of a spinless quantum field that permeates the Universe and gives mass to massive elementary particles. Testing the existence and properties of this field and its associated particle, the Higgs boson, has been one of the main goals of particle physics for several decades. In the standard model, the strength of the interaction, or ‘coupling’, between the Higgs boson and a given particle is fully defined by the particle’s mass and type. There is no direct coupling to the massless standard model force mediators, the photons and gluons, whereas there are three types of couplings to massive particles in the theory. The first is the ‘gauge’ coupling of the Higgs boson to the mediators of the weak force, the *W* and *Z* vector bosons. Demonstrating the existence of gauge couplings is an essential test of the spontaneous electroweak symmetry-breaking mechanism^5–9^. The second type of coupling involves another fundamental interaction, the Yukawa interaction, between the Higgs boson and matter particles, or fermions. The third type of coupling is the ‘self-coupling’ of the Higgs boson to itself. A central prediction of the theory is that the couplings scale with the particle masses and they are all precisely predicted once all the particle masses are known. The experimental determination of the couplings of the Higgs boson to each individual particle therefore provides important and independent tests of the standard model. It also provides stringent constraints on theories beyond the standard model, which generally predict different patterns of coupling values.

In 2012, the ATLAS^12^ and CMS^13^ experiments at the Large Hadron Collider (LHC)^14^ at CERN announced the discovery of a new particle with properties consistent with those predicted for the Higgs boson of the standard model^10,11^. More precise measurements that used all of the proton–proton collision data taken during the first data-taking period from 2011 to 2012 at the LHC (Run 1) showed evidence that, in contrast to all other known fundamental particles, the properties of the discovered particle were consistent with the hypothesis that it has no spin^15,16^. Alternate spin-1 and spin-2 hypotheses were also tested and were excluded at a high level of confidence. Investigations of the charge conjugation and parity (CP) properties of the new particle were also performed, demonstrating consistency with the CP-even quantum state predicted by the standard model, while still allowing for small admixtures of non-standard model CP-even or CP-odd states^15,16^. Limits on the particle’s lifetime were obtained through indirect measurements of its natural width^15–19^. In addition, more precise measurements of the new particle’s interactions with other elementary particles were achieved^20^. The results of all these investigations demonstrated that its properties were compatible with those of the standard model Higgs boson. However, the statistical uncertainties associated with these early measurements allowed considerable room for possible interpretations of the data in terms of new phenomena beyond the standard model and left many predictions of the standard model untested.

The characterization of the Higgs boson continued during the Run 2 data-taking period between 2015 and 2018. About 9 million Higgs bosons are predicted to have been produced in the ATLAS detector during this period, of which only about 0.3% are experimentally accessible. This is 30 times more events than at the time of its discovery, owing to the higher rate of collisions and the increase of the collision energy from 8 teraelectronvolts (TeV) to 13 TeV, which raises the production rate. In this Article, the full Run 2 dataset, corresponding to an integrated luminosity of 139 inverse femtobarns (fb^−1^), is used for the measurements of Higgs boson production and decay rates, which are used to study the couplings between the Higgs boson and the particles involved. This improves on the previous measurements obtained with partial Run 2 datasets^21,22^. The corresponding predictions depend on the value of the Higgs boson mass, which has now been measured by the ATLAS and CMS experiments^23–25^ with an uncertainty of approximately 0.1%. The predictions employed in this article use the combined central value of 125.09 GeV^23^.

**Fig. 1:**
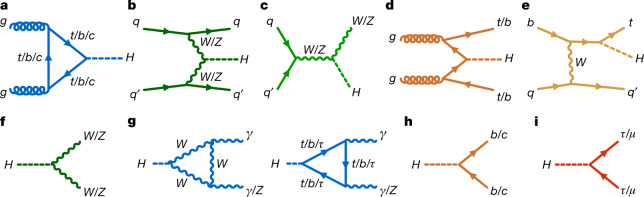
Examples of Feynman diagrams for Higgs boson production and decay. **a**–**e**, The Higgs boson is produced via gluon–gluon fusion (**a**), vector boson fusion (VBF; **b**) and associated production with vector bosons (**c**), top or *b* quark pairs (**d**), or a single top quark (**e**). **f**–**i**, The Higgs boson decays into a pair of vector bosons (**f**), a pair of photons or a *Z* boson and a photon (**g**), a pair of quarks (**h**), and a pair of charged leptons (**i**). Loop-induced Higgs boson interactions with gluons or photons are shown in blue, and processes involving couplings to *W* or *Z* bosons in green, to quarks in orange, and to leptons in red. Two different shades of green (orange) are used to separate the VBF and *VH* ($t\bar{t}H$ and *tH*) production processes.

The dominant production process at the LHC, which accounts for about 87% of Higgs boson production, is the heavy-quark loop-mediated gluon–gluon fusion process (ggF). The second most copious process is vector boson fusion (VBF), in which two weak bosons, either *Z* or *W* bosons, fuse to produce a Higgs boson (7%). Next in rate is production of a Higgs boson in association with a weak (*V* = *W*, *Z*) boson (4%). Production of a Higgs boson in association with a pair of top quarks $(t\bar{t}H)$ or bottom quarks $(b\bar{b}H)$ each account for about 1% of the total rate. The contribution of other $q\bar{q}H$ processes is much smaller and experimentally not accessible. Only about 0.05% of Higgs bosons are produced in association with a single top quark (*tH*). Representative Feynman diagrams of these processes are shown in Fig. 1a–e. After it is produced, the Higgs boson is predicted to decay almost instantly, with a lifetime of 1.6 × 10^−22^ seconds. More than 90% of these decays are via eight decay modes (Fig. 1f–i): decays into gauge boson pairs, that is, *W* bosons with a probability, or branching fraction, of 22%, *Z* bosons 3%, photons (*γ*) 0.2%, *Z* boson and photon 0.2%, as well as decays into fermion pairs, that is, *b* quarks 58%, *c* quarks 3%, *τ* leptons 6%, and muons (*μ*) 0.02%. There may also be decays of the Higgs boson into invisible particles, above the standard model prediction of 0.1%, which are also searched for. Such decays are possible in theories beyond the standard model, postulating, for example, the existence of dark matter particles that do not interact with the detector.

In this Article, the mutually exclusive measurements of Higgs boson production and decays probing all processes listed above are combined, taking into account the correlations among their uncertainties. In a single measurement, different couplings generally contribute in the production and decay. The combination of all measurements is therefore necessary to constrain these couplings individually. This enables key tests of the Higgs sector of the standard model to be performed, including the determination of the coupling strengths of the Higgs boson to various fundamental particles and a comprehensive study of the kinematic properties of Higgs boson production. The latter could reveal new phenomena beyond the standard model that are not observable through measurements of the coupling strengths.

## The ATLAS detector at the LHC

The ATLAS experiment^12^ at the LHC is a multipurpose particle detector with a forward–backward symmetric, cylindrical geometry and a near 4π coverage in solid angle. The detector records digitized signals produced by the products of LHC’s proton bunch collisions, hereafter termed collision ‘events’. It is designed to identify a wide variety of particles and measure their momenta and energies. These particles include electrons, muons, *τ* leptons and photons, as well as gluons and quarks, which produce collimated jets of particles in the detector. Because the jets from *b* quarks and *c* quarks contain hadrons with relatively long lifetimes, they can be identified by observing a decay vertex, which typically occurs at a measurable distance from the collision point. The presence of particles that do not interact with the detector, such as neutrinos, can be inferred by summing the vector momenta of the visible particles in the plane transverse to the beam and imposing conservation of transverse momenta.

The detector components closest to the collision point measure charged-particle trajectories and momenta. This inner spectrometer is surrounded by calorimeters that are used in the identification of particles and in the measurement of their energies. The calorimeters are in turn surrounded by an outer spectrometer dedicated to measuring the trajectories and momenta of muons, the only charged particle to travel through the calorimeters. A two-level trigger system was optimized for Run 2 data-taking^26^ to select events of interest at a rate of about 1 kHz from the proton bunch collisions occurring at a rate of 40 MHz. An extensive software suite^27^ is used in the simulation, reconstruction and analysis of real and simulated data, in detector operations, and in the trigger and data-acquisition systems of the experiment.

## Input measurements and combination procedure

Physics analyses typically focus on particular production and decay processes and measure the number of Higgs boson candidates observed after accounting for non-Higgs background processes. To determine the strengths of the interactions of the Higgs boson, simultaneous fits with different physically motivated assumptions are performed on a combined set of complementary measurements. The relative weights of the input measurements in the combination depend on the analysis selection efficiencies, on the signal rates associated with the Higgs processes studied by the analysis, on the signal-to-background ratios, and on the associated systematic uncertainties.

For each decay mode entering the combination, the production process is assessed via event classification based on the properties of particles produced in association with the Higgs boson, mostly via dedicated machine-learning approaches. Unless stated otherwise, studies of each decay mode consider all individual or combined contributions from six production processes: ggF, VBF, *WH*, *ZH*, $t\bar{t}H$ and *tH*. Higgs boson interactions are further explored via additional event classification of each production process based on the kinematic properties of the produced Higgs boson and the associated particles.

The input to the combined measurement includes the latest results from the decay modes that initially led to the Higgs boson discovery: *H* → *ZZ* → *ℓ*^+^*ℓ*^−^*ℓ*^+^*ℓ*^−^ decays^28^ with two *Z* bosons that subsequently decay into a pair of oppositely charged electrons or muons; *H* → *W* ^±^*W*^∓^ → *ℓ*^±^*ν*_*ℓ*_*ℓ*^∓^*ν*_*ℓ*_ decays targeting separately the ggF and VBF^29^, and *WH* and *ZH*^30^ production processes; and *H* → *γγ* decays^31^ with two high-energy photons. The latter is the only measurement used to discriminate between the $t\bar{t}H$ and *tH* processes. These diboson decay modes are for the first time complemented by a search for the rare *H* → *Zγ* → *ℓ*^+^*ℓ*^−^*γ* decay^32^. The decays of Higgs bosons to fermions are also extensively explored. The measurement of the dominant $H\to b\bar{b}$ decay mode is particularly challenging owing to a very large multi-jet background, which can be suppressed by requiring the presence of additional particles characteristic of the *WH* or *ZH*^33,34^, VBF^35^ and $t\bar{t}H$^36^ production processes. As a new input, the fully hadronic $H\to b\bar{b}$ signal events with large Higgs boson transverse momentum are also considered^37^, providing for the first time sensitivity to the ggF production process in this decay mode. The sensitivity of the latest measurement in the *H* → *τ*^+^*τ*^−^ decay mode^38^ is now extended to the *VH* and combined $t\bar{t}H$ and *tH* production processes. In addition to the $t\bar{t}H$ measurements obtained in the *γγ*, *τ*^+^*τ*^−^ and *ZZ* decay modes, a complementary analysis that is sensitive to *τ*^+^*τ*^−^, ${W}^{\pm }{W}^{\mp }$ and *ZZ* decays is performed using events with multiple leptons in the final state^39^. The considerably more challenging measurements of Higgs boson couplings to second-generation fermions are explored via searches for the *H* → *μ*^+^*μ*^−^ decay^40^ and, included in the combination for the first time, $H\to c\bar{c}$ decay^41^. Owing to the large multi-jet background, the latter decay mode is currently accessed only via *WH* and *ZH* production. Finally, the inputs to the combination are complemented by the latest direct searches in the VBF and *ZH* production processes for Higgs boson decays into invisible particles that escape the detector^42,43^. A summary of these input measurements used in the combination is available in Extended Data Table 1.

All input measurements are performed with the full set of Run 2 data, except for the measurements of previous works^30,39^, which use a partial Run 2 dataset collected during 2015 and 2016. The direct searches for invisible Higgs boson decays and the $H\to c\bar{c}$ measurements are employed only for measurements of the relevant Higgs boson coupling strengths, and the $H\to b\bar{b}$ measurements at high Higgs boson transverse momenta^37^ are considered only when probing the kinematic properties of Higgs boson production. All other inputs are used for the measurements of production cross-sections, branching fractions and coupling strengths. The measurement of kinematic properties of Higgs boson production excludes input measurements from previous works^30,32,39–41^, owing to their limited sensitivity.

Analyses performed with the Run 2 data introduce a number of improvements, often resulting in up to 50% better signal sensitivities compared to those expected from just the increase in the analysed amount of data. These improvements include better particle reconstruction (optimized to cope with an increased number of proton interactions per bunch crossing), dedicated reconstruction of highly Lorentz-boosted $H\to b\bar{b}$ decays, a greater number of simulated events, higher granularity of the kinematic regions that are probed in each production process, and improved signal and background theory predictions.

The standard model is tested by comparing the observed signal rates to theory predictions that require state-of-the-art calculations of Higgs boson production cross-sections and branching fractions^44–50^. All signal reconstruction efficiencies and most background rates are predicted from the simulation. The simulation is complemented by the use of dedicated signal-depleted control data for measurements of selected background processes and to constrain signal-selection efficiencies. A common set of event generators were used in all analyses to describe the gluon and quark interactions in the proton–proton collisions. The generated particles were passed through a detailed simulation of the ATLAS detector response prior to their reconstruction and identification.

The statistical analysis of the data is described in more detail in Methods. It relies on a likelihood formalism, where the product of the likelihood functions describing each of the input measurements is calculated in order to obtain a combined likelihood^51^. The effects of experimental and theoretical systematic uncertainties on the predicted signal and background yields are implemented by including nuisance parameters in the likelihood function. The values of those additional parameters are either fully determined by the included data, or constrained by Gaussian terms that multiply the likelihood. The effects of uncertainties that affect multiple measurements are propagated coherently through the fit by using common nuisance parameters.

The statistical test of a given signal hypothesis, used for the measurement of the parameters of interest, is performed with a test statistic that is based on the profile likelihood ratio^52^. The confidence intervals of the measured parameters and the *p* value used to test the compatibility of the results and the standard model predictions are constructed from the test statistic distribution, which is obtained using asymptotic formulae^52^.

The total uncertainty in the measurement of a given parameter of interest can be decomposed into different components. The statistical uncertainty is obtained from a fit with all externally constrained nuisance parameters set to their best-fit values. The systematic uncertainty, the squared value of which is evaluated as the difference between the squares of the total uncertainty and the statistical uncertainty, can be decomposed into categories by setting all relevant subsets of nuisance parameters to their best-fit values.

## Combined measurement with ATLAS Run 2 data

The Higgs boson production rates are probed by the likelihood fit to observed signal yields described earlier. Because the production cross-section *σ*_*i*_ and the branching fraction *B*_*f*_ for a specific production process *i* and decay mode *f* cannot be measured separately without further assumptions, the observed signal yield for a given process is expressed in terms of a single signal strength modifier ${\mu }_{if}=({\sigma }_{i}/{\sigma }_{i}^{{\rm{SM}}})({B}_{f}/{B}_{f}^{{\rm{SM}}})$, where the superscript ‘SM’ denotes the corresponding standard model prediction. Assuming that all production and decay processes scale with the same global signal strength *μ* = *μ*_*if*_, the inclusive Higgs boson production rate relative to the standard model prediction is measured to be

$$\mu =1.05\pm 0.06=1.05\pm 0.03({\rm{stat.}})\pm 0.03({\rm{exp.}})\pm 0.04({\rm{sig.}}\,{\rm{th.}})\pm 0.02({\rm{bkg.}}\,{\rm{th.}}).$$

The total measurement uncertainty is decomposed into components for statistical uncertainties, experimental systematic uncertainties, and theory uncertainties in both signal and background modelling. Both the experimental and the theoretical uncertainties are almost a factor of two lower than in the Run 1 result^20^. The presented measurement supersedes the previous ATLAS combination with a partial Run 2 dataset^22^, decreasing the latest total measurement uncertainty by about 30%.

**Fig. 2:**
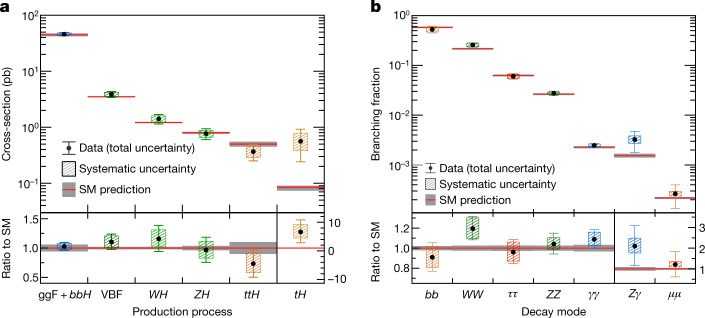
Observed and predicted Higgs boson production cross-sections and branching fractions. **a**, Cross-sections for different Higgs boson production processes are measured assuming standard model (SM) values for the decay branching fractions. **b**, Branching fractions for different Higgs boson decay modes are measured assuming SM values for the production cross-sections. The lower panels show the ratios of the measured values to their SM predictions. The vertical bar on each point denotes the 68% confidence interval. The *p* value for compatibility of the measurement and the SM prediction is 65% for **a** and 56% for **b**. Data are from ATLAS Run 2.

Higgs boson production is also studied per individual process. As opposed to the top quark decay products from $t\bar{t}H$ production, the identification efficiency of *b* jets from the $b\bar{b}H$ production is low, making the $b\bar{b}H$ process experimentally indistinguishable from ggF production. The $b\bar{b}H$ and ggF processes are therefore grouped together, with $b\bar{b}H$ contributing a relatively small amount: of the order of 1% to the total ${\rm{g}}{\rm{g}}{\rm{F}}+b\bar{b}H$ production. In cases where several processes are combined, the combination assumes the relative fractions of the components to be those from the standard model within corresponding theory uncertainties. Results are obtained from the fit to the data, where the cross-section of each production process is a free parameter of the fit. Higgs boson decay branching fractions are set to their standard model values, within the uncertainties specified previously^44^. The results are shown in Fig. 2a.

All measurement results are compatible with the standard model predictions. For the ggF and VBF production processes, which were previously observed in Run 1 data, the cross-sections are measured with a precision of 7% and 12%, respectively. The following production processes are now also observed: *WH* with an observed (expected) signal significance of 5.8 (5.1) standard deviations (*σ*), *ZH* with 5.0*σ* (5.5*σ*) and the combined $t\bar{t}H$ and *tH* production processes with 6.4*σ* (6.6*σ*), where the expected signal significances are obtained under the standard model hypothesis. The separate $t\bar{t}H$ and *tH* measurements lead to an observed (expected) upper limit on *tH* production of 15 (7) times the standard model prediction at the 95% confidence level (CL), with a relatively large negative correlation coefficient of 56% between the two measurements. This is due to cross-contamination between the $t\bar{t}H$ and *tH* processes in the set of reconstructed events that provide the highest sensitivity to these production processes.

Branching fractions of individual Higgs boson decay modes are measured by setting the cross-sections for Higgs boson production processes to their respective standard model values. The results are shown in Fig. 2b. The branching fractions of the *γγ*, *ZZ*, ${W}^{\pm }{W}^{\mp }$ and *τ*^+^*τ*^−^ decays, which were already observed in the Run 1 data, are measured with a precision ranging from 10% to 12%. The $b\bar{b}$ decay mode is observed with a signal significance of 7.0*σ* (expected 7.7*σ*), and the observed (expected) signal significances for the *H* → *μ*^+^*μ*^−^ and *H* → *Zγ* decays are 2.0*σ* (1.7*σ*) and 2.3*σ* (1.1*σ*), respectively.

**Fig. 3:**
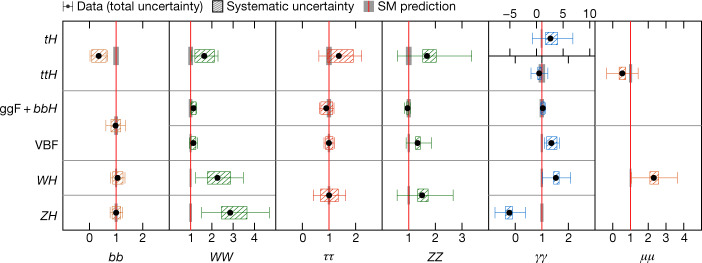
Ratio of observed rate to predicted standard model event rate for different combinations of Higgs boson production and decay processes. The horizontal bar on each point denotes the 68% confidence interval. The narrow grey bands indicate the theory uncertainties in the standard model (SM) cross-section multiplied by the branching fraction predictions. The *p* value for compatibility of the measurement and the SM prediction is 72%.  *σ*_*i*_ *B*_*f*_ is normalized to the SM prediction. Data are from ATLAS Run 2.

The assumptions about the relative contributions of different decay or production processes in the above measurements are relaxed by directly measuring the product of production cross-section and branching fraction for different combinations of production and decay processes. The corresponding results are shown in Fig. 3. The measurements are in agreement with the standard model prediction.

To determine the value of a particular Higgs boson coupling strength, a simultaneous fit of many individual production times branching fraction measurements is required. The coupling fit presented here is performed within the *κ* framework^53^ with a set of parameters **κ** that affect the Higgs boson coupling strengths without altering any kinematic distributions of a given process.

Within this framework, the cross-section times the branching fraction for an individual measurement is parameterized in terms of the multiplicative coupling strength modifiers *κ*. A coupling strength modifier *κ*_*p*_ for a production or decay process via the coupling to a given particle *p* is defined as ${\kappa }_{p}^{2}={\sigma }_{p}/{\sigma }_{p}^{{\rm{SM}}}$ or ${\kappa }_{p}^{2}={\varGamma }_{p}/{\varGamma }_{p}^{{\rm{SM}}}$, respectively, where *Γ*_*p*_ is the partial decay width into a pair of particles *p*. The parameterization takes into account that the total decay width depends on all decay modes included in the present measurements, as well as currently undetected or invisible, direct or indirect decays predicted by the standard model (such as those to gluons, light quarks or neutrinos) and the hypothetical decays into non-standard model particles. The decays to non-standard model particles are divided into decays to invisible particles and other decays that would be undetected owing to large backgrounds. The corresponding branching fractions for the two are denoted by *B*_inv._ and *B*_u._, respectively.

In the following, three classes of models with progressively fewer assumptions about coupling strength modifiers are considered. Standard model values are assumed for the coupling strength modifiers of first-generation fermions, and the modifiers of the second-generation quarks are set to those of the third generation, except where *κ*_*c*_ is left free-floating in the fit. Owing to their small sizes, these couplings are not expected to noticeably affect any of the results. The ggF production and the *H* → *γγ* and *H* → *Zγ* decays are loop-induced processes. They are either expressed in terms of the more fundamental coupling strength scale factors corresponding to the particles that contribute to the loop-induced processes in the standard model, or treated using effective coupling strength modifiers *κ*_*g*_, *κ*_*γ*_ and *κ*_*Zγ*_, respectively. The latter scenario accounts for possible loop contributions from particles beyond the standard model. The small contribution from the loop-induced *gg* → *ZH* process is always parameterized in terms of the couplings to the corresponding standard model particles.

**Fig. 4:**
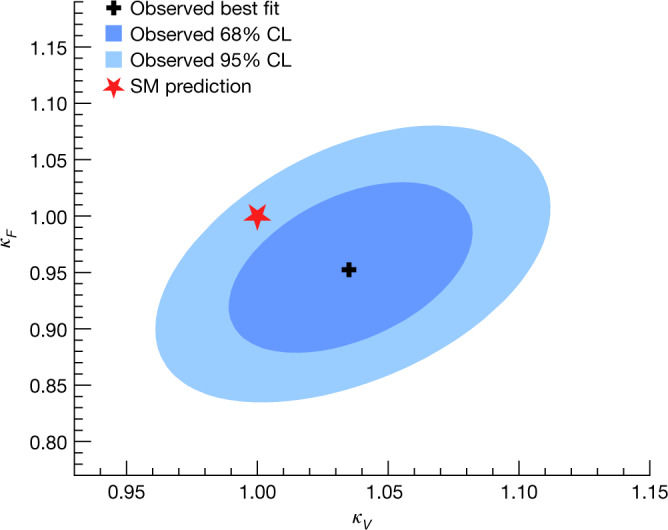
Negative log-likelihood contours corresponding to 68% and 95% CL in the (*κ*_*V*_, *κ*_*F*_) plane. The data are obtained from a combined fit assuming no contributions from invisible or undetected non-standard model Higgs boson decays. The *p* value for compatibility of the combined measurement and the standard model (SM) prediction is 14%. Data are from ATLAS Run 2.

The first model tests one scale factor for the vector bosons, *κ*_*V*_ = *κ*_*W*_ = *κ*_*Z*_, and a second, *κ*_*F*_, which applies to all fermions. In general, the standard model prediction of *κ*_*V*_ = *κ*_*F*_ = 1 does not hold in extensions of the standard model. For example, the values of *κ*_*V*_ and *κ*_*F*_ would be less than 1 in models in which the Higgs boson is a composite particle. The effective couplings corresponding to the ggF, *H* → *γγ* and *H* → *Zγ* loop-induced processes are parameterized in terms of the fundamental standard model couplings. It is assumed that there are no invisible or undetected Higgs boson decays beyond the standard model, that is, *B*_inv._ = *B*_u._ = 0. As only the relative sign between *κ*_*V*_ and *κ*_*F*_ is physical and a negative relative sign has been excluded with a high level of confidence^20^, *κ*_*V*_ ≥ 0 and *κ*_*F*_ ≥ 0 are assumed. Figure 4 shows the results of a combined fit in the (*κ*_*V*_, *κ*_*F*_) plane. The best-fit values and their uncertainties from the combined fit are *κ*_*V*_ = 1.035 ± 0.031 and *κ*_*F*_ = 0.95 ± 0.05, compatible with the standard model predictions. A relatively large positive correlation of 39% is observed between the two fit parameters, because some of the most sensitive input measurements involve the ggF production process (that is, via couplings to fermions) with subsequent Higgs boson decays into vector bosons.

**Fig. 5:**
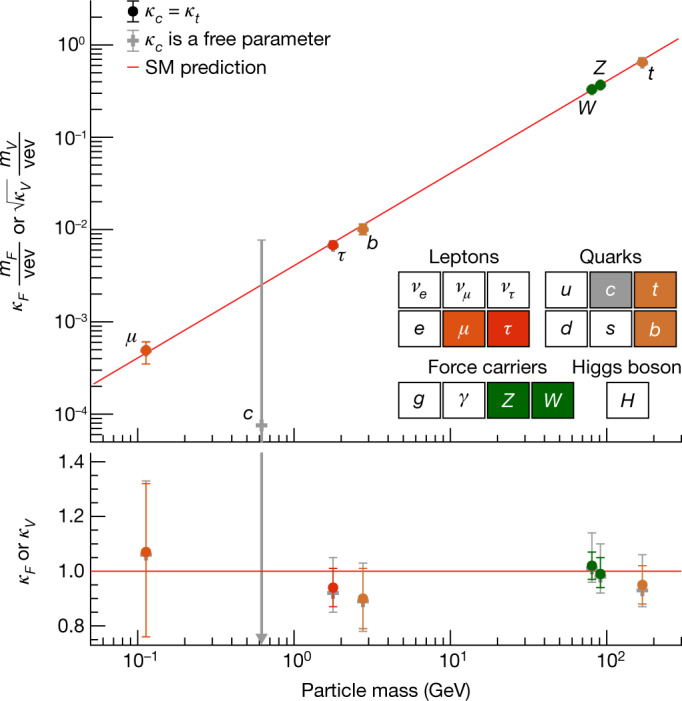
Reduced Higgs boson coupling strength modifiers and their uncertainties. They are defined as *κ*_*F*_*m*_*F*_/vev for fermions (*F* = *t*, *b*, *τ*, *μ*) and $\sqrt{{\kappa }_{V}}{m}_{V}/{\rm{vev}}$ for vector bosons as a function of their masses *m*_*F*_ and *m*_*V*_. Two fit scenarios with *κ*_*c*_ = *κ*_*t*_ (coloured circle markers), or *κ*_*c*_ left free-floating in the fit (grey cross markers) are shown. Loop-induced processes are assumed to have the standard model (SM) structure, and Higgs boson decays to non-SM particles are not allowed. The vertical bar on each point denotes the 68% confidence interval. The *p* values for compatibility of the combined measurement and the SM prediction are 56% and 65% for the respective scenarios. The lower panel shows the values of the coupling strength modifiers. The grey arrow points in the direction of the best-fit value and the corresponding grey uncertainty bar extends beyond the lower panel range. Data are from ATLAS Run 2.

In the second class of models, the coupling strength modifiers for *W*, *Z*, *t*, *b*, *c*, *τ* and *μ* are treated independently. All modifiers are assumed to be positive. It is assumed that only standard model particles contribute to the loop-induced processes, and modifications of the fermion and vector boson couplings are propagated through the loop calculations. Invisible or undetected non-standard model Higgs boson decays are not considered. These models enable testing of the predicted scaling of the couplings of the Higgs boson to the standard model particles as a function of their mass using the reduced coupling strength modifiers $\sqrt{{\kappa }_{V}{g}_{V}/2{\rm{vev}}}=\sqrt{{\kappa }_{V}}({m}_{V}/{\rm{vev}})$ for weak bosons with a mass *m*_*V*_ and *κ*_*F*_*g*_*F*_ = *κ*_*F*_*m*_*F*_/vev for fermions with a mass *m*_*F*_, where *g*_*V*_ and *g*_*F*_ are the corresponding absolute coupling strengths and ‘vev’ is the vacuum expectation value of the Higgs field. Figure 5 shows the results for two scenarios: one with the coupling to *c* quarks constrained by *κ*_*c*_ = *κ*_*t*_ in order to cope with the low sensitivity to this coupling; and the other with *κ*_*c*_ left as a free parameter in the fit. All measured coupling strength modifiers are found to be compatible with their standard model prediction. When the coupling strength modifier *κ*_*c*_ is left unconstrained in the fit, an upper limit of *κ*_*c*_ < 5.7 (7.6) times the standard model prediction is observed (expected) at 95% CL and the uncertainty in each of the other parameters increases because of the resulting weaker constraint on the total decay width. This improves the current observed (expected) limit of *κ*_*c*_ < 8.5 (12.4) at 95% CL from the individual measurement of $H\to c\bar{c}$ decays^41^ despite the relaxed assumptions on other coupling strength modifiers, through constraints coming from the parameterization of the total Higgs boson decay width that impacts all measurements.

**Fig. 6:**
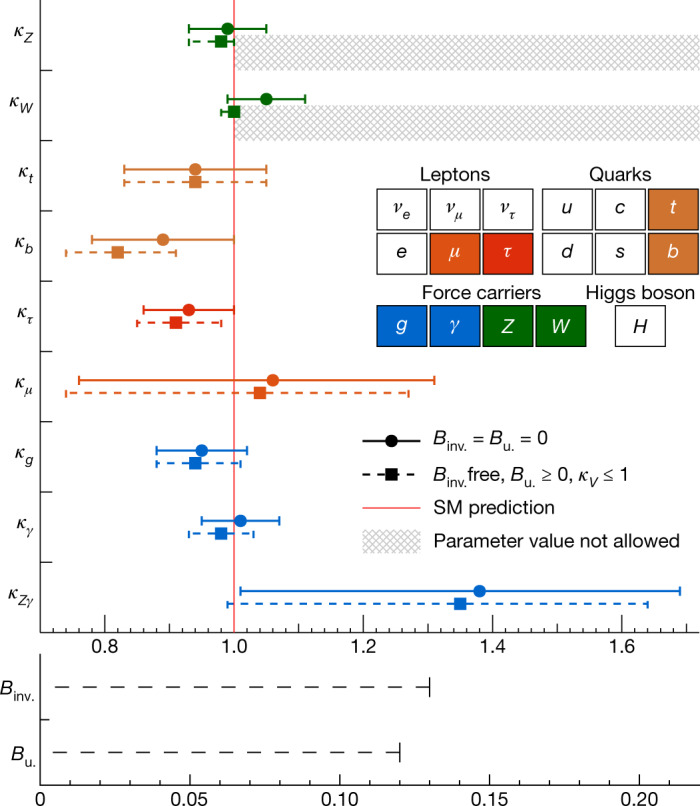
Reduced coupling strength modifiers and their uncertainties per particle type with effective photon, *Zγ* and gluon couplings. The horizontal bars on each point denote the 68% confidence interval. The scenario in which *B*_inv._ = *B*_u._ = 0 is assumed is shown as solid lines with circle markers. The *p* value for compatibility with the standard model (SM) prediction is 61% in this case. The scenario in which *B*_inv._ and *B*_u._ are allowed to contribute to the total Higgs boson decay width while assuming that *κ*_*V*_ ≤ 1 and *B*_u._ ≥ 0 is shown as dashed lines with square markers. The lower panel shows the 95% CL upper limits on *B*_inv._ and *B*_u._. Data are from ATLAS Run 2.

The third class of models in the *κ* framework closely follows the previous one, but allows for the presence of non-standard model particles in the loop-induced processes. These processes are parameterized by the effective coupling strength modifiers *κ*_*g*_, *κ*_*γ*_ and *κ*_*Zγ*_ instead of propagating modifications of the standard model particle couplings through the loop calculations. It is also assumed that any potential effect beyond the standard model does not substantially affect the kinematic properties of the Higgs boson decay products. The fit results for the scenario in which invisible or undetected non-standard model Higgs boson decays are assumed not to contribute to the total Higgs decay width, that is, *B*_inv._ = *B*_u._ = 0, are shown in Fig. 6 together with the results for the scenario allowing such decays. To avoid degenerate solutions, the latter constrains *B*_u._ ≥ 0 and imposes the additional constraint *κ*_*V*_ ≤ 1 that naturally arises in various scenarios of physics beyond the standard model^54,55^. All measured coupling strength modifiers are compatible with their standard model predictions.

When allowing invisible or undetected non-standard model Higgs boson decays to contribute to the total Higgs boson decay width, the previously measured coupling strength modifiers do not change significantly, and upper limits of *B*_u._ < 0.12 (expected 0.21) and *B*_inv._ < 0.13 (expected 0.08) are set at 95% CL on the corresponding branching fraction. The latter improves on the current best limit of *B*_inv._ < 0.145 (expected 0.103) from direct ATLAS searches^42^.

In all tested scenarios, the statistical and the systematic uncertainty contribute almost equally to the total uncertainty in most of the *κ* parameter measurements. The exceptions are the *κ*_*μ*_, *κ*_*Zγ*_, *κ*_*c*_ and *B*_u._ measurements, for which the statistical uncertainty still dominates.

Kinematic properties of Higgs boson production probing the internal structure of its couplings are studied in the framework of simplified template cross-sections^44,56–58^. The framework partitions the phase space of standard model Higgs boson production processes into a set of regions defined by the specific kinematic properties of the Higgs boson and, where relevant, of the associated jets, *W* bosons, or *Z* bosons, as described in Methods. The regions are defined so as to provide experimental sensitivity to deviations from the standard model predictions, to avoid large theory uncertainties in these predictions, and to minimize the model-dependence of their extrapolations to the experimentally accessible signal regions. Signal cross-sections measured in each of the introduced kinematic regions are compared with those predicted when assuming that the branching fractions and kinematic properties of the Higgs boson decay are described by the standard model.

**Fig. 7:**
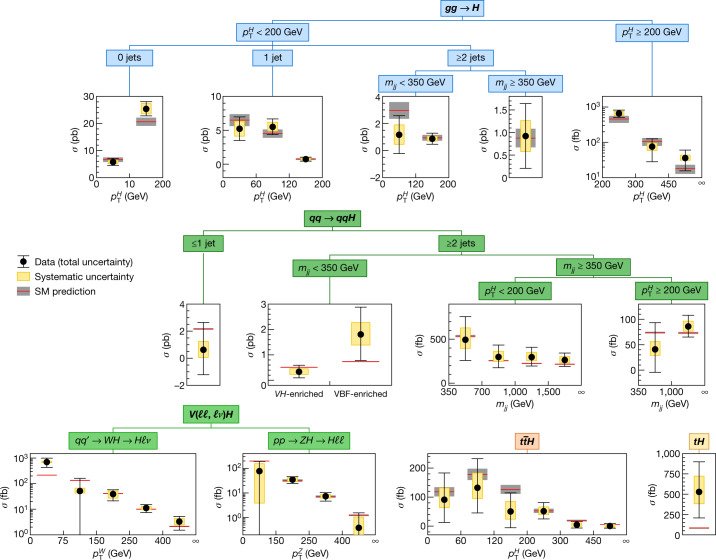
Observed and predicted Higgs boson production cross-sections in different kinematic regions. The vertical bar on each point denotes the 68% confidence interval. The *p* value for compatibility of the combined measurement and the standard model (SM) prediction is 94%. Kinematic regions are defined separately for each production process, based on the jet multiplicity, the transverse momentum of the Higgs $({p}_{{\rm{T}}}^{H})$ and vector bosons (${p}_{{\rm{T}}}^{W}$ and ${p}_{{\rm{T}}}^{Z}$) and the two-jet invariant mass (*m*_*jj*_). The ‘*VH*-enriched’ and ‘VBF-enriched’ regions with the respective requirements of ${m}_{jj}\in [60,120)\,{\rm{GeV}}$ and ${m}_{jj}\notin [60,120)\,{\rm{GeV}}$ are enhanced in signal events from *VH* and VBF productions, respectively. Data are from ATLAS Run 2.

The results of the simultaneous measurement in 36 kinematic regions are presented in Fig. 7. Compared to previous results with a smaller dataset^22^, a much larger number of regions are probed, particularly at high Higgs boson transverse momenta, where in many cases the sensitivity to new phenomena beyond the standard model is expected to be enhanced. All measurements are consistent with the standard model predictions.

## Conclusion

In summary, the production and decay rates of the Higgs boson were measured using the dataset collected by the ATLAS experiment during Run 2 of the LHC from 2015 to 2018. The measurement results were found to be in excellent agreement with the predictions of the standard model. In different scenarios, the couplings to the three heaviest fermions, the top quark, the *b* quark and the *τ* lepton, were measured with uncertainties ranging from about 7% to 12% and the couplings to the weak bosons (*Z* and *W*) were measured with uncertainties of about 5%. In addition, indications are emerging of the presence of very rare Higgs boson decays into second-generation fermions and into a *Z* boson and a photon. Finally, a comprehensive study of Higgs boson production kinematics was performed and the results were also found to be compatible with standard model predictions. In the ten years since its discovery, the Higgs boson has undergone many experimental tests that have demonstrated that, so far, its nature is remarkably consistent with the predictions of the standard model. However, some of its key properties—such as the coupling of the Higgs boson to itself—remain to be measured. In addition, some of its rare decay modes have not yet been observed and there is ample room for new phenomena beyond the standard model to be discovered. Substantial progress on these fronts is expected in the future, given that detector upgrades are planned for the coming years, that systematic uncertainties are expected to be reduced considerably^59^, and that the size of the LHC’s dataset is projected to increase by a factor of 20.

## Methods

### Experimental set-up

The ATLAS detector^12^ consists of an inner tracking detector surrounded by a thin superconducting solenoid, electromagnetic and hadron calorimeters, and a muon spectrometer incorporating three large superconducting air-core toroidal magnets.

ATLAS uses a right-handed coordinate system with its origin at the nominal interaction point in the centre of the detector and the *z* axis along the beam pipe. The *x* axis points from the interaction point to the centre of the LHC ring, and the *y* axis points upwards. Cylindrical coordinates (*r*, *ϕ*) are used in the transverse plane, *ϕ* being the azimuthal angle around the *z* axis. The pseudorapidity is defined in terms of the polar angle *θ* as *η* = −ln(tan(*θ*/2)).

The inner-detector (ID) system is immersed in a 2-T axial magnetic field and provides charged-particle tracking in the range |*η*| < 2.5. The high-granularity silicon pixel detector covers the vertex region and typically provides four measurements per track, the first hit normally being in the insertable B-layer (IBL) installed before Run 2^60,61^. It is followed by the silicon microstrip tracker (SCT), which usually provides eight measurements per track. These silicon detectors are complemented by the transition radiation tracker (TRT), which enables radially extended track reconstruction up to |*η*| < 2.0. The TRT also provides electron identification information based on the fraction of hits (typically 30 in total) above a higher energy-deposit threshold corresponding to transition radiation.

The calorimeter system covers the pseudorapidity range |*η*| < 4.9. Within the region |*η*| < 3.2, electromagnetic calorimetry is provided by barrel and endcap high-granularity lead/liquid-argon (LAr) calorimeters, with an additional thin LAr presampler covering |*η*| < 1.8 to correct for energy loss in material upstream of the calorimeters. Hadron calorimetry is provided by the steel/scintillator-tile calorimeter, segmented into three barrel structures within |*η*| < 1.7, and two copper/LAr hadron endcap calorimeters. The solid angle coverage is completed with forward copper/LAr and tungsten/LAr calorimeter modules optimized for electromagnetic and hadronic energy measurements, respectively.

The muon spectrometer (MS) comprises separate trigger and high-precision tracking chambers measuring the deflection of muons in a magnetic field generated by the superconducting air-core toroidal magnets. The field integral of the toroids ranges between 2.0 and 6.0 Tm across most of the detector. Three layers of precision chambers, each consisting of layers of monitored drift tubes, covers the region |*η*| < 2.7, complemented by cathode-strip chambers in the forward region, where the background is highest. The muon trigger system covers the range |*η*| < 2.4 with resistive-plate chambers in the barrel, and thin-gap chambers in the endcap regions.

The performance of the vertex and track reconstruction in the inner detector, the calorimeter resolution in electromagnetic and hadronic calorimeters and the muon momentum resolution provided by the muon spectrometer are given previously^12^.

Interesting events are selected by the first-level trigger system implemented in custom hardware, followed by selections made by algorithms implemented in software in the high-level trigger^62^. The first-level trigger accepts events from the 40-MHz bunch crossings at a rate below 100 kHz, which the high-level trigger further reduces in order to record events to disk at about 1 kHz.

### Statistical framework

The results of the combination presented in this paper are obtained from a likelihood function defined as the product of the likelihoods of each input measurement. The observed yield in each category of reconstructed events follows a Poisson distribution the parameter of which is the sum of the expected signal and background contributions. The number of signal events in any category *k* is split into the different production and decay modes:

$${n}_{k}^{{\rm{signal}}}={{\mathcal{L}}}_{k}\sum _{i}\sum _{f}({\sigma }_{i}{B}_{f}){(A\epsilon )}_{if}^{k},$$

where the sum indexed by *i* runs either over the production processes (ggF, VBF, *WH*, *ZH*, $t\bar{t}H$, *tH*) or over the set of the measured production kinematic regions, and the sum indexed by *f* runs over the decay final states (*ZZ*, *WW*, *γγ*, *Zγ*, $b\bar{b}$, $c\bar{c}$, *τ*^+^*τ*^−^, *μ*^+^*μ*^−^). The quantity ${ {\mathcal L} }_{k}$ is the integrated luminosity of the dataset used in category *k*, and ${(A\epsilon )}_{if}^{k}$ is the acceptance times selection efficiency factor for production process *i* and decay mode *f* in category *k*. Acceptances and efficiencies are obtained from the simulation (corrected by calibration measurements in control data for the efficiencies). Their values are subject to variations due to experimental and theoretical systematic uncertainties. The cross-sections *σ*_*i*_ and branching fractions *B*_*f*_ are the parameters of interest of the model. Depending on the model being tested, they are either free parameters, set to their standard model prediction or parameterized as functions of other parameters. All cross-sections are defined in the Higgs boson rapidity range |*y*_*H*_| < 2.5, which is related to the polar angle of the Higgs boson’s momentum in the detector and corresponds approximately to the region of experimental sensitivity.

The impact of experimental and theoretical systematic uncertainties on the predicted signal and background yields is taken into account by nuisance parameters included in the likelihood function. The predicted signal yields from each production process, the branching fractions and the signal acceptance in each analysis category are affected by theory uncertainties. The combined likelihood function is therefore expressed as:

$$L({\boldsymbol{\alpha }},{\boldsymbol{\theta }},{\rm{d}}{\rm{a}}{\rm{t}}{\rm{a}})=\prod _{k\in {\rm{c}}{\rm{a}}{\rm{t}}}\prod _{b\in {\rm{b}}{\rm{i}}{\rm{n}}{\rm{s}}}P({n}_{k,b}|{n}_{k,b}^{{\rm{s}}{\rm{i}}{\rm{g}}{\rm{n}}{\rm{a}}{\rm{l}}}({\boldsymbol{\alpha }},{\boldsymbol{\theta }})+{n}_{k,b}^{{\rm{b}}{\rm{k}}{\rm{g}}}({\boldsymbol{\theta }}))\prod _{\theta \in {\boldsymbol{\theta }}}G(\theta ),$$

where *n*_*k,b*_, ${n}_{k,b}^{{\rm{signal}}}$ and ${n}_{k,b}^{{\rm{bkg}}}$ stand for the number of observed events, the number of expected signal events and the number of expected background events in bin *b* of analysis category *k*, respectively. The parameters of interest are noted **α**, the nuisance parameters are **θ**, *P* represents the Poisson distribution, and *G* stands for Gaussian constraint terms assigned to the nuisance parameters. Some nuisance parameters are meant to be determined by data alone and do not have any associated constraint term. This is, for instance, the case for background normalization factors that are fitted in control categories. The effects of nuisance parameters affecting the normalizations of signal and backgrounds in a given category are generally implemented using the multiplicative expression:

$$n(\theta )={n}^{0}{(1+\sigma )}^{\theta },$$

where *n*^0^ is the nominal expected yield of either signal or background and *σ* the value of the uncertainty. This ensures that *n*(*θ*) > 0 even for negative values of *θ*. For the majority of nuisance parameters, including all those affecting the shapes of the distributions, a linear expression is used instead on each bin of the distributions:

$$n(\theta )={n}^{0}(1+\sigma \theta ).$$

The systematic uncertainties are broken down into independent underlying sources, so that when a source affects multiple or all analyses the associated nuisance parameter can be fully correlated across the terms in the likelihood corresponding to these analyses by using common nuisance parameters. This is the case of systematic uncertainties in the luminosity measurement^63^, in the reconstruction and selection efficiencies^64–70^ and in the calibrations of the energy measurements^71–74^. Their effects are propagated coherently by using common nuisance parameters whenever applicable. Only a few components of the systematic uncertainties are correlated between the analyses performed using the full Run 2 data and those using only the 2015 and 2016 data, owing to differences in their assessment, in the reconstruction algorithms and in software releases. Systematic uncertainties associated with the modelling of background processes, as well as uncertainties due to the limited number of simulated events used to estimate the expected signal and background yields, are treated as being uncorrelated between analyses.

Uncertainties in the parton distribution functions are implemented coherently in all input measurements and all analysis categories^75^. Uncertainties in modelling the parton showering into jets of particles affect the signal acceptances and efficiencies, and are common to all input measurements within a given production process. Similarly, uncertainties due to missing higher-order quantum chromodynamics (QCD) corrections are common to a given production process. Their implementation in the kinematic regions of the simplified template cross-sections framework results in a total of 66 uncertainty sources, where overall acceptance effects are separated from migrations between the various bins (for example, between jet multiplicity regions or between dijet invariant mass regions)^76^. Both the acceptance and signal yield uncertainties affect the signal strength modifier and coupling strength modifier results, which rely on comparisons of measured and expected yields. Only acceptance uncertainties affect the cross-section and branching fraction results. The uncertainties in the Higgs boson branching fractions due to dependencies on standard model parameter values (such as *b* and *c* quark masses) and missing higher-order effects are implemented using the correlation model described previously^44^.

In total, over 2,600 sources of systematic uncertainty are included in the combined likelihood. For most of the presented measurements, the systematic uncertainty is expected to be of similar size or somewhat smaller than the corresponding statistical uncertainty. The systematic uncertainties are dominant for the parameters that are measured the most precisely, that is, the global signal strength and the production cross-sections for the ggF and VBF processes. The expected systematic uncertainty of the global signal strength measurement (about 5%) is larger than the statistical uncertainty (3%), with similar contributions from the theory uncertainties in signal (4%) and background modelling (1.7%), and from the experimental systematic uncertainty (3%). The latter is predominantly composed of the uncertainty in the luminosity measurement (1.7%), followed by the uncertainties in electron, jet and *b*-jet reconstruction, data-driven background modelling, as well as from the limited number of simulated events (about 1% each). All other sources of experimental uncertainty combined contribute an additional 1%. The systematic uncertainty in the production cross-section of the ggF process is dominated by experimental uncertainties (3.5%) followed by signal theory uncertainties (3%), compared to a statistical uncertainty of 4%. For the VBF process, where the statistical uncertainty is 8%, the experimental uncertainties are estimated to be 5%, and the signal theory uncertainties add up to 7%. Systematic uncertainties are also dominant over the statistical uncertainties in the measurements of the branching fractions into *W* pairs and *τ* lepton pairs.

Measurements of the parameters of interest use a statistical test based on the profile likelihood ratio^52^:

$$\varLambda ({\boldsymbol{\alpha }})=\frac{L({\boldsymbol{\alpha }},\hat{\hat{{\boldsymbol{\theta }}}}({\boldsymbol{\alpha }}))}{L(\hat{{\boldsymbol{\alpha }}},\hat{{\boldsymbol{\theta }}})},$$

where **α** are the parameters of interest and **θ** are the nuisance parameters. The $\hat{\hat{{\boldsymbol{\theta }}}}({\boldsymbol{\alpha }})$ notation indicates that the nuisance parameters values are those that maximize the likelihood for given values of the parameters of interest. In the denominator, both the parameters of interest and the nuisance parameters are set to the values ($\hat{{\boldsymbol{\alpha }}}$, $\hat{{\boldsymbol{\theta }}}$) that unconditionally maximize the likelihood. The estimates of the parameters **α** are these values $\hat{{\boldsymbol{\alpha }}}$ that maximize the likelihood ratio.

Owing to the usually large number of events selected in the measurements, all results presented in this paper are obtained in the asymptotic regime where the likelihood approximately follows a Gaussian distribution. It was checked in previous iterations of the individual input measurements, for instance ref. ^77^, that this assumption also holds in cases with low event counts by comparing the results of the asymptotic formulae with those of pseudo-experiments. This confirmed the results from a previous work^52^ that the Gaussian approximation becomes valid for as few as ≳5 background events. In the asymptotic regime twice the negative logarithm of the profile likelihood *λ*(**α**) = −2ln(*Λ*(**α**)) follows a *χ*^2^ distribution with a number of degrees of freedom equal to the number of parameters of interest. Confidence intervals for a given confidence level (CL), usually 68%, are then defined as the regions fulfilling $\lambda ({\boldsymbol{\alpha }}) < {F}_{n}^{-1}({\rm{C}}{\rm{L}})$ where ${F}_{n}^{-1}$ is the quantile function of the *χ*^2^ distribution with *n* degrees of freedom, so ${F}_{1}^{-1}=1\,(4)$ for a 1*σ* (2*σ*) CL with one degree of freedom. The values of the parameters **α** corresponding to these confidence intervals are obtained by scanning the profile likelihood. Similarly, the *p* value *p*_SM_ = 1 − *F*_*n*_(*λ*(**α**_SM_)) is used to test the compatibility of the measurement and the standard model prediction. The correlations between the parameters are estimated by inverting the matrix of the second derivatives of the likelihood.

The expected significances and limits are determined using the ‘Asimov’ datasets^52^, which are obtained by setting the observed yields to their expected values when the nuisance parameters are set to the values that maximize the likelihood $\hat{{\rm{\theta }}}$.

### Parameterization within the *κ* framework

Within the *κ* framework, the cross-section for an individual measurement is parameterized as

$$\sigma (i\to H\to f)={\sigma }_{i}{B}_{f}=\frac{{\sigma }_{i}({\boldsymbol{\kappa }}){\varGamma }_{f}({\boldsymbol{\kappa }})}{{\varGamma }_{H}({\boldsymbol{\kappa }},{B}_{{\rm{inv.}}},{B}_{{\rm{u.}}})},$$

where *Γ*_*f*_ is the partial width for a Higgs boson decay to the final state *f* and *Γ*_*H*_ is the total decay width of the Higgs boson. The total width is given by the sum of the partial widths of all the decay modes included. Contributions to the total Higgs boson decay width owing to phenomena beyond the standard model may manifest themselves as a value of coupling strength modifier *κ*_*p*_ differing from one, or a value of *B*_inv__._ or *B*_u._ differing from zero. The Higgs boson total width is then expressed as ${\varGamma }_{H}({\boldsymbol{\kappa }},{B}_{{\rm{inv.}}},{B}_{{\rm{u.}}})={\kappa }_{H}^{2}({\boldsymbol{\kappa }},{B}_{{\rm{inv.}}},{B}_{{\rm{u.}}}){\varGamma }_{H}^{{\rm{SM}}}$ with

$${\kappa }_{H}^{2}({\boldsymbol{\kappa }},{B}_{{\rm{inv.}}},{B}_{{\rm{u.}}})=\frac{{\sum }_{p}{B}_{p}^{{\rm{SM}}}{\kappa }_{p}^{2}}{(1-{B}_{{\rm{inv.}}}-{B}_{{\rm{u.}}})}.$$

Higgs boson production cross-sections and partial and total decay widths are parameterized in terms of the coupling strength modifiers as shown in table 9 of ref. ^22^. An improved parameterization including additional sub-leading contributions is used in this paper to match the increased precision of the measurements.

### Kinematic regions probing Higgs boson production

The definitions of kinematic regions for the precision study of Higgs boson production in the framework of simplified template cross-sections^44,56–58^ are based on the predicted properties of particles generated in a given production process. The partitioning follows the so-called Stage-1.2 scheme, which features a slightly finer granularity than the Stage-1.1 scheme^57^ and introduces the Higgs boson transverse momentum categories for the $t\bar{t}H$ production process. Higgs bosons are required to be produced with rapidity |*y*_*H*_| < 2.5. Associated jets of particles are constructed from all stable particles with a lifetime greater than 10 ps, excluding the decay products of the Higgs boson and leptons from *W* and *Z* boson decays, using the anti-*k*_*t*_ algorithm^78^ with a jet radius parameter *R* = 0.4, and must have a transverse momentum *p*_T,jet_ > 30 GeV. Standard model predictions are assumed for the kinematic properties of Higgs boson decays. Phenomena beyond the standard model can substantially modify these properties, and thus the acceptance of the signal, especially for the *WW* or *ZZ* decay modes, and this should be considered when using these measurements for the relevant interpretations.

Higgs boson production is first classified according to the nature of the initial state and the associated particles, the latter including the decay products of *W* and *Z* bosons if they are present. These classes are: $t\bar{t}H$ and *tH* processes; *qq*′ → *Hqq*′ processes, with contributions from both VBF and quark-initiated *VH* (where *V* = *W*, *Z*) production with a hadronic decay of the vector boson; *pp* → *VH* production with a leptonic decay of the vector boson (*V*(*ℓℓ*, *ℓν*)*H*), including *gg* → *ZH* → *ℓℓH* production; and finally the ggF process combined with $gg\to ZH\to q\bar{q}H$ production to form a single *gg* → *H* process. The contribution of the $b\bar{b}H$ production process is taken into account as a 1%^44^ increase of the *gg* → *H* yield in each kinematic region, because the acceptances for both processes are similar for all input analyses^44^.

The input measurements in individual decay modes provide only limited sensitivity to the cross-section in some of the regions of the Stage-1.2 scheme, mainly because of the small number of events in some of these regions. In other cases, they only provide sensitivity to a combination of these regions, leading to strongly correlated measurements. To mitigate these effects, some of the Stage-1.2 kinematic regions were merged for the combined measurement.

Compared to individual input measurements, systematic theory uncertainties associated with the signal predictions have been updated for the combination to closely follow the granularity of the Stage-1.2 scheme. The QCD scale uncertainties in ggF production were updated for all input channels that are sensitive to this production process. Out of 18 uncertainty sources in total, two account for overall fixed-order and resummation effects, two cover the migrations between different jet multiplicity bins, seven are associated with the modelling of the Higgs boson transverse momentum (${p}_{{\rm{T}}}^{H}$) in different phase-space regions, four account for the uncertainty in the distribution of the dijet invariant mass (*m*_*jj*_) variable, one covers the modelling of the Higgs boson plus two leading jets transverse momentum (${p}_{{\rm{T}}}^{Hjj}$) distribution in the ≥2-jet region, one pertains to modelling of the distribution of the Higgs boson plus one jet transverse momentum (${p}_{{\rm{T}}}^{Hj}$) divided by ${p}_{{\rm{T}}}^{H}$ in the high-${p}_{{\rm{T}}}^{H}$ region, and finally, the last takes into account the uncertainty from the choice of top quark mass scheme. Theory uncertainties for the *qq*′ → *Hqq*′ and $t\bar{t}H$ processes are defined previously^28^, and those of the *V*(*ℓℓ*, *ℓν*)*H* kinematic region follow the scheme described in an earlier work^76^. For the kinematic regions defined by the merging of several Stage-1.2 regions, the signal acceptance factors are determined assuming that the relative fractions in each Stage-1.2 region are given by their standard model values, and the uncertainties predicted by the standard model in these fractions are taken into account.

## Online content

Any methods, additional references, Nature Research reporting summaries, source data, extended data, supplementary information, acknowledgements, peer review information; details of author contributions and competing interests; and statements of data and code availability are available at 10.1038/s41586-022-04893-w.

## Figures and Tables

**Extended Data Table 1:**
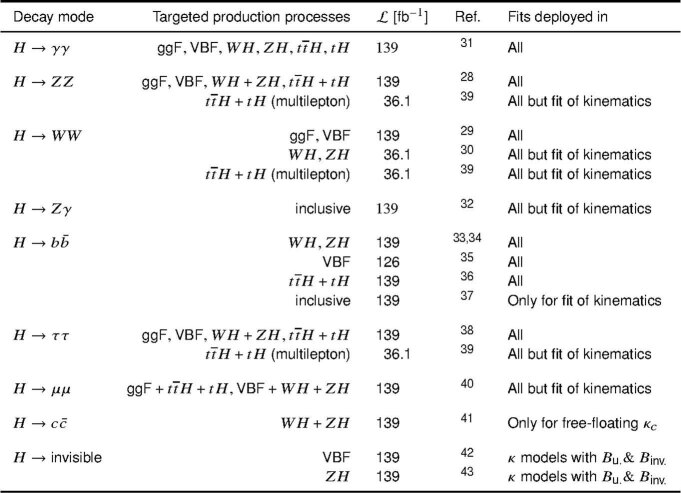
Summary of the input measurements used for the combined fit results

Listed are the measured decay modes, targeted production processes and integrated luminosity (${\mathcal{L}}$) used for each input analysis of the combination. The references for the input measurements and information about which combined measurements they enter are also provided. The $q\bar{q}/gg\to t\bar{t}H+tHX$ measurement in the multilepton final state^39^ is complementary to other $q\bar{q}/gg\to t\bar{t}H+tHX$ measurements in the *WW*, *ZZ* and *ττ* decay modes, with no overlapping events selected by these measurements.

## Data Availability

The experimental data that support the findings of this study are available in HEPData with the identifier 10.17182/hepdata.130266.

## Code availability

The ATLAS data reduction software is available at the following link: https://zenodo.org/record/4772550. Statistical modelling and analysis is based on the ROOT software and its embedded RooFit and RooStats modules, available at https://zenodo.org/record/3895852. Code to configure these statistical tools and to process their output is available upon request.
