# Publisher Correction: A detailed map of Higgs boson interactions by the ATLAS experiment ten years after the discovery

**DOI:** 10.1038/s41586-022-05581-5

**Published:** 2022-12-06

**Authors:** The ATLAS Collaboration

**Affiliations:** ATLAS Experiment, CERN, Geneva, Switzerland

**Keywords:** Particle physics, Experimental particle physics

Correction to: *Nature* 10.1038/s41586-022-04893-w Published online 4 July 2022

In the version of this article initially published, there was an error in the top *y*-axis label of Fig. 5, where in “${\kappa }_{F\frac{{m}_{F}}{{\rm{vev}}}}$ ”, the numerator “*m*_*F*_” appeared in error as “*m*_*V*_”; the label has been amended in the HTML and PDF versions of the article.

