# Author Correction: A detailed map of Higgs boson interactions by the ATLAS experiment ten years after the discovery

**DOI:** 10.1038/s41586-023-06248-5

**Published:** 2023-10-18

**Authors:** The ATLAS Collaboration, G. Aad, B. Abbott, D. C. Abbott, K. Abeling, S. H. Abidi, A. Aboulhorma, H. Abramowicz, H. Abreu, Y. Abulaiti, A. C. Abusleme Hoffman, B. S. Acharya, B. Achkar, L. Adam, C. Adam Bourdarios, L. Adamczyk, L. Adamek, S. V. Addepalli, J. Adelman, A. Adiguzel, S. Adorni, T. Adye, A. A. Affolder, Y. Afik, M. N. Agaras, J. Agarwala, A. Aggarwal, C. Agheorghiesei, J. A. Aguilar-Saavedra, A. Ahmad, F. Ahmadov, W. S. Ahmed, S. Ahuja, X. Ai, G. Aielli, I. Aizenberg, M. Akbiyik, T. P. A. Åkesson, A. V. Akimov, K. Al Khoury, G. L. Alberghi, J. Albert, P. Albicocco, M. J. Alconada Verzini, S. Alderweireldt, M. Aleksa, I. N. Aleksandrov, C. Alexa, T. Alexopoulos, A. Alfonsi, F. Alfonsi, M. Alhroob, B. Ali, S. Ali, M. Aliev, G. Alimonti, C. Allaire, B. M. M. Allbrooke, P. P. Allport, A. Aloisio, F. Alonso, C. Alpigiani, E. Alunno Camelia, M. Alvarez Estevez, M. G. Alviggi, Y. Amaral Coutinho, A. Ambler, C. Amelung, C. G. Ames, D. Amidei, S. P. Amor Dos Santos, S. Amoroso, K. R. Amos, C. S. Amrouche, V. Ananiev, C. Anastopoulos, N. Andari, T. Andeen, J. K. Anders, S. Y. Andrean, A. Andreazza, S. Angelidakis, A. Angerami, A. V. Anisenkov, A. Annovi, C. Antel, M. T. Anthony, E. Antipov, M. Antonelli, D. J. A. Antrim, F. Anulli, M. Aoki, J. A. Aparisi Pozo, M. A. Aparo, L. Aperio Bella, C. Appelt, N. Aranzabal, V. Araujo Ferraz, C. Arcangeletti, A. T. H. Arce, E. Arena, J-F. Arguin, S. Argyropoulos, J.-H. Arling, A. J. Armbruster, O. Arnaez, H. Arnold, Z. P. Arrubarrena Tame, G. Artoni, H. Asada, K. Asai, S. Asai, N. A. Asbah, E. M. Asimakopoulou, J. Assahsah, K. Assamagan, R. Astalos, R. J. Atkin, M. Atkinson, N. B. Atlay, H. Atmani, P. A. Atmasiddha, K. Augsten, S. Auricchio, A. D. Auriol, V. A. Austrup, G. Avner, G. Avolio, K. Axiotis, M. K. Ayoub, G. Azuelos, D. Babal, H. Bachacou, K. Bachas, A. Bachiu, F. Backman, A. Badea, P. Bagnaia, M. Bahmani, A. J. Bailey, V. R. Bailey, J. T. Baines, C. Bakalis, O. K. Baker, P. J. Bakker, E. Bakos, D. Bakshi Gupta, S. Balaji, R. Balasubramanian, E. M. Baldin, P. Balek, E. Ballabene, F. Balli, L. M. Baltes, W. K. Balunas, J. Balz, E. Banas, M. Bandieramonte, A. Bandyopadhyay, S. Bansal, L. Barak, E. L. Barberio, D. Barberis, M. Barbero, G. Barbour, K. N. Barends, T. Barillari, M.-S. Barisits, J. Barkeloo, T. Barklow, R. M. Barnett, P. Baron, D. A. Baron Moreno, A. Baroncelli, G. Barone, A. J. Barr, L. Barranco Navarro, F. Barreiro, J. Barreiro Guimarães da Costa, U. Barron, M. G. Barros Teixeira, S. Barsov, F. Bartels, R. Bartoldus, A. E. Barton, P. Bartos, A. Basalaev, A. Basan, M. Baselga, I. Bashta, A. Bassalat, M. J. Basso, C. R. Basson, R. L. Bates, S. Batlamous, J. R. Batley, B. Batool, M. Battaglia, M. Bauce, P. Bauer, A. Bayirli, J. B. Beacham, T. Beau, P. H. Beauchemin, F. Becherer, P. Bechtle, H. P. Beck, K. Becker, C. Becot, A. J. Beddall, V. A. Bednyakov, C. P. Bee, L. J. Beemster, T. A. Beermann, M. Begalli, M. Begel, A. Behera, J. K. Behr, C. Beirao Da Cruz E Silva, J. F. Beirer, F. Beisiegel, M. Belfkir, G. Bella, L. Bellagamba, A. Bellerive, P. Bellos, K. Beloborodov, K. Belotskiy, N. L. Belyaev, D. Benchekroun, F. Bendebba, Y. Benhammou, D. P. Benjamin, M. Benoit, J. R. Bensinger, S. Bentvelsen, L. Beresford, M. Beretta, D. Berge, E. Bergeaas Kuutmann, N. Berger, B. Bergmann, J. Beringer, S. Berlendis, G. Bernardi, C. Bernius, F. U. Bernlochner, T. Berry, P. Berta, A. Berthold, I. A. Bertram, O. Bessidskaia Bylund, S. Bethke, A. Betti, A. J. Bevan, M. Bhamjee, S. Bhatta, D. S. Bhattacharya, P. Bhattarai, V. S. Bhopatkar, R. Bi, R. Bi, R. M. Bianchi, O. Biebel, R. Bielski, M. Biglietti, T. R. V. Billoud, M. Bindi, A. Bingul, C. Bini, S. Biondi, A. Biondini, C. J. Birch-sykes, G. A. Bird, M. Birman, T. Bisanz, D. Biswas, A. Bitadze, K. Bjørke, I. Bloch, C. Blocker, A. Blue, U. Blumenschein, J. Blumenthal, G. J. Bobbink, V. S. Bobrovnikov, M. Boehler, D. Bogavac, A. G. Bogdanchikov, C. Bohm, V. Boisvert, P. Bokan, T. Bold, M. Bomben, M. Bona, M. Boonekamp, C. D. Booth, A. G. Borbély, H. M. Borecka-Bielska, L. S. Borgna, G. Borissov, D. Bortoletto, D. Boscherini, M. Bosman, J. D. Bossio Sola, K. Bouaouda, J. Boudreau, E. V. Bouhova-Thacker, D. Boumediene, R. Bouquet, A. Boveia, J. Boyd, D. Boye, I. R. Boyko, J. Bracinik, N. Brahimi, G. Brandt, O. Brandt, F. Braren, B. Brau, J. E. Brau, W. D. Breaden Madden, K. Brendlinger, R. Brener, L. Brenner, R. Brenner, S. Bressler, B. Brickwedde, D. Britton, D. Britzger, I. Brock, G. Brooijmans, W. K. Brooks, E. Brost, P. A. Bruckman de Renstrom, B. Brüers, D. Bruncko, A. Bruni, G. Bruni, M. Bruschi, N. Bruscino, L. Bryngemark, T. Buanes, Q. Buat, P. Buchholz, A. G. Buckley, I. A. Budagov, M. K. Bugge, O. Bulekov, B. A. Bullard, S. Burdin, C. D. Burgard, A. M. Burger, B. Burghgrave, J. T. P. Burr, C. D. Burton, J. C. Burzynski, E. L. Busch, V. Büscher, P. J. Bussey, J. M. Butler, C. M. Buttar, J. M. Butterworth, W. Buttinger, C. J. Buxo Vazquez, A. R. Buzykaev, G. Cabras, S. Cabrera Urbán, D. Caforio, H. Cai, Y. Cai, V. M. M. Cairo, O. Cakir, N. Calace, P. Calafiura, G. Calderini, P. Calfayan, G. Callea, L. P. Caloba, D. Calvet, S. Calvet, T. P. Calvet, M. Calvetti, R. Camacho Toro, S. Camarda, D. Camarero Munoz, P. Camarri, M. T. Camerlingo, D. Cameron, C. Camincher, M. Campanelli, A. Camplani, V. Canale, A. Canesse, M. Cano Bret, J. Cantero, Y. Cao, F. Capocasa, M. Capua, A. Carbone, R. Cardarelli, J. C. J. Cardenas, F. Cardillo, T. Carli, G. Carlino, B. T. Carlson, E. M. Carlson, L. Carminati, M. Carnesale, S. Caron, E. Carquin, S. Carrá, G. Carratta, F. Carrio Argos, J. W. S. Carter, T. M. Carter, M. P. Casado, A. F. Casha, E. G. Castiglia, F. L. Castillo, L. Castillo Garcia, V. Castillo Gimenez, N. F. Castro, A. Catinaccio, J. R. Catmore, V. Cavaliere, N. Cavalli, V. Cavasinni, E. Celebi, F. Celli, M. S. Centonze, K. Cerny, A. S. Cerqueira, A. Cerri, L. Cerrito, F. Cerutti, A. Cervelli, S. A. Cetin, Z. Chadi, D. Chakraborty, M. Chala, J. Chan, W. S. Chan, W. Y. Chan, J. D. Chapman, B. Chargeishvili, D. G. Charlton, T. P. Charman, M. Chatterjee, S. Chekanov, S. V. Chekulaev, G. A. Chelkov, A. Chen, B. Chen, B. Chen, C. Chen, H. Chen, H. Chen, J. Chen, J. Chen, S. Chen, S. J. Chen, X. Chen, X. Chen, Y. Chen, C. L. Cheng, H. C. Cheng, A. Cheplakov, E. Cheremushkina, E. Cherepanova, R. Cherkaoui El Moursli, E. Cheu, K. Cheung, L. Chevalier, V. Chiarella, G. Chiarelli, G. Chiodini, A. S. Chisholm, A. Chitan, Y. H. Chiu, M. V. Chizhov, K. Choi, A. R. Chomont, Y. Chou, E. Y. S. Chow, T. Chowdhury, L. D. Christopher, K. L. Chu, M. C. Chu, X. Chu, J. Chudoba, J. J. Chwastowski, D. Cieri, K. M. Ciesla, V. Cindro, A. Ciocio, F. Cirotto, Z. H. Citron, M. Citterio, D. A. Ciubotaru, B. M. Ciungu, A. Clark, P. J. Clark, J. M. Clavijo Columbie, S. E. Clawson, C. Clement, J. Clercx, L. Clissa, Y. Coadou, M. Cobal, A. Coccaro, R. F. Coelho Barrue, R. Coelho Lopes De Sa, S. Coelli, H. Cohen, A. E. C. Coimbra, B. Cole, J. Collot, P. Conde Muiño, M. P. Connell, S. H. Connell, I. A. Connelly, E. I. Conroy, F. Conventi, H. G. Cooke, A. M. Cooper-Sarkar, F. Cormier, L. D. Corpe, M. Corradi, E. E. Corrigan, F. Corriveau, A. Cortes-Gonzalez, M. J. Costa, F. Costanza, D. Costanzo, B. M. Cote, G. Cowan, J. W. Cowley, K. Cranmer, S. Crépé-Renaudin, F. Crescioli, M. Cristinziani, M. Cristoforetti, V. Croft, G. Crosetti, A. Cueto, T. Cuhadar Donszelmann, H. Cui, Z. Cui, A. R. Cukierman, W. R. Cunningham, F. Curcio, P. Czodrowski, M. M. Czurylo, M. J. Da Cunha Sargedas De Sousa, J. V. Da Fonseca Pinto, C. Da Via, W. Dabrowski, T. Dado, S. Dahbi, T. Dai, C. Dallapiccola, M. Dam, G. D’amen, V. D’Amico, J. Damp, J. R. Dandoy, M. F. Daneri, M. Danninger, V. Dao, G. Darbo, S. Darmora, S. J. Das, A. Dattagupta, S. D’Auria, C. David, T. Davidek, D. R. Davis, B. Davis-Purcell, I. Dawson, K. De, R. De Asmundis, M. De Beurs, S. De Castro, N. De Groot, P. de Jong, H. De la Torre, A. De Maria, A. De Salvo, U. De Sanctis, A. De Santo, J. B. De Vivie De Regie, D. V. Dedovich, J. Degens, A. M. Deiana, F. Del Corso, J. Del Peso, F. Del Rio, F. Deliot, C. M. Delitzsch, M. Della Pietra, D. Della Volpe, A. Dell’Acqua, L. Dell’Asta, M. Delmastro, P. A. Delsart, S. Demers, M. Demichev, S. P. Denisov, L. D’Eramo, D. Derendarz, F. Derue, P. Dervan, K. Desch, K. Dette, C. Deutsch, P. O. Deviveiros, F. A. Di Bello, A. Di Ciaccio, L. Di Ciaccio, A. Di Domenico, C. Di Donato, A. Di Girolamo, G. Di Gregorio, A. Di Luca, B. Di Micco, R. Di Nardo, C. Diaconu, F. A. Dias, T. Dias Do Vale, M. A. Diaz, F. G. Diaz Capriles, M. Didenko, E. B. Diehl, L. Diehl, S. Díez Cornell, C. Diez Pardos, C. Dimitriadi, A. Dimitrievska, W. Ding, J. Dingfelder, I.-M. Dinu, S. J. Dittmeier, F. Dittus, F. Djama, T. Djobava, J. I. Djuvsland, D. Dodsworth, C. Doglioni, J. Dolejsi, Z. Dolezal, M. Donadelli, B. Dong, J. Donini, A. D’Onofrio, M. D’Onofrio, J. Dopke, A. Doria, M. T. Dova, A. T. Doyle, M. A. Draguet, E. Drechsler, E. Dreyer, I. Drivas-koulouris, A. S. Drobac, D. Du, T. A. du Pree, F. Dubinin, M. Dubovsky, E. Duchovni, G. Duckeck, O. A. Ducu, D. Duda, A. Dudarev, M. D’uffizi, L. Duflot, M. Dührssen, C. Dülsen, A. E. Dumitriu, M. Dunford, S. Dungs, K. Dunne, A. Duperrin, H. Duran Yildiz, M. Düren, A. Durglishvili, B. L. Dwyer, G. I. Dyckes, M. Dyndal, S. Dysch, B. S. Dziedzic, Z. O. Earnshaw, B. Eckerova, M. G. Eggleston, E. Egidio Purcino De Souza, L. F. Ehrke, G. Eigen, K. Einsweiler, T. Ekelof, P. A. Ekman, Y. El Ghazali, H. El Jarrari, A. El Moussaouy, V. Ellajosyula, M. Ellert, F. Ellinghaus, A. A. Elliot, N. Ellis, J. Elmsheuser, M. Elsing, D. Emeliyanov, A. Emerman, Y. Enari, I. Ene, S. Epari, J. Erdmann, A. Ereditato, P. A. Erland, M. Errenst, M. Escalier, C. Escobar, E. Etzion, G. Evans, H. Evans, M. O. Evans, A. Ezhilov, S. Ezzarqtouni, F. Fabbri, L. Fabbri, G. Facini, V. Fadeyev, R. M. Fakhrutdinov, S. Falciano, P. J. Falke, S. Falke, J. Faltova, Y. Fan, Y. Fang, G. Fanourakis, M. Fanti, M. Faraj, A. Farbin, A. Farilla, T. Farooque, S. M. Farrington, F. Fassi, D. Fassouliotis, M. Faucci Giannelli, W. J. Fawcett, L. Fayard, O. L. Fedin, G. Fedotov, M. Feickert, L. Feligioni, A. Fell, D. E. Fellers, C. Feng, M. Feng, M. J. Fenton, A. B. Fenyuk, L. Ferencz, S. W. Ferguson, J. Pretel, J. Ferrando, A. Ferrari, P. Ferrari, R. Ferrari, D. Ferrere, C. Ferretti, F. Fiedler, A. Filipčič, E. K. Filmer, F. Filthaut, M. C. N. Fiolhais, L. Fiorini, F. Fischer, W. C. Fisher, T. Fitschen, I. Fleck, P. Fleischmann, T. Flick, L. Flores, M. Flores, L. R. Flores Castillo, F. M. Follega, N. Fomin, J. H. Foo, B. C. Forland, A. Formica, A. C. Forti, E. Fortin, A. W. Fortman, M. G. Foti, L. Fountas, D. Fournier, H. Fox, P. Francavilla, S. Francescato, M. Franchini, S. Franchino, D. Francis, L. Franco, L. Franconi, M. Franklin, G. Frattari, A. C. Freegard, P. M. Freeman, W. S. Freund, N. Fritzsche, A. Froch, D. Froidevaux, J. A. Frost, Y. Fu, M. Fujimoto, E. Fullana Torregrosa, J. Fuster, A. Gabrielli, A. Gabrielli, P. Gadow, G. Gagliardi, L. G. Gagnon, G. E. Gallardo, E. J. Gallas, B. J. Gallop, R. Gamboa Goni, K. K. Gan, S. Ganguly, J. Gao, Y. Gao, F. M. Garay Walls, B. Garcia, C. García, J. E. García Navarro, J. A. García Pascual, M. Garcia-Sciveres, R. W. Gardner, D. Garg, R. B. Garg, S. Gargiulo, C. A. Garner, V. Garonne, S. J. Gasiorowski, P. Gaspar, G. Gaudio, V. Gautam, P. Gauzzi, I. L. Gavrilenko, A. Gavrilyuk, C. Gay, G. Gaycken, E. N. Gazis, A. A. Geanta, C. M. Gee, J. Geisen, M. Geisen, C. Gemme, M. H. Genest, S. Gentile, S. George, W. F. George, T. Geralis, L. O. Gerlach, P. Gessinger-Befurt, M. Ghasemi Bostanabad, M. Ghneimat, A. Ghosal, A. Ghosh, A. Ghosh, B. Giacobbe, S. Giagu, N. Giangiacomi, P. Giannetti, A. Giannini, S. M. Gibson, M. Gignac, D. T. Gil, A. K. Gilbert, B. J. Gilbert, D. Gillberg, G. Gilles, N. E. K. Gillwald, L. Ginabat, D. M. Gingrich, M. P. Giordani, P. F. Giraud, G. Giugliarelli, D. Giugni, F. Giuli, I. Gkialas, L. K. Gladilin, C. Glasman, G. R. Gledhill, M. Glisic, I. Gnesi, Y. Go, M. Goblirsch-Kolb, D. Godin, S. Goldfarb, T. Golling, M. G. D. Gololo, D. Golubkov, J. P. Gombas, A. Gomes, G. Gomes Da Silva, A. J. Gomez Delegido, R. Goncalves Gama, R. Gonçalo, G. Gonella, L. Gonella, A. Gongadze, F. Gonnella, J. L. Gonski, S. González de la Hoz, S. Gonzalez Fernandez, R. Gonzalez Lopez, C. Gonzalez Renteria, R. Gonzalez Suarez, S. Gonzalez-Sevilla, G. R. Gonzalvo Rodriguez, R. Y. González Andana, L. Goossens, N. A. Gorasia, P. A. Gorbounov, B. Gorini, E. Gorini, A. Gorišek, A. T. Goshaw, M. I. Gostkin, C. A. Gottardo, M. Gouighri, V. Goumarre, A. G. Goussiou, N. Govender, C. Goy, I. Grabowska-Bold, K. Graham, E. Gramstad, S. Grancagnolo, M. Grandi, V. Gratchev, P. M. Gravila, F. G. Gravili, H. M. Gray, M. Greco, C. Grefe, I. M. Gregor, P. Grenier, C. Grieco, A. A. Grillo, K. Grimm, S. Grinstein, J.-F. Grivaz, E. Gross, J. Grosse-Knetter, C. Grud, A. Grummer, J. C. Grundy, L. Guan, W. Guan, C. Gubbels, J. G. R. Guerrero Rojas, G. Guerrieri, F. Guescini, R. Gugel, J. A. M. Guhit, A. Guida, T. Guillemin, E. Guilloton, S. Guindon, F. Guo, J. Guo, L. Guo, Y. Guo, R. Gupta, S. Gurbuz, S. S. Gurdasani, G. Gustavino, M. Guth, P. Gutierrez, L. F. Gutierrez Zagazeta, C. Gutschow, C. Guyot, C. Gwenlan, C. B. Gwilliam, E. S. Haaland, A. Haas, M. Habedank, C. Haber, H. K. Hadavand, A. Hadef, S. Hadzic, M. Haleem, J. Haley, J. J. Hall, G. D. Hallewell, L. Halser, K. Hamano, H. Hamdaoui, M. Hamer, G. N. Hamity, J. Han, K. Han, L. Han, L. Han, S. Han, Y. F. Han, K. Hanagaki, M. Hance, D. A. Hangal, M. D. Hank, R. Hankache, J. B. Hansen, J. D. Hansen, P. H. Hansen, K. Hara, D. Harada, T. Harenberg, S. Harkusha, Y. T. Harris, N. M. Harrison, P. F. Harrison, N. M. Hartman, N. M. Hartmann, Y. Hasegawa, A. Hasib, S. Haug, R. Hauser, M. Havranek, C. M. Hawkes, R. J. Hawkings, S. Hayashida, D. Hayden, C. Hayes, R. L. Hayes, C. P. Hays, J. M. Hays, H. S. Hayward, F. He, Y. He, Y. He, M. P. Heath, V. Hedberg, A. L. Heggelund, N. D. Hehir, C. Heidegger, K. K. Heidegger, W. D. Heidorn, J. Heilman, S. Heim, T. Heim, J. G. Heinlein, J. J. Heinrich, L. Heinrich, J. Hejbal, L. Helary, A. Held, S. Hellesund, C. M. Helling, S. Hellman, C. Helsens, R. C. W. Henderson, L. Henkelmann, A. M. Henriques Correia, H. Herde, Y. Hernández Jiménez, H. Herr, M. G. Herrmann, T. Herrmann, G. Herten, R. Hertenberger, L. Hervas, N. P. Hessey, H. Hibi, E. Higón-Rodriguez, S. J. Hillier, I. Hinchliffe, F. Hinterkeuser, M. Hirose, S. Hirose, D. Hirschbuehl, T. G. Hitchings, B. Hiti, J. Hobbs, R. Hobincu, N. Hod, M. C. Hodgkinson, B. H. Hodkinson, A. Hoecker, J. Hofer, D. Hohn, T. Holm, M. Holzbock, L. B. A. H. Hommels, B. P. Honan, J. Hong, T. M. Hong, Y. Hong, J. C. Honig, A. Hönle, B. H. Hooberman, W. H. Hopkins, Y. Horii, S. Hou, A. S. Howard, J. Howarth, J. Hoya, M. Hrabovsky, A. Hrynevich, T. Hryn’ova, P. J. Hsu, S.-C. Hsu, Q. Hu, Y. F. Hu, D. P. Huang, S. Huang, X. Huang, Y. Huang, Y. Huang, Z. Huang, Z. Hubacek, M. Huebner, F. Huegging, T. B. Huffman, M. Huhtinen, S. K. Huiberts, R. Hulsken, N. Huseynov, J. Huston, J. Huth, R. Hyneman, S. Hyrych, G. Iacobucci, G. Iakovidis, I. Ibragimov, L. Iconomidou-Fayard, P. Iengo, R. Iguchi, T. Iizawa, Y. Ikegami, A. Ilg, N. Ilic, H. Imam, T. Ingebretsen Carlson, G. Introzzi, M. Iodice, V. Ippolito, M. Ishino, W. Islam, C. Issever, S. Istin, H. Ito, J. M. Iturbe Ponce, R. Iuppa, A. Ivina, J. M. Izen, V. Izzo, P. Jacka, P. Jackson, R. M. Jacobs, B. P. Jaeger, C. S. Jagfeld, G. Jäkel, K. Jakobs, T. Jakoubek, J. Jamieson, K. W. Janas, G. Jarlskog, A. E. Jaspan, T. Javůrek, M. Javurkova, F. Jeanneau, L. Jeanty, J. Jejelava, P. Jenni, C. E. Jessiman, S. Jézéquel, J. Jia, X. Jia, X. Jia, Z. Jia, Y. Jiang, S. Jiggins, J. Jimenez Pena, S. Jin, A. Jinaru, O. Jinnouchi, H. Jivan, P. Johansson, K. A. Johns, C. A. Johnson, D. M. Jones, E. Jones, P. Jones, R. W. L. Jones, T. J. Jones, J. Jovicevic, X. Ju, J. J. Junggeburth, A. Juste Rozas, S. Kabana, A. Kaczmarska, M. Kado, H. Kagan, M. Kagan, A. Kahn, A. Kahn, C. Kahra, T. Kaji, E. Kajomovitz, N. Kakati, C. W. Kalderon, A. Kamenshchikov, N. J. Kang, Y. Kano, D. Kar, K. Karava, M. J. Kareem, E. Karentzos, I. Karkanias, S. N. Karpov, Z. M. Karpova, V. Kartvelishvili, A. N. Karyukhin, E. Kasimi, C. Kato, J. Katzy, S. Kaur, K. Kawade, K. Kawagoe, T. Kawaguchi, T. Kawamoto, G. Kawamura, E. F. Kay, F. I. Kaya, S. Kazakos, V. F. Kazanin, Y. Ke, J. M. Keaveney, R. Keeler, G. V. Kehris, J. S. Keller, A. S. Kelly, D. Kelsey, J. J. Kempster, J. Kendrick, K. E. Kennedy, O. Kepka, B. P. Kerridge, S. Kersten, B. P. Kerševan, L. Keszeghova, S. Ketabchi Haghighat, M. Khandoga, A. Khanov, A. G. Kharlamov, T. Kharlamova, E. E. Khoda, T. J. Khoo, G. Khoriauli, J. Khubua, Y. A. R. Khwaira, M. Kiehn, A. Kilgallon, D. W. Kim, E. Kim, Y. K. Kim, N. Kimura, A. Kirchhoff, D. Kirchmeier, C. Kirfel, J. Kirk, A. E. Kiryunin, T. Kishimoto, D. P. Kisliuk, C. Kitsaki, O. Kivernyk, M. Klassen, C. Klein, L. Klein, M. H. Klein, M. Klein, U. Klein, P. Klimek, A. Klimentov, F. Klimpel, T. Klingl, T. Klioutchnikova, F. F. Klitzner, P. Kluit, S. Kluth, E. Kneringer, T. M. Knight, A. Knue, D. Kobayashi, R. Kobayashi, M. Kocian, T. Kodama, P. Kodyš, D. M. Koeck, P. T. Koenig, T. Koffas, N. M. Köhler, M. Kolb, I. Koletsou, T. Komarek, K. Köneke, A. X. Y. Kong, T. Kono, N. Konstantinidis, B. Konya, R. Kopeliansky, S. Koperny, K. Korcyl, K. Kordas, G. Koren, A. Korn, S. Korn, I. Korolkov, N. Korotkova, B. Kortman, O. Kortner, S. Kortner, W. H. Kostecka, V. V. Kostyukhin, A. Kotsokechagia, A. Kotwal, A. Koulouris, A. Kourkoumeli-Charalampidi, C. Kourkoumelis, E. Kourlitis, O. Kovanda, R. Kowalewski, W. Kozanecki, A. S. Kozhin, V. A. Kramarenko, G. Kramberger, P. Kramer, M. W. Krasny, A. Krasznahorkay, J. A. Kremer, T. Kresse, J. Kretzschmar, K. Kreul, P. Krieger, F. Krieter, S. Krishnamurthy, A. Krishnan, M. Krivos, K. Krizka, K. Kroeninger, H. Kroha, J. Kroll, J. Kroll, K. S. Krowpman, U. Kruchonak, H. Krüger, N. Krumnack, M. C. Kruse, J. A. Krzysiak, A. Kubota, O. Kuchinskaia, S. Kuday, D. Kuechler, J. T. Kuechler, S. Kuehn, T. Kuhl, V. Kukhtin, Y. Kulchitsky, S. Kuleshov, M. Kumar, N. Kumari, M. Kuna, A. Kupco, T. Kupfer, A. Kupich, O. Kuprash, H. Kurashige, L. L. Kurchaninov, Y. A. Kurochkin, A. Kurova, E. S. Kuwertz, M. Kuze, A. K. Kvam, J. Kvita, T. Kwan, K. W. Kwok, C. Lacasta, F. Lacava, H. Lacker, D. Lacour, N. N. Lad, E. Ladygin, B. Laforge, T. Lagouri, S. Lai, I. K. Lakomiec, N. Lalloue, J. E. Lambert, S. Lammers, W. Lampl, C. Lampoudis, A. N. Lancaster, E. Lançon, U. Landgraf, M. P. J. Landon, V. S. Lang, R. J. Langenberg, A. J. Lankford, F. Lanni, K. Lantzsch, A. Lanza, A. Lapertosa, J. F. Laporte, T. Lari, F. Lasagni Manghi, M. Lassnig, V. Latonova, T. S. Lau, A. Laudrain, A. Laurier, S. D. Lawlor, Z. Lawrence, M. Lazzaroni, B. Le, B. Leban, A. Lebedev, M. LeBlanc, T. LeCompte, F. Ledroit-Guillon, A. C. A. Lee, G. R. Lee, L. Lee, S. C. Lee, S. Lee, L. L. Leeuw, H. P. Lefebvre, M. Lefebvre, C. Leggett, K. Lehmann, G. Lehmann Miotto, W. A. Leight, A. Leisos, M. A. L. Leite, C. E. Leitgeb, R. Leitner, K. J. C. Leney, T. Lenz, S. Leone, C. Leonidopoulos, A. Leopold, C. Leroy, R. Les, C. G. Lester, M. Levchenko, J. Levêque, D. Levin, L. J. Levinson, M. P. Lewicki, D. J. Lewis, B. Li, B. Li, C. Li, C.-Q. Li, H. Li, H. Li, H. Li, J. Li, K. Li, L. Li, M. Li, Q. Y. Li, S. Li, T. Li, X. Li, Z. Li, Z. Li, Z. Li, Z. Li, Z. Liang, M. Liberatore, B. Liberti, K. Lie, J. Lieber Marin, K. Lin, R. A. Linck, R. E. Lindley, J. H. Lindon, A. Linss, E. Lipeles, A. Lipniacka, T. M. Liss, A. Lister, J. D. Little, B. Liu, B. X. Liu, D. Liu, J. B. Liu, J. K. K. Liu, K. Liu, M. Liu, M. Y. Liu, P. Liu, Q. Liu, X. Liu, Y. Liu, Y. Liu, Y. L. Liu, Y. W. Liu, M. Livan, J. Llorente Merino, S. L. Lloyd, E. M. Lobodzinska, P. Loch, S. Loffredo, T. Lohse, K. Lohwasser, M. Lokajicek, J. D. Long, I. Longarini, L. Longo, R. Longo, I. Lopez Paz, A. Lopez Solis, J. Lorenz, N. Lorenzo Martinez, A. M. Lory, A. Lösle, X. Lou, X. Lou, A. Lounis, J. Love, P. A. Love, J. J. Lozano Bahilo, G. Lu, M. Lu, S. Lu, Y. J. Lu, H. J. Lubatti, C. Luci, F. L. Lucio Alves, A. Lucotte, F. Luehring, I. Luise, O. Lukianchuk, O. Lundberg, B. Lund-Jensen, N. A. Luongo, M. S. Lutz, D. Lynn, H. Lyons, R. Lysak, E. Lytken, F. Lyu, V. Lyubushkin, T. Lyubushkina, H. Ma, L. L. Ma, Y. Ma, D. M. Mac Donell, G. Maccarrone, J. C. MacDonald, R. Madar, W. F. Mader, J. Maeda, T. Maeno, M. Maerker, V. Magerl, J. Magro, H. Maguire, D. J. Mahon, C. Maidantchik, A. Maio, K. Maj, O. Majersky, S. Majewski, N. Makovec, V. Maksimovic, B. Malaescu, Pa. Malecki, V. P. Maleev, F. Malek, D. Malito, U. Mallik, C. Malone, S. Maltezos, S. Malyukov, J. Mamuzic, G. Mancini, G. Manco, J. P. Mandalia, I. Mandić, L. Manhaes de Andrade Filho, I. M. Maniatis, M. Manisha, J. Manjarres Ramos, D. C. Mankad, K. H. Mankinen, A. Mann, A. Manousos, B. Mansoulie, S. Manzoni, A. Marantis, G. Marchiori, M. Marcisovsky, L. Marcoccia, C. Marcon, M. Marinescu, M. Marjanovic, Z. Marshall, S. Marti-Garcia, T. A. Martin, V. J. Martin, B. Martin dit Latour, L. Martinelli, M. Martinez, P. Martinez Agullo, V. I. Martinez Outschoorn, P. Martinez Suarez, S. Martin-Haugh, V. S. Martoiu, A. C. Martyniuk, A. Marzin, S. R. Maschek, L. Masetti, T. Mashimo, J. Masik, A. L. Maslennikov, L. Massa, P. Massarotti, P. Mastrandrea, A. Mastroberardino, T. Masubuchi, T. Mathisen, A. Matic, N. Matsuzawa, J. Maurer, B. Maček, D. A. Maximov, R. Mazini, I. Maznas, M. Mazza, S. M. Mazza, C. Mc Ginn, J. P. Mc Gowan, S. P. Mc Kee, T. G. McCarthy, W. P. McCormack, E. F. McDonald, A. E. McDougall, J. A. Mcfayden, G. Mchedlidze, R. P. Mckenzie, T. C. Mclachlan, D. J. Mclaughlin, K. D. McLean, S. J. McMahon, P. C. McNamara, R. A. McPherson, J. E. Mdhluli, S. Meehan, T. Megy, S. Mehlhase, A. Mehta, B. Meirose, D. Melini, B. R. Mellado Garcia, A. H. Melo, F. Meloni, E. D. Mendes Gouveia, A. M. Mendes Jacques Da Costa, H. Y. Meng, L. Meng, S. Menke, M. Mentink, E. Meoni, C. Merlassino, L. Merola, C. Meroni, G. Merz, O. Meshkov, J. K. R. Meshreki, J. Metcalfe, A. S. Mete, C. Meyer, J.-P. Meyer, M. Michetti, R. P. Middleton, L. Mijović, G. Mikenberg, M. Mikestikova, M. Mikuž, H. Mildner, A. Milic, C. D. Milke, D. W. Miller, L. S. Miller, A. Milov, D. A. Milstead, T. Min, A. A. Minaenko, I. A. Minashvili, L. Mince, A. I. Mincer, B. Mindur, M. Mineev, Y. Minegishi, Y. Mino, L. M. Mir, M. Miralles Lopez, M. Mironova, T. Mitani, A. Mitra, V. A. Mitsou, O. Miu, P. S. Miyagawa, Y. Miyazaki, A. Mizukami, J. U. Mjörnmark, T. Mkrtchyan, M. Mlynarikova, T. Moa, S. Mobius, K. Mochizuki, P. Moder, P. Mogg, A. F. Mohammed, S. Mohapatra, G. Mokgatitswane, B. Mondal, S. Mondal, K. Mönig, E. Monnier, L. Monsonis Romero, J. Montejo Berlingen, M. Montella, F. Monticelli, N. Morange, A. L. Moreira De Carvalho, M. Moreno Llácer, C. Moreno Martinez, P. Morettini, S. Morgenstern, M. Morii, M. Morinaga, V. Morisbak, A. K. Morley, F. Morodei, L. Morvaj, P. Moschovakos, B. Moser, M. Mosidze, T. Moskalets, P. Moskvitina, J. Moss, E. J. W. Moyse, S. Muanza, J. Mueller, D. Muenstermann, R. Müller, G. A. Mullier, J. J. Mullin, D. P. Mungo, J. L. Munoz Martinez, D. Munoz Perez, F. J. Munoz Sanchez, M. Murin, W. J. Murray, A. Murrone, J. M. Muse, M. Muškinja, C. Mwewa, A. G. Myagkov, A. J. Myers, A. A. Myers, G. Myers, M. Myska, B. P. Nachman, O. Nackenhorst, A. Nag, K. Nagai, K. Nagano, J. L. Nagle, E. Nagy, A. M. Nairz, Y. Nakahama, K. Nakamura, H. Nanjo, R. Narayan, E. A. Narayanan, I. Naryshkin, M. Naseri, C. Nass, G. Navarro, J. Navarro-Gonzalez, R. Nayak, P. Y. Nechaeva, F. Nechansky, T. J. Neep, A. Negri, M. Negrini, C. Nellist, C. Nelson, K. Nelson, S. Nemecek, M. Nessi, M. S. Neubauer, F. Neuhaus, J. Neundorf, R. Newhouse, P. R. Newman, C. W. Ng, Y. S. Ng, Y. W. Y. Ng, B. Ngair, H. D. N. Nguyen, R. B. Nickerson, R. Nicolaidou, J. Nielsen, M. Niemeyer, N. Nikiforou, V. Nikolaenko, I. Nikolic-Audit, K. Nikolopoulos, P. Nilsson, H. R. Nindhito, A. Nisati, N. Nishu, R. Nisius, J.-E. Nitschke, E. K. Nkadimeng, S. J. Noacco Rosende, T. Nobe, D. L. Noel, Y. Noguchi, T. Nommensen, M. A. Nomura, M. B. Norfolk, R. R. B. Norisam, B. J. Norman, J. Novak, T. Novak, O. Novgorodova, L. Novotny, R. Novotny, L. Nozka, K. Ntekas, E. Nurse, F. G. Oakham, J. Ocariz, A. Ochi, I. Ochoa, S. Oerdek, A. Ogrodnik, A. Oh, C. C. Ohm, H. Oide, R. Oishi, M. L. Ojeda, Y. Okazaki, M. W. O’Keefe, Y. Okumura, A. Olariu, L. F. Oleiro Seabra, S. A. Olivares Pino, D. Oliveira Damazio, D. Oliveira Goncalves, J. L. Oliver, M. J. R. Olsson, A. Olszewski, J. Olszowska, Ö. O. Öncel, D. C. O’Neil, A. P. O’Neill, A. Onofre, P. U. E. Onyisi, M. J. Oreglia, G. E. Orellana, D. Orestano, N. Orlando, R. S. Orr, V. O’Shea, R. Ospanov, G. Otero y Garzon, H. Otono, P. S. Ott, G. J. Ottino, M. Ouchrif, J. Ouellette, F. Ould-Saada, M. Owen, R. E. Owen, K. Y. Oyulmaz, V. E. Ozcan, N. Ozturk, S. Ozturk, J. Pacalt, H. A. Pacey, K. Pachal, A. Pacheco Pages, C. Padilla Aranda, G. Padovano, S. Pagan Griso, G. Palacino, A. Palazzo, S. Palazzo, S. Palestini, M. Palka, J. Pan, T. Pan, D. K. Panchal, C. E. Pandini, J. G. Panduro Vazquez, H. Pang, P. Pani, G. Panizzo, L. Paolozzi, C. Papadatos, S. Parajuli, A. Paramonov, C. Paraskevopoulos, D. Paredes Hernandez, T. H. Park, M. A. Parker, F. Parodi, E. W. Parrish, V. A. Parrish, J. A. Parsons, U. Parzefall, B. Pascual Dias, L. Pascual Dominguez, V. R. Pascuzzi, F. Pasquali, E. Pasqualucci, S. Passaggio, F. Pastore, P. Pasuwan, J. R. Pater, J. Patton, T. Pauly, J. Pearkes, M. Pedersen, R. Pedro, S. V. Peleganchuk, O. Penc, C. Peng, H. Peng, K. E. Penski, M. Penzin, B. S. Peralva, A. P. Pereira Peixoto, L. Pereira Sanchez, D. V. Perepelitsa, E. Perez Codina, M. Perganti, L. Perini, H. Pernegger, S. Perrella, A. Perrevoort, O. Perrin, K. Peters, R. F. Y. Peters, B. A. Petersen, T. C. Petersen, E. Petit, V. Petousis, C. Petridou, A. Petrukhin, M. Pettee, N. E. Pettersson, A. Petukhov, K. Petukhova, A. Peyaud, R. Pezoa, L. Pezzotti, G. Pezzullo, T. Pham, P. W. Phillips, M. W. Phipps, G. Piacquadio, E. Pianori, F. Piazza, R. Piegaia, D. Pietreanu, A. D. Pilkington, M. Pinamonti, J. L. Pinfold, B. C. Pinheiro Pereira, C. Pitman Donaldson, D. A. Pizzi, L. Pizzimento, A. Pizzini, M.-A. Pleier, V. Plesanovs, V. Pleskot, E. Plotnikova, G. Poddar, R. Poettgen, R. Poggi, L. Poggioli, I. Pogrebnyak, D. Pohl, I. Pokharel, S. Polacek, G. Polesello, A. Poley, R. Polifka, A. Polini, C. S. Pollard, Z. B. Pollock, V. Polychronakos, D. Ponomarenko, L. Pontecorvo, S. Popa, G. A. Popeneciu, D. M. Portillo Quintero, S. Pospisil, P. Postolache, K. Potamianos, I. N. Potrap, C. J. Potter, H. Potti, T. Poulsen, J. Poveda, G. Pownall, M. E. Pozo Astigarraga, A. Prades Ibanez, M. M. Prapa, D. Price, M. Primavera, M. A. Principe Martin, M. L. Proffitt, N. Proklova, K. Prokofiev, G. Proto, S. Protopopescu, J. Proudfoot, M. Przybycien, J. E. Puddefoot, D. Pudzha, P. Puzo, D. Pyatiizbyantseva, J. Qian, Y. Qin, T. Qiu, A. Quadt, M. Queitsch-Maitland, G. Rabanal Bolanos, D. Rafanoharana, F. Ragusa, J. L. Rainbolt, J. A. Raine, S. Rajagopalan, E. Ramakoti, K. Ran, V. Raskina, D. F. Rassloff, S. Rave, B. Ravina, I. Ravinovich, M. Raymond, A. L. Read, N. P. Readioff, D. M. Rebuzzi, G. Redlinger, K. Reeves, J. A. Reidelsturz, D. Reikher, A. Reiss, A. Rej, C. Rembser, A. Renardi, M. Renda, M. B. Rendel, A. G. Rennie, S. Resconi, M. Ressegotti, E. D. Resseguie, S. Rettie, B. Reynolds, E. Reynolds, M. Rezaei Estabragh, O. L. Rezanova, P. Reznicek, E. Ricci, R. Richter, S. Richter, E. Richter-Was, M. Ridel, P. Rieck, P. Riedler, M. Rijssenbeek, A. Rimoldi, M. Rimoldi, L. Rinaldi, T. T. Rinn, M. P. Rinnagel, G. Ripellino, I. Riu, P. Rivadeneira, J. C. Rivera Vergara, F. Rizatdinova, E. Rizvi, C. Rizzi, B. A. Roberts, B. R. Roberts, S. H. Robertson, M. Robin, D. Robinson, C. M. Robles Gajardo, M. Robles Manzano, A. Robson, A. Rocchi, C. Roda, S. Rodriguez Bosca, Y. Rodriguez Garcia, A. Rodriguez Rodriguez, A. M. Rodríguez Vera, S. Roe, J. T. Roemer, A. R. Roepe-Gier, J. Roggel, O. Røhne, R. A. Rojas, B. Roland, C. P. A. Roland, J. Roloff, A. Romaniouk, E. Romano, M. Romano, A. C. Romero Hernandez, N. Rompotis, L. Roos, S. Rosati, B. J. Rosser, E. Rossi, E. Rossi, L. P. Rossi, L. Rossini, R. Rosten, M. Rotaru, B. Rottler, D. Rousseau, D. Rousso, G. Rovelli, A. Roy, A. Rozanov, Y. Rozen, X. Ruan, A. Rubio Jimenez, A. J. Ruby, T. A. Ruggeri, F. Rühr, A. Ruiz-Martinez, A. Rummler, Z. Rurikova, N. A. Rusakovich, H. L. Russell, J. P. Rutherfoord, E. M. Rüttinger, K. Rybacki, M. Rybar, E. B. Rye, A. Ryzhov, J. A. Sabater Iglesias, P. Sabatini, L. Sabetta, H. F.-W. Sadrozinski, F. Safai Tehrani, B. Safarzadeh Samani, M. Safdari, S. Saha, M. Sahinsoy, M. Saimpert, M. Saito, T. Saito, D. Salamani, G. Salamanna, A. Salnikov, J. Salt, A. Salvador Salas, D. Salvatore, F. Salvatore, A. Salzburger, D. Sammel, D. Sampsonidis, D. Sampsonidou, J. Sánchez, A. Sanchez Pineda, V. Sanchez Sebastian, H. Sandaker, C. O. Sander, J. A. Sandesara, M. Sandhoff, C. Sandoval, D. P. C. Sankey, A. Sansoni, L. Santi, C. Santoni, H. Santos, S. N. Santpur, A. Santra, K. A. Saoucha, J. G. Saraiva, J. Sardain, O. Sasaki, K. Sato, C. Sauer, F. Sauerburger, E. Sauvan, P. Savard, R. Sawada, C. Sawyer, L. Sawyer, I. Sayago Galvan, C. Sbarra, A. Sbrizzi, T. Scanlon, J. Schaarschmidt, P. Schacht, D. Schaefer, U. Schäfer, A. C. Schaffer, D. Schaile, R. D. Schamberger, E. Schanet, C. Scharf, V. A. Schegelsky, D. Scheirich, F. Schenck, M. Schernau, C. Scheulen, C. Schiavi, Z. M. Schillaci, E. J. Schioppa, M. Schioppa, B. Schlag, K. E. Schleicher, S. Schlenker, K. Schmieden, C. Schmitt, S. Schmitt, L. Schoeffel, A. Schoening, P. G. Scholer, E. Schopf, M. Schott, J. Schovancova, S. Schramm, F. Schroeder, H.-C. Schultz-Coulon, M. Schumacher, B. A. Schumm, Ph. Schune, A. Schwartzman, T. A. Schwarz, Ph. Schwemling, R. Schwienhorst, A. Sciandra, G. Sciolla, F. Scuri, F. Scutti, C. D. Sebastiani, K. Sedlaczek, P. Seema, S. C. Seidel, A. Seiden, B. D. Seidlitz, T. Seiss, C. Seitz, J. M. Seixas, G. Sekhniaidze, S. J. Sekula, L. Selem, N. Semprini-Cesari, S. Sen, D. Sengupta, V. Senthilkumar, L. Serin, L. Serkin, M. Sessa, H. Severini, S. Sevova, F. Sforza, A. Sfyrla, E. Shabalina, R. Shaheen, J. D. Shahinian, N. W. Shaikh, D. Shaked Renous, L. Y. Shan, M. Shapiro, A. Sharma, A. S. Sharma, P. Sharma, S. Sharma, P. B. Shatalov, K. Shaw, S. M. Shaw, Q. Shen, P. Sherwood, L. Shi, C. O. Shimmin, Y. Shimogama, J. D. Shinner, I. P. J. Shipsey, S. Shirabe, M. Shiyakova, J. Shlomi, M. J. Shochet, J. Shojaii, D. R. Shope, S. Shrestha, E. M. Shrif, M. J. Shroff, P. Sicho, A. M. Sickles, E. Sideras Haddad, O. Sidiropoulou, A. Sidoti, F. Siegert, Dj. Sijacki, R. Sikora, F. Sili, J. M. Silva, M. V. Silva Oliveira, S. B. Silverstein, S. Simion, R. Simoniello, E. L. Simpson, N. D. Simpson, S. Simsek, S. Sindhu, P. Sinervo, V. Sinetckii, S. Singh, S. Singh, S. Sinha, S. Sinha, M. Sioli, I. Siral, S. Yu. Sivoklokov, J. Sjölin, A. Skaf, E. Skorda, P. Skubic, M. Slawinska, V. Smakhtin, B. H. Smart, J. Smiesko, S. Yu. Smirnov, Y. Smirnov, L. N. Smirnova, O. Smirnova, A. C. Smith, E. A. Smith, H. A. Smith, J. L. Smith, R. Smith, M. Smizanska, K. Smolek, A. Smykiewicz, A. A. Snesarev, H. L. Snoek, S. Snyder, R. Sobie, A. Soffer, C. A. Solans Sanchez, E. Yu. Soldatov, U. Soldevila, A. A. Solodkov, S. Solomon, A. Soloshenko, K. Solovieva, O. V. Solovyanov, V. Solovyev, P. Sommer, A. Sonay, W. Y. Song, A. Sopczak, A. L. Sopio, F. Sopkova, V. Sothilingam, S. Sottocornola, R. Soualah, Z. Soumaimi, D. South, S. Spagnolo, M. Spalla, F. Spanò, D. Sperlich, G. Spigo, M. Spina, S. Spinali, D. P. Spiteri, M. Spousta, E. J. Staats, A. Stabile, R. Stamen, M. Stamenkovic, A. Stampekis, M. Standke, E. Stanecka, B. Stanislaus, M. M. Stanitzki, M. Stankaityte, B. Stapf, E. A. Starchenko, G. H. Stark, J. Stark, D. M. Starko, P. Staroba, P. Starovoitov, S. Stärz, R. Staszewski, G. Stavropoulos, J. Steentoft, P. Steinberg, A. L. Steinhebel, B. Stelzer, H. J. Stelzer, O. Stelzer-Chilton, H. Stenzel, T. J. Stevenson, G. A. Stewart, M. C. Stockton, G. Stoicea, M. Stolarski, S. Stonjek, A. Straessner, J. Strandberg, S. Strandberg, M. Strauss, T. Strebler, P. Strizenec, R. Ströhmer, D. M. Strom, L. R. Strom, R. Stroynowski, A. Strubig, S. A. Stucci, B. Stugu, J. Stupak, N. A. Styles, D. Su, S. Su, W. Su, X. Su, K. Sugizaki, V. V. Sulin, M. J. Sullivan, D. M. S. Sultan, L. Sultanaliyeva, S. Sultansoy, T. Sumida, S. Sun, S. Sun, O. Sunneborn Gudnadottir, M. R. Sutton, M. Svatos, M. Swiatlowski, T. Swirski, I. Sykora, M. Sykora, T. Sykora, D. Ta, K. Tackmann, A. Taffard, R. Tafirout, J. S. Tafoya Vargas, R. H. M. Taibah, R. Takashima, K. Takeda, E. P. Takeva, Y. Takubo, M. Talby, A. A. Talyshev, K. C. Tam, N. M. Tamir, A. Tanaka, J. Tanaka, R. Tanaka, M. Tanasini, J. Tang, Z. Tao, S. Tapia Araya, S. Tapprogge, A. Tarek Abouelfadl Mohamed, S. Tarem, K. Tariq, G. Tarna, G. F. Tartarelli, P. Tas, M. Tasevsky, E. Tassi, A. C. Tate, G. Tateno, Y. Tayalati, G. N. Taylor, W. Taylor, H. Teagle, A. S. Tee, R. Teixeira De Lima, P. Teixeira-Dias, J. J. Teoh, K. Terashi, J. Terron, S. Terzo, M. Testa, R. J. Teuscher, A. Thaler, N. Themistokleous, T. Theveneaux-Pelzer, O. Thielmann, D. W. Thomas, J. P. Thomas, E. A. Thompson, P. D. Thompson, E. Thomson, E. J. Thorpe, Y. Tian, V. Tikhomirov, Yu. A. Tikhonov, S. Timoshenko, E. X. L. Ting, P. Tipton, S. Tisserant, S. H. Tlou, A. Tnourji, K. Todome, S. Todorova-Nova, S. Todt, M. Togawa, J. Tojo, S. Tokár, K. Tokushuku, R. Tombs, M. Tomoto, L. Tompkins, P. Tornambe, E. Torrence, H. Torres, E. Torró Pastor, M. Toscani, C. Tosciri, D. R. Tovey, A. Traeet, I. S. Trandafir, T. Trefzger, A. Tricoli, I. M. Trigger, S. Trincaz-Duvoid, D. A. Trischuk, B. Trocmé, A. Trofymov, C. Troncon, L. Truong, M. Trzebinski, A. Trzupek, F. Tsai, M. Tsai, A. Tsiamis, P. V. Tsiareshka, S. Tsigaridas, A. Tsirigotis, V. Tsiskaridze, E. G. Tskhadadze, M. Tsopoulou, Y. Tsujikawa, I. I. Tsukerman, V. Tsulaia, S. Tsuno, O. Tsur, D. Tsybychev, Y. Tu, A. Tudorache, V. Tudorache, A. N. Tuna, S. Turchikhin, I. Turk Cakir, R. Turra, T. Turtuvshin, P. M. Tuts, S. Tzamarias, P. Tzanis, E. Tzovara, K. Uchida, F. Ukegawa, P. A. Ulloa Poblete, G. Unal, M. Unal, A. Undrus, G. Unel, K. Uno, J. Urban, P. Urquijo, G. Usai, R. Ushioda, M. Usman, Z. Uysal, V. Vacek, B. Vachon, K. O. H. Vadla, T. Vafeiadis, C. Valderanis, E. Valdes Santurio, M. Valente, S. Valentinetti, A. Valero, A. Vallier, J. A. Valls Ferrer, T. R. Van Daalen, P. Van Gemmeren, S. Van Stroud, I. Van Vulpen, M. Vanadia, W. Vandelli, M. Vandenbroucke, E. R. Vandewall, D. Vannicola, L. Vannoli, R. Vari, E. W. Varnes, C. Varni, T. Varol, D. Varouchas, L. Varriale, K. E. Varvell, M. E. Vasile, L. Vaslin, G. A. Vasquez, F. Vazeille, T. Vazquez Schroeder, J. Veatch, V. Vecchio, M. J. Veen, I. Veliscek, L. M. Veloce, F. Veloso, S. Veneziano, A. Ventura, A. Verbytskyi, M. Verducci, C. Vergis, M. Verissimo De Araujo, W. Verkerke, J. C. Vermeulen, C. Vernieri, P. J. Verschuuren, M. Vessella, M. L. Vesterbacka, M. C. Vetterli, A. Vgenopoulos, N. Viaux Maira, T. Vickey, O. E. Vickey Boeriu, G. H. A. Viehhauser, L. Vigani, M. Villa, M. Villaplana Perez, E. M. Villhauer, E. Vilucchi, M. G. Vincter, G. S. Virdee, A. Vishwakarma, C. Vittori, I. Vivarelli, V. Vladimirov, E. Voevodina, F. Vogel, P. Vokac, J. Von Ahnen, E. Von Toerne, B. Vormwald, V. Vorobel, K. Vorobev, M. Vos, J. H. Vossebeld, M. Vozak, L. Vozdecky, N. Vranjes, M. Vranjes Milosavljevic, M. Vreeswijk, R. Vuillermet, O. Vujinovic, I. Vukotic, S. Wada, C. Wagner, W. Wagner, S. Wahdan, H. Wahlberg, R. Wakasa, M. Wakida, V. M. Walbrecht, J. Walder, R. Walker, W. Walkowiak, A. M. Wang, A. Z. Wang, C. Wang, C. Wang, H. Wang, J. Wang, P. Wang, R.-J. Wang, R. Wang, R. Wang, S. M. Wang, S. Wang, T. Wang, W. T. Wang, W. X. Wang, X. Wang, X. Wang, X. Wang, Y. Wang, Y. Wang, Z. Wang, Z. Wang, A. Warburton, R. J. Ward, N. Warrack, A. T. Watson, M. F. Watson, G. Watts, B. M. Waugh, A. F. Webb, C. Weber, M. S. Weber, S. A. Weber, S. M. Weber, C. Wei, Y. Wei, A. R. Weidberg, J. Weingarten, M. Weirich, C. Weiser, C. J. Wells, T. Wenaus, B. Wendland, T. Wengler, N. S. Wenke, N. Wermes, M. Wessels, K. Whalen, A. M. Wharton, A. S. White, A. White, M. J. White, D. Whiteson, L. Wickremasinghe, W. Wiedenmann, C. Wiel, M. Wielers, N. Wieseotte, C. Wiglesworth, L. A. M. Wiik-Fuchs, D. J. Wilbern, H. G. Wilkens, D. M. Williams, H. H. Williams, S. Williams, S. Willocq, P. J. Windischhofer, F. Winklmeier, B. T. Winter, M. Wittgen, M. Wobisch, A. Wolf, R. Wölker, J. Wollrath, M. W. Wolter, H. Wolters, V. W. S. Wong, A. F. Wongel, S. D. Worm, B. K. Wosiek, K. W. Woźniak, K. Wraight, J. Wu, M. Wu, S. L. Wu, X. Wu, Y. Wu, Z. Wu, J. Wuerzinger, T. R. Wyatt, B. M. Wynne, S. Xella, L. Xia, M. Xia, J. Xiang, X. Xiao, M. Xie, X. Xie, J. Xiong, I. Xiotidis, D. Xu, H. Xu, L. Xu, R. Xu, T. Xu, W. Xu, Y. Xu, Z. Xu, Z. Xu, B. Yabsley, S. Yacoob, N. Yamaguchi, Y. Yamaguchi, H. Yamauchi, T. Yamazaki, Y. Yamazaki, J. Yan, S. Yan, Z. Yan, H. J. Yang, H. T. Yang, S. Yang, T. Yang, X. Yang, X. Yang, Y. Yang, Z. Yang, W.-M. Yao, Y. C. Yap, H. Ye, J. Ye, S. Ye, X. Ye, Y. Yeh, I. Yeletskikh, M. R. Yexley, P. Yin, K. Yorita, C. J. S. Young, C. Young, M. Yuan, R. Yuan, L. Yue, X. Yue, M. Zaazoua, B. Zabinski, E. Zaid, T. Zakareishvili, N. Zakharchuk, S. Zambito, J. Zang, D. Zanzi, O. Zaplatilek, S. V. Zeißner, C. Zeitnitz, J. C. Zeng, D. T. Zenger, O. Zenin, T. Ženiš, S. Zenz, S. Zerradi, D. Zerwas, B. Zhang, D. F. Zhang, G. Zhang, J. Zhang, K. Zhang, L. Zhang, R. Zhang, S. Zhang, T. Zhang, X. Zhang, X. Zhang, Z. Zhang, Z. Zhang, H. Zhao, P. Zhao, T. Zhao, Y. Zhao, Z. Zhao, A. Zhemchugov, Z. Zheng, D. Zhong, B. Zhou, C. Zhou, H. Zhou, N. Zhou, Y. Zhou, C. G. Zhu, C. Zhu, H. L. Zhu, H. Zhu, J. Zhu, Y. Zhu, Y. Zhu, X. Zhuang, K. Zhukov, V. Zhulanov, N. I. Zimine, J. Zinsser, M. Ziolkowski, L. Živković, A. Zoccoli, K. Zoch, T. G. Zorbas, O. Zormpa, W. Zou, L. Zwalinski

**Affiliations:** 1 CPPM, Aix-Marseille Université, CNRS/IN2P3, Marseille, France; 2 Homer L. Dodge Department of Physics and Astronomy, University of Oklahoma, Norman, OK United States of America; 3 Department of Physics, University of Massachusetts, Amherst, MA United States of America; 4 II. Physikalisches Institut, Georg-August-Universität Göttingen, Göttingen, Germany; 5 Physics Department, Brookhaven National Laboratory, Upton, NY United States of America; 6 Faculté des sciences, Université Mohammed V, Rabat, Morocco; 7 Physics Department, An-Najah National University, Nablus, Palestine; 8 Raymond and Beverly Sackler School of Physics and Astronomy, Tel Aviv University, Tel Aviv, Israel; 9 Department of Physics, Technion, Israel Institute of Technology, Haifa, Israel; 10 Department of Physics, New York University, New York, NY United States of America; 11 Departamento de Física, Pontificia Universidad Católica de Chile, Santiago, Chile; 12 INFN Gruppo Collegato di Udine, Sezione di Trieste, Udine, Italy; 13 ICTP, Trieste, Italy; 14 Institut für Physik, Universität Mainz, Mainz, Germany; 15 LAPP, Univ. Savoie Mont Blanc, CNRS/IN2P3, Annecy, France; 16 AGH University of Krakow, Faculty of Physics and Applied Computer Science, Krakow, Poland; 17 Department of Physics, University of Toronto, Toronto, ON Canada; 18 Department of Physics, Brandeis University, Waltham, MA United States of America; 19 Department of Physics, Northern Illinois University, DeKalb, IL United States of America; 20 Department of Physics, Istanbul University, Istanbul, Turkey; 21 Département de Physique Nucléaire et Corpusculaire, Université de Genève, Genève, Switzerland; 22 Particle Physics Department, Rutherford Appleton Laboratory, Didcot, United Kingdom; 23 Santa Cruz Institute for Particle Physics, University of California Santa Cruz, Santa Cruz, CA United States of America; 24 CERN, Geneva, Switzerland; 25 Institut de Física d’Altes Energies (IFAE), Barcelona Institute of Science and Technology, Barcelona, Spain; 26 INFN Sezione di Pavia, Pavia, Italy; 27 Dipartimento di Fisica, Università di Pavia, Pavia, Italy; 28 Department of Physics, Alexandru Ioan Cuza University of Iasi, Iasi, Romania; 29 Departamento de Física Teórica y del Cosmos, Universidad de Granada, Granada, Spain; 30 Affiliated with an international laboratory covered by a cooperation agreement with CERN, Geneva, Switzerland; 31 Department of Physics, McGill University, Montreal, QC Canada; 32 Department of Physics, Royal Holloway University of London, Egham, United Kingdom; 33 Deutsches Elektronen-Synchrotron DESY, Hamburg and Zeuthen, Germany; 34 INFN Sezione di Roma Tor Vergata, Rome, Italy; 35 Dipartimento di Fisica, Università di Roma Tor Vergata, Roma, Italy; 36 Department of Particle Physics and Astrophysics, Weizmann Institute of Science, Rehovot, Israel; 37 Fysiska institutionen, Lunds universitet, Lund, Sweden; 38 Affiliated with an institute covered by a cooperation agreement with CERN, Geneva, Switzerland; 39 Nevis Laboratory, Columbia University, Irvington, NY United States of America; 40 INFN Sezione di Bologna, Bologna, Italy; 41 Department of Physics and Astronomy, University of Victoria, Victoria, BC Canada; 42 INFN e Laboratori Nazionali di Frascati, Frascati, Italy; 43 Instituto de Física La Plata, Universidad Nacional de La Plata and CONICET, La Plata, Argentina; 44 SUPA - School of Physics and Astronomy, University of Edinburgh, Edinburgh, United Kingdom; 45 Horia Hulubei National Institute of Physics and Nuclear Engineering, Bucharest, Romania; 46 Physics Department, National Technical University of Athens, Zografou, Greece; 47 Nikhef National Institute for Subatomic Physics and University of Amsterdam, Amsterdam, Netherlands; 48 Czech Technical University in Prague, Prague, Czech Republic; 49 Institute of Physics, Academia Sinica, Taipei, Taiwan; 50 INFN Sezione di Milano, Milan, Italy; 51 Department of Physics and Astronomy, University of Sussex, Brighton, United Kingdom; 52 School of Physics and Astronomy, University of Birmingham, Birmingham, United Kingdom; 53 INFN Sezione di Napoli, Napoli, Italy; 54 Dipartimento di Fisica, Università di Napoli, Napoli, Italy; 55 Department of Physics, University of Washington, Seattle, WA United States of America; 56 Departamento de Física Teorica C-15 and CIAFF, Universidad Autónoma de Madrid, Madrid, Spain; 57 Universidade Federal do Rio De Janeiro COPPE/EE/IF, Rio de Janeiro, Brazil; 58 Fakultät für Physik, Ludwig-Maximilians-Universität München, München, Germany; 59 Department of Physics, University of Michigan, Ann Arbor, MI United States of America; 60 Laboratório de Instrumentação e Física Experimental de Partículas - LIP, Lisboa, Portugal; 61 Instituto de Física Corpuscular (IFIC), Centro Mixto Universidad de Valencia - CSIC, Valencia, Spain; 62 Department of Physics, University of Oslo, Oslo, Norway; 63 Department of Physics and Astronomy, University of Sheffield, Sheffield, United Kingdom; 64 IRFU, CEA, Université Paris-Saclay, Gif-sur-Yvette, France; 65 Department of Physics, University of Texas at Austin, Austin, TX United States of America; 66 Albert Einstein Center for Fundamental Physics and Laboratory for High Energy Physics, University of Bern, Bern, Switzerland; 67 Department of Physics, Stockholm University, Stockholm, Sweden; 68 Oskar Klein Centre, Stockholm, Sweden; 69 Dipartimento di Fisica, Università di Milano, Milano, Italy; 70 Physics Department, National and Kapodistrian University of Athens, Athens, Greece; 71 INFN Sezione di Pisa, Pisa, Italy; 72 Department of Physics, Oklahoma State University, Stillwater, OK United States of America; 73 Physics Division, Lawrence Berkeley National Laboratory, Berkeley, CA United States of America; 74 Institute for Nuclear Research and Nuclear Energy (INRNE) of the Bulgarian Academy of Sciences, Sofia, Bulgaria; 75 INFN Sezione di Roma, Roma, Italy; 76 KEK, High Energy Accelerator Research Organization, Tsukuba, Japan; 77 Institut für Physik, Humboldt Universität zu Berlin, Berlin, Germany; 78 Departamento de Engenharia Elétrica, Universidade Federal de Juiz de Fora (UFJF), Juiz de Fora, Brazil; 79 Department of Physics, Duke University, Durham, NC United States of America; 80 Oliver Lodge Laboratory, University of Liverpool, Liverpool, United Kingdom; 81 Group of Particle Physics, University of Montreal, Montreal, QC Canada; 82 Physikalisches Institut, Albert-Ludwigs-Universität Freiburg, Freiburg, Germany; 83 Dipartimento di Fisica, Sapienza Università di Roma, Roma, Italy; 84 Graduate School of Science and Kobayashi-Maskawa Institute, Nagoya University, Nagoya, Japan; 85 Ochanomizu University, Otsuka, Bunkyo-ku, Tokyo, Japan; 86 International Center for Elementary Particle Physics and Department of Physics, University of Tokyo, Tokyo, Japan; 87 Laboratory for Particle Physics and Cosmology, Harvard University, Cambridge, MA United States of America; 88 Department of Physics and Astronomy, University of Uppsala, Uppsala, Sweden; 89 LPMR, Faculté des Sciences, Université Mohamed Premier, Oujda, Morocco; 90 Faculty of Mathematics, Physics and Informatics, Comenius University, Bratislava, Slovak Republic; 91 Department of Physics, University of Cape Town, Cape Town, South Africa; 92 L2IT, Université de Toulouse, CNRS/IN2P3, UPS, Toulouse, France; 93 Department of Physics, University of Illinois, Urbana, IL United States of America; 94 Institute of Frontier and Interdisciplinary Science and Key Laboratory of Particle Physics and Particle Irradiation (MOE), Shandong University, Qingdao, China; 95 Fakultät für Mathematik und Naturwissenschaften, Fachgruppe Physik, Bergische Universität Wuppertal, Wuppertal, Germany; 96 Department of Physics, Nanjing University, Nanjing, China; 97 Department of Physics, Aristotle University of Thessaloniki, Thessaloniki, Greece; 98 Department of Physics, Carleton University, Ottawa, ON Canada; 99 Department of Physics, Yale University, New Haven, CT United States of America; 100 Institute of Physics, University of Belgrade, Belgrade, Serbia; 101 Department of Physics, University of Texas at Arlington, Arlington, TX United States of America; 102 School of Physics, University of Sydney, Sydney, Australia; 103 Charles University, Faculty of Mathematics and Physics, Prague, Czech Republic; 104 Kirchhoff-Institut für Physik, Ruprecht-Karls-Universität Heidelberg, Heidelberg, Germany; 105 Cavendish Laboratory, University of Cambridge, Cambridge, United Kingdom; 106 Institute of Nuclear Physics Polish Academy of Sciences, Krakow, Poland; 107 Department of Physics and Astronomy, University of Pittsburgh, Pittsburgh, PA United States of America; 108 Physikalisches Institut, Universität Bonn, Bonn, Germany; 109 School of Physics, University of Melbourne, Victoria, Australia; 110 Dipartimento di Fisica, Università di Genova, Genova, Italy; 111 INFN Sezione di Genova, Genova, Italy; 112 Department of Physics and Astronomy, University College London, London, United Kingdom; 113 Max-Planck-Institut für Physik (Werner-Heisenberg-Institut), München, Germany; 114 Institute for Fundamental Science, University of Oregon, Eugene, OR United States of America; 115 SLAC National Accelerator Laboratory, Stanford, CA United States of America; 116 Palacký University, Joint Laboratory of Optics, Olomouc, Czech Republic; 117 School of Physics and Astronomy, University of Manchester, Manchester, United Kingdom; 118 Department of Modern Physics and State Key Laboratory of Particle Detection and Electronics, University of Science and Technology of China, Hefei, China; 119 Department of Physics, Oxford University, Oxford, United Kingdom; 120 Institute of High Energy Physics, Chinese Academy of Sciences, Beijing, China; 121 Physics Department, Tsinghua University, Beijing, China; 122 University of Chinese Academy of Science (UCAS), Beijing, China; 123 Physics Department, Lancaster University, Lancaster, United Kingdom; 124 Fakultät Physik, Technische Universität Dortmund, Dortmund, Germany; 125 INFN Sezione di Roma Tre, Roma, Italy; 126 Dipartimento di Matematica e Fisica, Università Roma Tre, Roma, Italy; 127 IJCLab, Université Paris-Saclay, CNRS/IN2P3, 91405 Orsay, France; 128 SUPA - School of Physics and Astronomy, University of Glasgow, Glasgow, United Kingdom; 129 Department Physik, Universität Siegen, Siegen, Germany; 130 Department of Physics, Bogazici University, Istanbul, Turkey; 131 LPNHE, Sorbonne Université, Université Paris Cité, CNRS/IN2P3, Paris, France; 132 Department of Physics and Astronomy, Tufts University, Medford, MA United States of America; 133 Department of Physics, University of Warwick, Coventry, United Kingdom; 134 Istinye University, Sariyer, Istanbul, Turkey; 135 Departments of Physics and Astronomy, Stony Brook University, Stony Brook, NY United States of America; 136 Rio de Janeiro State University, Rio de Janeiro, Brazil; 137 Technical University of Munich, Munich, Germany; 138 United Arab Emirates University, Al Ain, United Arab Emirates; 139 Faculté des Sciences Ain Chock, Réseau Universitaire de Physique des Hautes Energies - Université Hassan II, Casablanca, Morocco; 140 Department of Physics, University of Arizona, Tucson, AZ United States of America; 141 APC, Université Paris Cité, CNRS/IN2P3, Paris, France; 142 Institut für Kern- und Teilchenphysik, Technische Universität Dresden, Dresden, Germany; 143 School of Physics and Astronomy, Queen Mary University of London, London, United Kingdom; 144 Department of Mechanical Engineering Science, University of Johannesburg, Johannesburg, South Africa; 145 Fakultät für Physik und Astronomie, Julius-Maximilians-Universität Würzburg, Würzburg, Germany; 146 High Energy Physics Division, Argonne National Laboratory, Argonne, IL United States of America; 147 Department of Physics Engineering, Gaziantep University, Gaziantep, Türkiye; 148 Dipartimento di Fisica e Astronomia A. Righi, Università di Bologna, Bologna, Italy; 149 Department of Physics, University of Wisconsin, Madison, WI United States of America; 150 LPC, Université Clermont Auvergne, CNRS/IN2P3, Clermont-Ferrand, France; 151 Ohio State University, Columbus, OH United States of America; 152 School of Physics and Astronomy, Shanghai Jiao Tong University, Key Laboratory for Particle Astrophysics and Cosmology (MOE), SKLPPC, Shanghai, China; 153 Tsung-Dao Lee Institute, Shanghai, China; 154 Departamento de Física, Universidad Técnica Federico Santa María, Valparaíso, Chile; 155 Department of Subnuclear Physics, Institute of Experimental Physics of the Slovak Academy of Sciences, Kosice, Slovak Republic; 156 Department for Physics and Technology, University of Bergen, Bergen, Norway; 157 Department of Physics, Simon Fraser University, Burnaby, BC Canada; 158 Department of Physics, Boston University, Boston, MA United States of America; 159 Department of Physics and Astronomy, Michigan State University, East Lansing, MI United States of America; 160 II. Physikalisches Institut, Justus-Liebig-Universität Giessen, Giessen, Germany; 161 Department of Physics, Ankara University, Ankara, Turkey; 162 Department of Physics, Indiana University, Bloomington, IN United States of America; 163 Dipartimento di Fisica E. Fermi, Università di Pisa, Pisa, Italy; 164 Niels Bohr Institute, University of Copenhagen, Copenhagen, Denmark; 165 University of Iowa, Iowa City, IA United States of America; 166 Dipartimento di Fisica, Università della Calabria, Rende, Italy; 167 INFN Gruppo Collegato di Cosenza, Laboratori Nazionali di Frascati, Frascati, Italy; 168 TRIUMF, Vancouver, BC Canada; 169 Institute for Mathematics, Astrophysics and Particle Physics, Radboud University/Nikhef, Nijmegen, Netherlands; 170 School of Physics, University of the Witwatersrand, Johannesburg, South Africa; 171 Departamento de Física, Universidade do Minho, Braga, Portugal; 172 INFN Sezione di Lecce, Lecce, Italy; 173 Dipartimento di Matematica e Fisica, Università del Salento, Lecce, Italy; 174 High Energy Physics Institute, Tbilisi State University, Tbilisi, Georgia; 175 Department of Physics, Stanford University, Stanford CA, United States of America; 176 Department of Physics, Chinese University of Hong Kong, Shatin, N.T., Hong Kong, China; 177 Department of Physics, National Tsing Hua University, Hsinchu, Taiwan; 178 Institute of Physics of the Czech Academy of Sciences, Prague, Czech Republic; 179 Department of Experimental Particle Physics, Jožef Stefan Institute and Department of Physics, University of Ljubljana, Ljubljana, Slovenia; 180 Dipartimento Politecnico di Ingegneria e Architettura, Università di Udine, Udine, Italy; 181 LPSC, Université Grenoble Alpes, CNRS/IN2P3, Grenoble INP, Grenoble, France; 182 Instituto Superior Técnico, Universidade de Lisboa, Lisboa, Portugal; 183 Department of Physics, University of British Columbia, Vancouver, BC Canada; 184 INFN-TIFPA, Povo, Italy; 185 Università degli Studi di Trento, Trento, Italy; 186 Department of Physics and Astronomy, University of California Irvine, Irvine, CA United States of America; 187 Physikalisches Institut, Ruprecht-Karls-Universität Heidelberg, Heidelberg, Germany; 188 Department of Physics, University of Pennsylvania, Philadelphia, PA United States of America; 189 Universidad de Buenos Aires, Facultad de Ciencias Exactas y Naturales, Departamento de Física, y CONICET, Instituto de Física de Buenos Aires (IFIBA), Buenos Aires, Argentina; 190 Department of Physics and Astronomy, York University, Toronto, ON Canada; 191 Physics Department, Southern Methodist University, Dallas, TX United States of America; 192 Millennium Institute for Subatomic physics at high energy frontier (SAPHIR), Santiago, Chile; 193 Instituto de Física, Universidade de São Paulo, São Paulo, Brazil; 194 Faculté des Sciences, Université Ibn-Tofail, Kénitra, Morocco; 196 National Centre for Scientific Research “Demokritos”, Agia Paraskevi, Greece; 197 Physics Department, University of Texas at Dallas, Richardson, TX United States of America; 198 Department of Physics, University of Adelaide, Adelaide, Australia; 199 Departamento de Física, Universidade de Coimbra, Coimbra, Portugal; 200 National Institute of Physics, University of the Philippines, Diliman (Philippines), Diliman, Philippines; 203 Enrico Fermi Institute, University of Chicago, Chicago, IL United States of America; 204 Marian Smoluchowski Institute of Physics, Jagiellonian University, Krakow, Poland; 205 Department of Physics, University of Alberta, Edmonton, AB Canada; 206 Departamento de Física, Faculdade de Ciências, Universidade de Lisboa, Lisboa, Portugal; 207 West University in Timisoara, Timisoara, Romania; 209 California State University, Long Beach, CA United States of America; 210 Department of Physics and Astronomy, University of New Mexico, Albuquerque, NM United States of America; 211 Division of Physics and Tomonaga Center for the History of the Universe, Faculty of Pure and Applied Sciences, University of Tsukuba, Tsukuba, Japan; 212 Department of Physics, Shinshu University, Nagano, Japan; 213 Department of Physics, Tokyo Institute of Technology, Tokyo, Japan; 214 Department of Physics and Astronomy, Iowa State University, Ames IA, United States of America; 215 Graduate School of Science, Kobe University, Kobe, Japan; 216 Graduate School of Science, Osaka University, Osaka, Japan; 217 University Politehnica Bucharest, Bucharest, Romania; 218 Department of Physics, University of Hong Kong, Hong Kong, China; 219 Institute of Physics, Azerbaijan Academy of Sciences, Baku, Azerbaijan; 220 Waseda University, Tokyo, Japan; 221 E. Andronikashvili Institute of Physics, Iv. Javakhishvili Tbilisi State University, Tbilisi, Georgia; 222 Instituto de Alta Investigación, Universidad de Tarapacá, Arica, Chile; 223 Research Center for Advanced Particle Physics and Department of Physics, Kyushu University, Fukuoka, Japan; 224 Universität Innsbruck, Department of Astro and Particle Physics, Innsbruck, Austria; 225 Faculty of Science, Kyoto University, Kyoto, Japan; 226 Universidad Andres Bello, Department of Physics, Santiago, Chile; 227 Department of Physics, Royal Institute of Technology, Stockholm, Sweden; 228 Department of Physics and Institute for Advanced Study, Hong Kong University of Science and Technology, Clear Water Bay, Kowloon, Hong Kong China; 229 Centro de Física Nuclear da Universidade de Lisboa, Lisboa, Portugal; 230 Facultad de Ciencias y Centro de Investigaciónes, Universidad Antonio Nariño, Bogotá, Colombia; 231 Transilvania University of Brasov, Brasov, Romania; 232 National Institute for Research and Development of Isotopic and Molecular Technologies, Physics Department, Cluj-Napoca, Romania; 233 Departamento de Física, Universidad Nacional de Colombia, Bogotá, Colombia; 234 Louisiana Tech University, Ruston, LA United States of America; 235 University of Sharjah, Sharjah, United Arab Emirates; 236 Division of Physics, TOBB University of Economics and Technology, Ankara, Turkey; 237 Kyoto University of Education, Kyoto, Japan; 238 Instituto de Investigación Multidisciplinario en Ciencia y Tecnología, y Departamento de Física, Universidad de La Serena, La Serena, Chile; 239 Affiliated with an institute covered by a cooperation agreement with CERN, Geneva, Switzerland; 240 Borough of Manhattan Community College, City University of New York, New York, NY United States of America; 241 Bruno Kessler Foundation, Trento, Italy; 242 Center for High Energy Physics, Peking University, Beijing, China; 243 Centro Studi e Ricerche Enrico Fermi, Roma, Italy; 244 CERN, Geneva, Switzerland; 245 Département de Physique Nucléaire et Corpusculaire, Université de Genève, Genève, Switzerland; 246 Departament de Fisica de la Universitat Autonoma de Barcelona, Barcelona, Spain; 247 Department of Financial and Management Engineering, University of the Aegean, Chios, Greece; 248 Department of Physics and Astronomy, Michigan State University, East Lansing MI, United States of America; 249 Department of Physics and Astronomy, University of Louisville, Louisville, KY United States of America; 250 Department of Physics, Ben Gurion University of the Negev, Beer Sheva, Israel; 251 Department of Physics, California State University, East Bay, United States of America; 252 Department of Physics, California State University, Sacramento, United States of America; 253 Department of Physics, King’s College London, London, United Kingdom; 254 Department of Physics, University of Fribourg, Fribourg, Switzerland; 255 Department of Physics, University of Thessaly, Thessaly, Greece; 256 Department of Physics, Westmont College, Santa Barbara, United States of America; 257 Hellenic Open University, Patras, Greece; 258 Institucio Catalana de Recerca i Estudis Avancats, ICREA, Barcelona, Spain; 259 Institut für Experimentalphysik, Universität Hamburg, Hamburg, Germany; 260 Institute of Particle Physics (IPP), Ottawa, Canada; 261 Institute of Physics, Azerbaijan Academy of Sciences, Baku, Azerbaijan; 262 Institute of Theoretical Physics, Ilia State University, Tbilisi, Georgia; 263 Lawrence Livermore National Laboratory, Livermore, United States of America; 264 The City College of New York, New York, NY United States of America; 265 The Collaborative Innovation Center of Quantum Matter (CICQM), Beijing, China; 266 TRIUMF, Vancouver, BC Canada; 267 Università di Napoli Parthenope, Napoli, Italy; 268 University of Chinese Academy of Sciences (UCAS), Beijing, China; 269 University of Colorado Boulder, Department of Physics, Colorado, United States of America; 270 Yeditepe University, Physics Department, Istanbul, Turkey

**Keywords:** Particle physics, Experimental particle physics

Correction to: *Nature* 10.1038/s41586-022-04893-w Published online 4 July 2022

In the version of this article initially published, the ATLAS Collaboration author names, affiliations and acknowledgements were omitted and have now been included in the HTML and PDF versions of the article.

